# A SAM analogue-utilizing ribozyme for site-specific RNA alkylation in living cells

**DOI:** 10.1038/s41557-023-01320-z

**Published:** 2023-09-04

**Authors:** Takumi Okuda, Ann-Kathrin Lenz, Florian Seitz, Jörg Vogel, Claudia Höbartner

**Affiliations:** 1 Institute of Organic Chemistry, Julius-Maximilians-Universität Würzburg, Würzburg, Germany; 2 Institute of Molecular Infection Biology (IMIB), Julius-Maximilians-Universität Würzburg, Würzburg, Germany; 3 Helmholtz Institute for RNA-based Infection Research (HIRI), Helmholtz Centre for Infection Research (HZI), Würzburg, Germany; 4 Center for Nanosystems Chemistry (CNC), Julius-Maximilians-Universität Würzburg, Würzburg, Germany

**Keywords:** RNA, Chemical modification

## Abstract

Post-transcriptional RNA modification methods are in high demand for site-specific RNA labelling and analysis of RNA functions. In vitro-selected ribozymes are attractive tools for RNA research and have the potential to overcome some of the limitations of chemoenzymatic approaches with repurposed methyltransferases. Here we report an alkyltransferase ribozyme that uses a synthetic, stabilized *S-*adenosylmethionine (SAM) analogue and catalyses the transfer of a propargyl group to a specific adenosine in the target RNA. Almost quantitative conversion was achieved within 1 h under a wide range of reaction conditions in vitro, including physiological magnesium ion concentrations. A genetically encoded version of the SAM analogue-utilizing ribozyme (SAMURI) was expressed in HEK293T cells, and intracellular propargylation of the target adenosine was confirmed by specific fluorescent labelling. SAMURI is a general tool for the site-specific installation of the smallest tag for azide-alkyne click chemistry, which can be further functionalized with fluorophores, affinity tags or other functional probes.

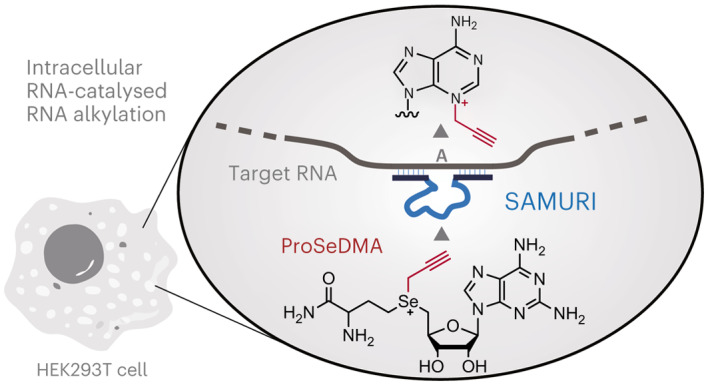

## Abstract (web summary)

Ribozyme-mediated post-transcriptional RNA modification is a powerful method for site-specific RNA labelling and analysis of RNA functions. Now, an alkyltransferase ribozyme—termed SAMURI—has been shown to catalyse the transfer of a propargyl group from a stabilized synthetic *S*-adenosylmethionine analogue to a specific adenosine on the target RNA within cells.

## Main

Site-specific chemical modification methods that enable precise post-synthetic or post-transcriptional functionalization of RNA provide robust means to probe and manipulate RNA functions in vitro and in cells^1,2^. Chemoenzymatic strategies with repurposed methyltransferase (MTase) protein enzymes and *S-*adenosylmethionine (SAM or AdoMet) derivatives have emerged as powerful methods for targeted RNA modification^3–6^. Several types of chemically modified SAM analogue have been developed, including variants for the transfer of alkyne^7,8^ or azide tags^9,10^, photoreactive groups^11^, fluorophores^12^ or biotin^13^. The installation of bioorthogonal functional groups allows further derivatization via click reactions for several purposes, including affinity purification^8^, chemical degradation^14^ or fluorescent labelling^15^ of target RNAs. In some examples, modified SAM analogues have been applied for target modification in the intracellular context by using a cascade reaction of methionine adenosyl-transferase (MAT) and MTases^16–18^. Such metabolic approaches are designed to give global modifications of DNA, RNA or proteins^16,19^, but often lack target specificity, because many cellular MTases maintain a common SAM recognition domain. To address specific RNA targets beyond the mRNA cap^20^, either structure-specific tRNA MTases^21^, or C/D box snoRNPs with sequence-specific guide RNAs^7^, have been used in vitro.

Ribozymes have gained increasing momentum as an alternative sequence-specific approach for post-synthetic RNA modification^2,22^. Usually, the catalytic core is flanked by binding arms that hybridize to a particular target sequence by Watson–Crick base-pairing, placing the target nucleotide into the active site for reaction with a specific, non-covalently bound cofactor. Thus, these *trans*-active ribozymes combine both functions—the guide and the catalyst—in one RNA molecule that interacts with the RNA target. Several combinations of ribozymes and cofactors for RNA labelling have been identified by in vitro selection. For example, ribozymes catalyse the attachment of fluorescently labelled nucleotides^23,24^ or acyclic nucleotide analogues^25^ to a specific internal 2′-OH group of the target RNA, or at the 3′-end^26^. Other classes of ribozymes work in *cis*, that is, they catalyse their own modification, for example, via self-alkylation with electrophilic iodoacetyl groups^27,28^ or reactive epoxide probes^29,30^. The most recent addition to the repertoire of RNA-catalysed reactions concerns site-specific RNA methylation. The in vitro-selected methyltransferase ribozyme MTR1 uses *O*^6^-methylguanine (m^6^G) as cofactor^31,32^, and a split riboswitch uses an engineered ligand *O*^6^-methyl pre-queuosine (m^6^preQ_1_)^33^ to methylate their RNA targets at the Watson–Crick base-pairing face to produce m^1^A (ref. ^31^) and m^3^C (ref. ^33^), respectively. Self-methylation at the Hoogsteen edge to install m^7^G was achieved by an in vitro-selected copper ion-dependent ribozyme that uses native SAM as cofactor^34^. In analogy to chemoenzymatic MTase engineering, these recently reported methyltransferase ribozymes have high potential to be repurposed for RNA labelling. However, only MTR1 has so far been addressed to a variety of RNA target sequences, including tRNAs, and m^6^preQ_1_- and SAM-dependent ribozymes suffer from a severely restricted sequence scope.

In this Article we report a ribozyme that catalyses the sequence-specific transfer of a propargyl group from bioorthogonal SAM analogues to the minor groove side of a specific adenine in various RNA target sequences. We explore the scope of the bioorthogonal SAM analogue-utilizing ribozyme and find that the catalytic activity is retained even at low Mg^2+^ concentrations. This feature enables the intracellular site-specific RNA modification upon expression of the ribozyme in living human cells.

## Results and discussion

### Cofactor design and synthesis

**Fig. 1:**
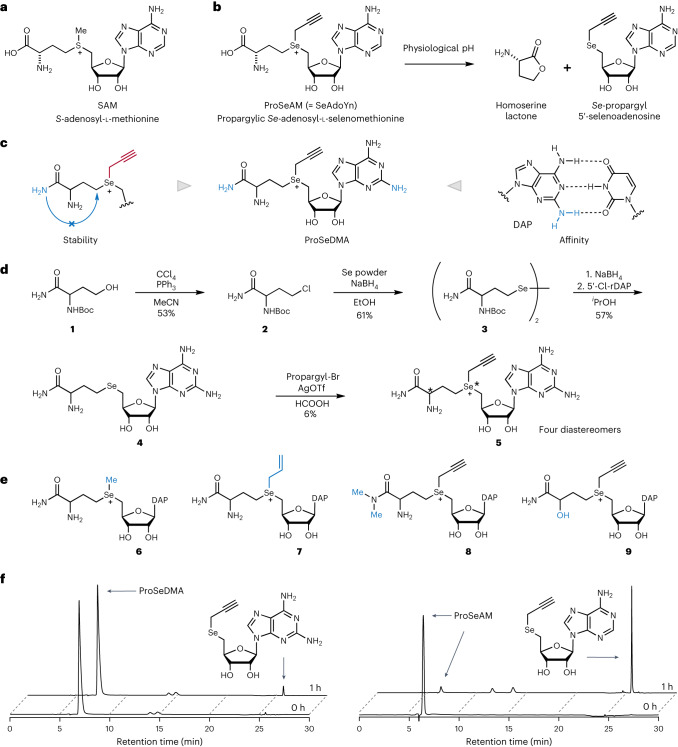
Design and characterization of ProSeDMA. **a**, Chemical structure of SAM. **b**, Major degradation pathway of ProSeAM. **c**, Concept of ProSeDMA. Terminal amide substitution inhibits self-degradation, and 2-aminoadenosine may assist binding to a ribozyme. DAP = 2,6-diaminopurine. **d**, Synthesis of ProSeDMA. The asterisk indicates a stereocentre. **e**, Other SeDMA analogues synthesized. **f**, Stability comparison between ProSeDMA (left) and ProSeAM (right). 1 mM cofactor was incubated in 50 mM HEPES (pH 7.0), 120 mM KCl, 5 mM NaCl and 10 mM MgCl_2_ at 37 °C. Aliquots were analysed by RP–HPLC, monitored at 260 nm.

Synthetic derivatives of the natural cofactor SAM (Fig. 1a) carrying allyl, propargyl and further extended groups at the sulfonium centre have been shown to be accepted by various types of MTase for alkylating DNA, RNA, proteins and small molecules^35,36^. Double-activated cofactor analogues contain an unsaturated bond at the β carbon to stabilize the transition state by hyperconjugation and to rescue the reduced reaction rates compared to the transfer of a methyl group^37^. To further increase the reactivity for the nucleophilic attack, the sulfur centre was replaced by selenium, and propargylated adenosyl selenomethionine (ProSeAM, also known as SeAdoYn) has become a heavily investigated cofactor for MTase protein enzymes^38,39^. A similar approach using artificial SAM analogues would be attractive for alkyltransferase ribozymes, but the rather poor chemical stability of ProSeAM and its analogues limits their utility as reagents for in vitro selection of catalytic RNA. Several pathways contribute to the spontaneous decomposition in aqueous environment^40^. Propargylated SAM undergoes rapid hydration of the triple bond at physiological pH, probably via an allene intermediate, and the major decomposition pathway for ProSeAM at physiological pH involves an intramolecular nucleophilic attack of the carboxylate group, resulting in breaking the weak selenium–carbon bond^19^. This intramolecular lactonization of the 2-aminobutyrate moiety affords the by-products homoserine lactone and *Se*-propargyl-5′-selenoadenosine (Fig. 1b). To avoid this side reaction, we designed a more stable and bioorthogonal propargylic SAM analogue, which we termed ProSeDMA (for propargylic *Se*-2,6-diaminopurinribosyl-selenomethionineamide) (Fig. 1c). The nucleophilic carboxylate was replaced by an amide group to prevent the intramolecular S_N_2 reaction. In addition, 2-aminoadenosine (also known as 2,6-diaminopurine riboside, rDAP) was used as the nucleoside, with the aim to increase the binding affinity between cofactor and ribozyme by introducing an extra hydrogen-bond donor. The chemical synthesis of ProSeDMA involved four steps, starting from the protected α-aminoamide alcohol **1**, via transformation to the selenohomocystine amide **3** (Fig. 1d). After reduction of the diselenide, reaction with 5′-chloro-rDAP afforded the selenoether **4**. The propargyl group was installed by alkylation in the presence of a Lewis acid, and ProSeDMA (**5**) was purified by reversed-phase (RP) HPLC. Analogous cofactors with *Se*-methyl (MeSeDMA, **6**) and *Se*-allyl groups (AllSeDMA, **7**) were also prepared, as well as the *N*,*N*-dimethylamide **8**, in which the amide group of ProSeDMA was further blocked by alkylation (Fig. 1e). In addition, we prepared an analogous α-hydroxy-amide cofactor **9**, which we considered an alternative in vitro-selection substrate. Each of these cofactors was obtained as a mixture of four diastereomers, which were not separated.

The chemical stability of ProSeDMA was compared to ProSeAM (Fig. 1f) under three different pH conditions (pH 6, 7 and 8). ProSeAM was rapidly degraded even at pH 6, and almost all of it was converted to *Se*-propargyl-5′-selenoadenosine after 1 h. In contrast, ProSeDMA was much more stable, with less than 5% loss after 1 h, and still more than 60% of the intact material remaining after 5 h of incubation at pH 7.0.

**Extended Data Fig. 1:**
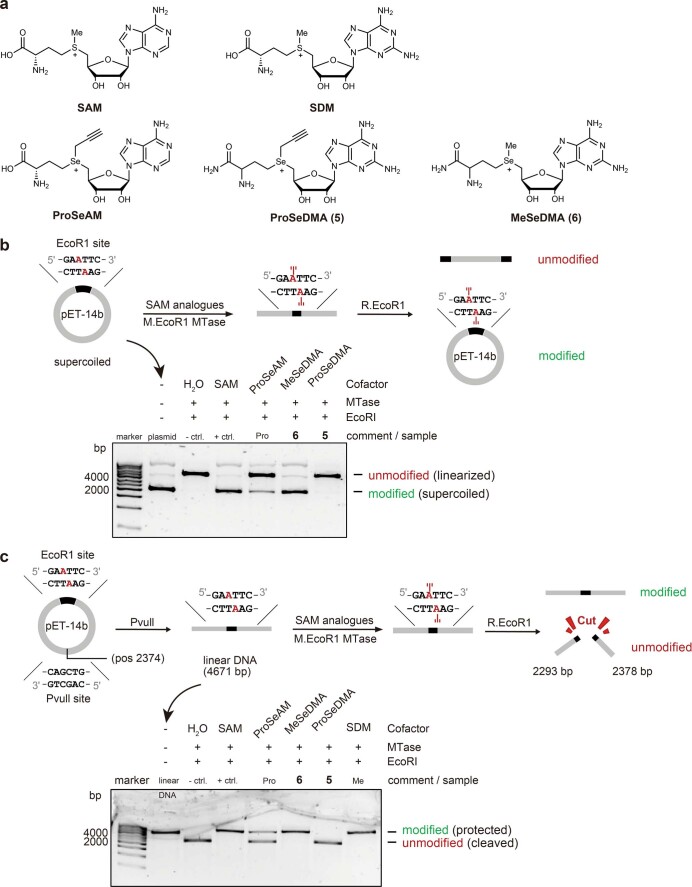
Enzymatic recognition of SAM analogues by MTase (M.EcoR1). **a**. Structures of cofactors used. **b**. Plasmid (pET-14b) was subjected to enzymatic modification with M.EcoRI and the respective cofactor, and the modification efficiency was analyzed by restriction enzyme R.EcoRI. **c**. Plasmid (pET-14b) was fist linearized by digestion with restriction enzyme PvuII, and then treated as in **b**. Transalkylation conditions: 150 ng Plasmid / linear DNA, 100 μM SAM analogue, 50 mM Tris-HCl, 50 mM NaCl, 10 mM EDTA (pH = 8.0), 40 U M.EcoR1 MTase 37 °C for 1 h. Then the MTase was inactivated by heating to 65 °C for 20 min. Restriction enzyme condition: 20 U R.EcoR1, 37 °C for 1 h. Analysis condition: 0.8% agarose gel (75 V, 45 min), staining: SYBR green. Representative images of two independent experiments with similar results are shown. Source data

Next, we investigated the bioorthogonality of ProSeDMA, expecting that the terminal amide substitution and 2-aminoadenosine modification probably prevent recognition by native MTases. We chose the bacterial MTase M.EcoR1 and plasmid DNA for enzymatic modification, as previously described^37^. The modification efficiency was evaluated by enzymatic digestion with the restriction enzyme R. EcoR1 after MTase treatment (Extended Data Fig. 1). If the cofactor is accepted and the target site is modified by MTase, digestion by the restriction enzyme will be suppressed. Analogous reactions with SAM, ProSeAM, ProSeDMA (**5**) and MeSeDMA (**6**) were performed in parallel. The digestion pattern of the ProSeDMA-containing sample was almost the same as in the experiment without cofactor. This result suggests that ProSeDMA was not recognized as a cofactor by EcoR1 MTase. By contrast, ProSeAM, and surprisingly also MeSeDMA, showed similar activity to SAM. Thus, the combination of propargyl and amide modification provided sufficient structural change to escape from MTase recognition, and ProSeDMA is a potential candidate to serve as a bioorthogonal cofactor for intracellular propargyl modification of RNA with a suitable ribozyme.

### In vitro selection of ProSeDMA utilizing ribozyme

**Fig. 2:**
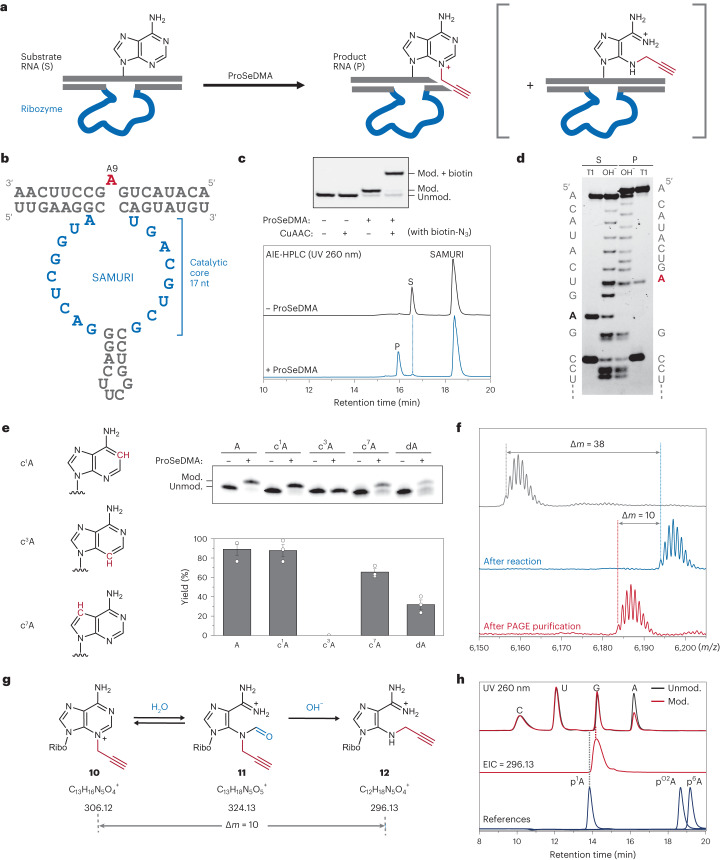
Identification and analysis of ProSeDMA utilizing ribozyme (SAMURI). **a**, Schematic of SAMURI-catalysed *trans*-propargylation to target adenosine. **b**, Sequence and predicted secondary structure of SAMURI. **c**, Polyacrylamide gel electrophoresis (PAGE, top) and anion-exchange HPLC analysis (bottom) of the SAMURI-catalysed reaction of 17-mer substrate RNA (S, 5′-ACAUACUGAGCCUUCAA-3′) with and without ProSeDMA at 37 °C for 1 h; 1 μM substrate RNA, 1 μM SAMURI, 10 μM ProSeDMA and 10 mM MgCl_2_, pH 7.5. PAGE also shows the reactivity test of transferred alkyne by CuAAC with biotin azide (10 μM mod. RNA, 500 μM CuBr, 1 mM TBTA and 1 mM biotin azide in H_2_O/DMSO/^*t*^BuOH = 5/3/1 at 37 °C for 1 h). AIE-HPLC = anion-exchange HPLC. **d**, Alkaline hydrolysis and RNase T1 digestion of reacted RNA (mod., product P) in comparison to untreated RNA (unmod., S). The image is representative of two independent experiments with similar results. **e**, Atomic mutagenesis of substrate RNA. Reaction conditions are as in **c**. A representative gel image is shown, and a histogram showing individual data points and the mean ± s.d. of three independent experiments. **f**, MALDI–TOF mass spectra of unmodified RNA (grey, *m*/*z* = 6,155.04), a mixture of modified RNA (blue, *m*/*z* = 6,193.06) and ribozyme, and modified RNA after PAGE purification (red, *m*/*z* = 6,183.08). **g**, Plausible reaction pathway causing a mass shift after purification. **h**, LC/MS analysis of the digested 12-mer substrate RNA (5′-CUACUGAGCCUU-3′) before and after modification, showing the UV (260 nm) trace and extracted ion chromatogram (EIC, detecting *m*/*z* = 296.13). p^1^A = 1-propargyladenosine, p^O2^A = 2′-*O*-propargyladenosine, p^6^A = *N*^6^-propargyladenosine. Source data

**Extended Data Fig. 2:**
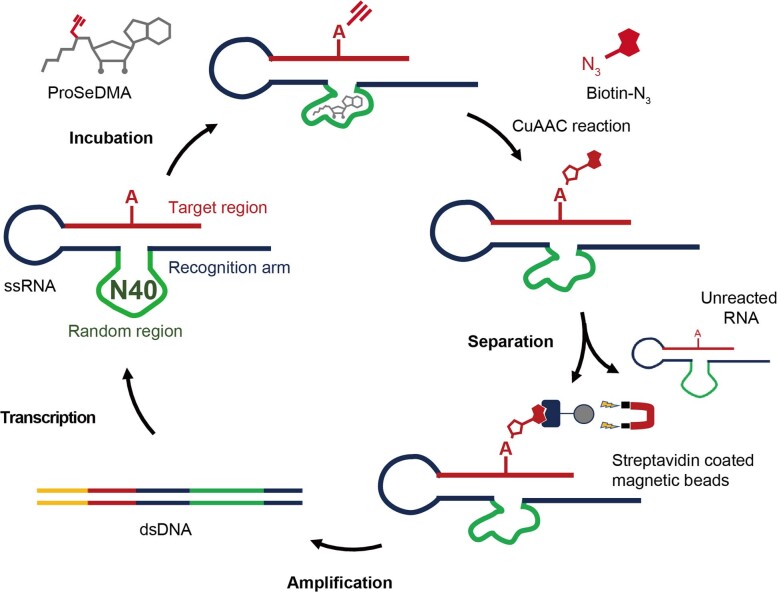
In vitro selection of trans-propargylation ribozymes. In vitro selection was proceeded by incubation, CuAAC reaction, separation, amplification and transcription steps. The RNA library contains an unpaired adenosine (red, A) and is connected to the 40 random nucleotides via the single-stranded loop. Incubation was done in 5 μM RNA, 5 μM cofactor **5** or **9**, 50 mM HEPES, pH 7.5, 120 mM KCl, 5 mM NaCl and 10 mM MgCl_2_, at 37 °C for 1 h. Then, propargylated RNA was modified with biotin in 100 μM RNA, 500 μM biotin azide, 1 mM CuBr and 2 mM TBTA. For capture, beads were blocked with E. coli tRNA; streptavidin and neutravidin beads were switched every two rounds. Denaturing wash buffer was 8 M urea, 10 mM Tris-HCl, pH 7.5, 1 mM EDTA, 0.01% Tween-20. Elution used 95% formamide, 1 mM EDTA, at 95 °C for 5 min. For RT–PCR, one-pot RT–PCR reaction was used with the following conditions: 42 °C, 30 min, 10 cycles of PCR with 1 μM primer **D5** and 0.5 μM primer **D3**. For the following PCR, we used: 30 cycles, 10% (v/v) RT–PCR product as template, 1 μM **D4** and 0.5 μM **D5**, 10% (v/v) DMSO, and an annealing temperature of 63 °C. For in vitro transcription, T7 RNA polymerase was used with dsDNA template obtained from a 200 μL PCR reaction, in 100 μL transcription reaction volume with 4 mM each NTP, 37 °C for 15 h, followed by PAGE purification.

**Extended Data Fig. 3:**
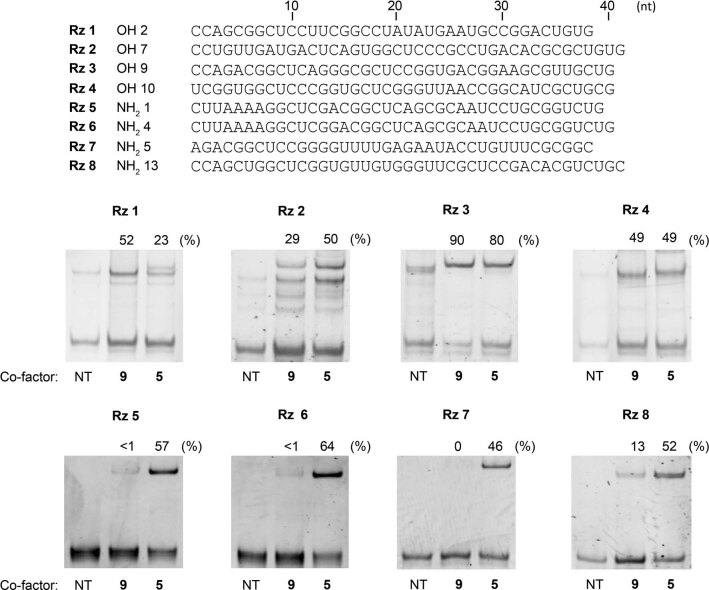
Identification of candidates of alkyltransferase ribozyme from in vitro selection libraries. Four sequences were identified from each library (OH series used cofactor **9**; NH_2_ series used cofactor **5** during selection). Trans alkylation activity was evaluated by streptavidin gel shift assay on native PAGE (the qualitative screening assay was performed once). Condition: 5 pmol RNA, 1 μg of streptavidin in 1x TBS buffer. Source data

**Extended Data Fig. 4:**
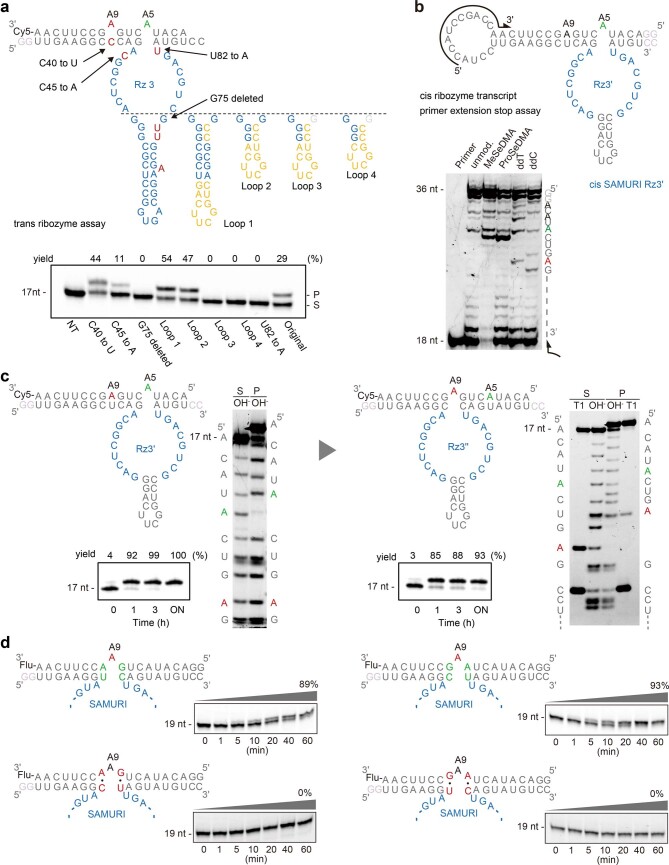
Sequence and predicted secondary structure of Rz3 ribozyme and its trans-active variants. **a**. Minimization and mutation analysis. Conditions for trans activity assay: 1 μM 3’-labelled substrate RNA, 1 μM **Rz3** or indicated mutant, 10 μM ProSeDMA and 10 mM MgCl_2_ were incubated at 37 °C for 1 h. **b**. Primer extension assay was performed after incubation of cis-Rz3’ transcript with ProSeDMA or MeSeDMA. Condition:10 pmol modified cis ribozyme, 10 pmol FAM labelled DNA primer, 1x first strand buffer (50 mM Tris-HCl pH8.3, 75 mM KCl and 3 mM MgCl_2_), 5 mM DTT, 0.5 mM of each dNTP and SuperScript III RT (ThermoFisherScientific) were incubated at 55 °C for 30 min. **c**. Transplanting the catalytic core to target an alternative adenosine (A9 instead of A5). Conditions as in (**a**) for 1 h, 3 h, or overnight (ON). NT, no treatment. Products (P) were isolated and subjected to alkaline hydrolysis. The shifted band in comparison to the alkaline hydrolysis pattern of the unmodified RNA (S) identified the modification site. **d**. SAMURI activity and sequence specificity with different base-pairs flanking the modification site. The mutated ribozymes and 19-nt long substrate RNAs were prepared by *in vitro* transcription. The substrate RNAs were labelled with fluorescein at the 3’-end after oxidation with periodate. SAMURI reaction conditions as in (**a**) with time points up to 1 h as indicated. Representative images are shown of two (**a**, **b**, **c**) or three (**d**) independent experiments with similar results. Source data

Alkyltransferase ribozymes for use with ProSeDMA were identified by in vitro selection from a structured RNA library that contained a random region of 40 nucleotides between a pair of recognition arms complementary to the RNA substrate region, leaving a single adenosine unpaired in the middle of the target RNA sequence (Fig. 2a and Extended Data Fig. 2)^23,31^. Two independent selection experiments were performed using ProSeDMA (**5**) and its α-hydroxy analogue **9** as co-substrates. After RNA folding and incubation with the cofactor at pH 7.5 and 37 °C for 1 h, the transferred propargyl group was reacted with biotin azide under Cu-catalysed azide-alkyne cycloaddition (CuAAC) reaction conditions. The biotinylated RNA molecules were then separated via streptavidin affinity purification on magnetic beads. The captured RNAs were amplified by reverse transcription and polymerase chain reaction (PCR). The generated dsDNA template was transcribed by T7 RNA polymerase, and the enriched RNA library was used in the next round of selection. After eight rounds, the enriched library was cloned and sequenced, and individual ribozyme candidates were screened for activity by a streptavidin gel shift assay (Extended Data Fig. 3) with both cofactors **5** and **9**. Interestingly, the ribozyme candidates from the selection with cofactor **5** only recognized their cognate α-amino cofactor ProSeDMA, while candidate ribozymes from the selection with cofactor **9** showed promiscuous activity with both **5** and **9**. The RNA sequence **Rz3** showed the highest reactivity of all screened ribozyme candidates, and was chosen for further characterization and optimization. The **Rz3** ribozyme retained its catalytic activity in an intermolecular set-up (in *trans*). Strategic mutations in the binding arms that fixed mismatched base pairs or bulged out nucleotides indicated the localization of the reacting adenosine (A5), which was further confirmed by primer extension assays and by re-targeting (‘transplanting’) the ribozyme to a different adenosine (A9) in the RNA sequence (Extended Data Fig. 4). Full activity was maintained when the base pairs flanking the modification site were exchanged, but mismatches were not tolerated (Extended Data Fig. 4d). The predicted stem loop in the ribozyme core was stabilized by a UUCG tetraloop. The engineered ribozyme containing a 17-nt catalytic core was named SAMURI (for SAM analogue-utilizing ribozyme) (Fig. 2b).

### Characterization of SAMURI-catalysed RNA alkylation

For detailed characterization, SAMURI was hybridized to the target RNA with two 8-nt binding arms and showed over 90% *trans*-propargylation efficiency after 1-h incubation at 37 °C with 10 µM ProSeDMA. The modified RNA was separable from the unmodified RNA substrate by anion-exchange HPLC and denaturing PAGE (Fig. 2c). The reacted adenosine was localized at A9, as confirmed by the shifted band pattern in alkaline hydrolysis and RNase T1 digestion assays (Fig. 2d). The precise modification site on A9 was then examined by atomic mutagenesis. Adenosine has several possible nucleophilic positions (N1, N3, N6, N7 and the 2′-OH group), which were individually addressed by the respective deaza or deoxy nucleosides (Fig. 2e). The substrate RNAs with 1-deazaadenosine (c^1^A) or 7-deazaadenosine (c^7^A) showed similar reactivity to unmodified adenosine. The RNA with 2′-deoxyadenosine (dA) showed two products of lower intensity (31% and 24%). By contrast, the RNA containing 3-deazaadenosine (c^3^A) did not give any modified product. These results strongly suggest that the N3 nitrogen is essential for catalytic activity, whereas N1 and N7 are dispensable.

**Extended Data Fig. 5:**
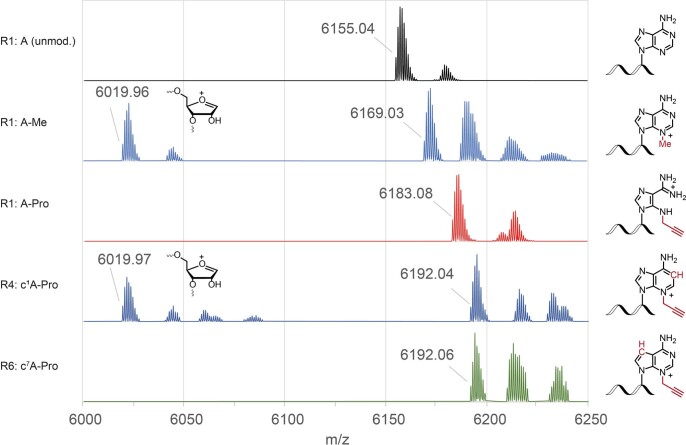
Mass spectrometric analysis of alkylated RNA products including atomic mutagenesis. ESI-MS of PAGE-purified 17-nt RNAs (5’-ACAUACUGAGCCUUCAA-Cy5); deconvoluted mass spectra are shown; Chemical formula and calculated masses are given in the Supporting Information (Supplementary Table 3).

Alkylation of adenosine at N3 in DNA is known to cause depurination^41^, and the second product observed with the dA-containing substrate is consistent with the formation of an abasic site. This mechanism is exploited by DNA targeting natural products and minor groove-binding cytotoxic agents^42^. In contrast, N3 alkylation of adenosine in RNA has barely been investigated, despite its biological importance^43^ and the mechanistic involvement of adenine-N3 in natural ribozymes^44^. Thus, to further characterize the product produced by SAMURI, the modified RNA was analysed by MS (Fig. 2f). The addition of a propargyl group to the target RNA (+38 Da) was detected by matrix-assisted laser desorption/ionization-time of flight (MALDI–TOF) mass analysis when the modified RNA was directly measured after the reaction. Surprisingly, after purification by denaturing PAGE, the mass spectrum of the modified RNA showed a loss of 10 Da. This RNA could still undergo CuAAC with biotin azide, confirming that the propargyl group was still intact (Fig. 2c). Interestingly, the propargylated RNAs containing c^1^A and c^7^A showed the expected mass of +38 Da, but not the +28 Da product that was observed for the parent adenosine (Extended Data Fig. 5). Instead, the electrospray ionization (ESI)-MS trace of the alkylated c^1^A sample showed a depurinated fragment, which was not seen for the c^7^A sample. Combining the results of atomic mutagenesis and mass analysis, a plausible modification process for adenosine is presented in Fig. 2g. SAMURI-catalysed alkylation of the N3 nitrogen enhances the electrophilicity of C2 for the attack of water, which results in ring opening followed by deformylation to produce a stable propargylated aminoimidazole nucleoside as product. The absence of the N1 nitrogen in c^1^A prevents ring opening, but results in a weaker glycosidic bond, while the missing N7 nitrogen in c^7^A prevents depurination.

To further confirm these mechanistic hypotheses, the PAGE-purified propargylated RNA was digested with snake venom phosphodiesterase (SVPD) and alkaline phosphatase enzymes, and the mononucleoside mixture was analysed by liquid chromatography-mass spectrometry (LC-MS; Fig. 2h). Interestingly, no new peak was detected in the UV chromatogram at 260 nm in addition to the standard four nucleosides, but the intensity of the adenosine peak was decreased. This result suggested that, upon reaction, the absorption of adenosine at 260 nm was reduced or lost, which is consistent with the proposed imidazole riboside depicted in Fig. 2g. Indeed, the extracted ion chromatogram (EIC) detected nucleoside **12** at 14 min (*m*/*z* 296.13), which was overlapped with the guanosine peak. The masses of the direct N3-alkylation product **10** (*m*/*z* 306.12) and the formyl intermediate **11** were not found after the digestion. For reference, we also recorded LC-MS spectra of stable isomers of 3-propargyladenosine **10**, namely 1-propargyladenosine (p^1^A), 2′-*O*-propargyladenosine (p^O2^A) and *N*^6^-propargyladenosine (p^6^A), which showed the expected EIC at *m*/*z* 306.12 at three different retention times. Based on the combined results of atomic mutagenesis and MS, we conclude that SAMURI catalyses the transfer of the propargyl group from ProSeDMA to the N3 nitrogen of the unpaired adenosine A9 in the target RNA.

### Kinetics and substrate scope of SAMURI

**Fig. 3:**
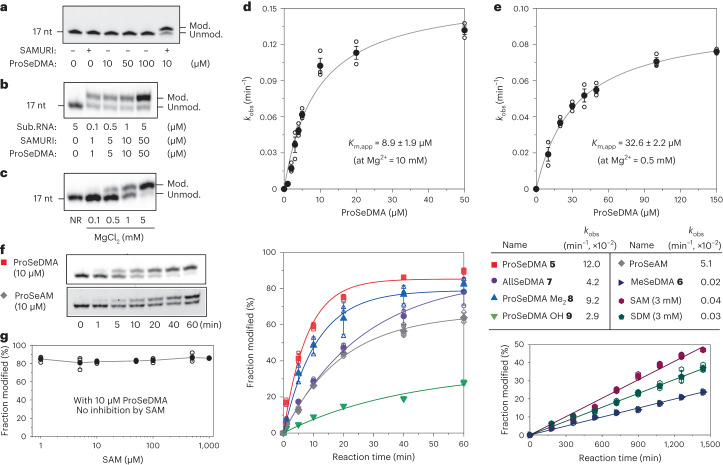
Characterization of SAMURI. **a**, Evaluation of unspecific modification by ProSeDMA. In the absence of ribozyme, 1 μM RNA substrate was incubated with an increasing concentration of ProSeDMA at 37 °C for 1 h. **b**, Catalytic activity of SAMURI under varying conditions. The ratio of RNA:SAMURI:ProSeDMA was kept at 1:10:10. Sub.RNA = substrate RNA. **c**, Mg^2+^ dependence of SAMURI (1 µM RNA, 1 µM SAMURI, 10 µM ProSeDMA, varying Mg^2+^ as indicated, 37 °C, 1 h). NR = no reaction. **d**,**e**, ProSeDMA concentration dependence of the observed rate constant *k*_obs_ at 10 mM Mg^2+^ (**d**) and 0.5 mM Mg^2+^ (**e**). Data are fitted to the Michaelis–Menten equation. **f**, Scope of SAMURI with various SAM analogues. *k*_obs_ values were obtained under pseudo-first-order reaction conditions for 60 min. For MeSeDMA, SAM and SDM, incubation was extended to 24 h, and a linear fit was applied because of the low reactivity. All cofactors were used at 10 µM, except for SAM and SDM, which were used at 3 mM. **g**, Inhibition assay: SAMURI *trans*-propargylation activity was not inhibited by SAM. Conditions are as in **c**, with increasing concentrations of SAM as indicated. **a**,**b**,**c**,**f** show representative images of three independent experiments, with similar results. In **d**,**e**,**g**, individual data points and the mean ± s.d. of three independent experiments are shown. Source data

Next, we evaluated the specificity and reactivity of SAMURI under various reaction conditions. To rule out uncatalysed background reactions, we incubated substrate RNA with ProSeDMA at concentrations up to 100 µM. In the absence of ribozyme, we did not observe any non-specific propargylation (Fig. 3a). Following the addition of SAMURI, the *trans*-propargylation was highly efficient, not only at the ProSeDMA concentration of 5 µM, which was used during in vitro selection, but also at the fivefold lower concentration of 1 μM (Fig. 3b), which still produced more than 60% product after 1 h incubation at 37 °C. With 10 µM ProSeDMA and 10 mM Mg^2+^, quantitative propargylation was achieved with an observed rate constant *k*_obs_ of 0.12 min^−1^. Importantly, SAMURI retained its catalytic activity at physiological Mg^2+^ concentrations (Fig. 3c). The reaction rate was dependent on ProSeDMA concentration, with an apparent *K*_m, app_ of 32 µM at 0.5 mM Mg^2+^, and 3.7-fold tighter binding at 10 mM Mg^2+^ (*K*_m, app_ = 8.9 µM) (Fig. 3d,e).

To explore the interactions between the cofactor and ribozyme, we evaluated the reactivity of different SeDMA derivatives (Fig. 3f). We found that alkylation of the terminal amide was tolerated well. Dimethylamide **8** reacted almost as efficiently as the parent cofactor **5**, suggesting that the terminal amide is not strictly recognized within the catalytic core. In contrast, replacement of the α-amino group by an α-hydroxyl group (cofactor **9**) decreased the activity of the ribozyme, suggesting that the protonation state of the α-amino group is important for interaction. Together, these results suggested that the selenomethionine amino-acid side chain of ProSeAM may also be tolerated. Indeed, 60% propargylation was observed with 10 µM ProSeAM within 1 h (Fig. 3f).

**Extended Data Fig. 6:**
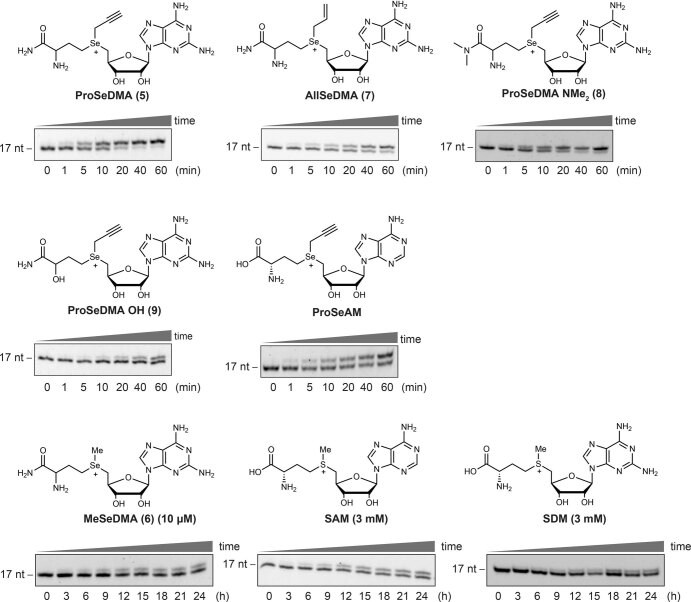
Scope of SAMURI trans activity. Gel images for kinetics graphs shown in Fig. 3f (ProSeDMA and ProSeAM gels are same as in Fig. 3f, here reproduced for comparison). Conditions: 10 pmol Cy5 labelled substrate RNA and 100 pmol SAMURI in reaction buffer (50 mM HEPES, 120 mM KCl and 5 mM NaCl); after annealing (3 min at 95 °C, 10 min at 25 °C), 10 mM MgCl_2_ and 100 pmol respective cofactor (or 30 nmol SAM) were added (total reaction volume 10 μL). Incubation at 37 °C, 1 μL aliquots mixed with 4 μL of stop solution; *k*_obs_ determined from pseudo-first order curve fit; Representative gel images of three independent replicates for all cofactors, except for MeSeDMA and SAM two replicates.

**Extended Data Fig. 7:**
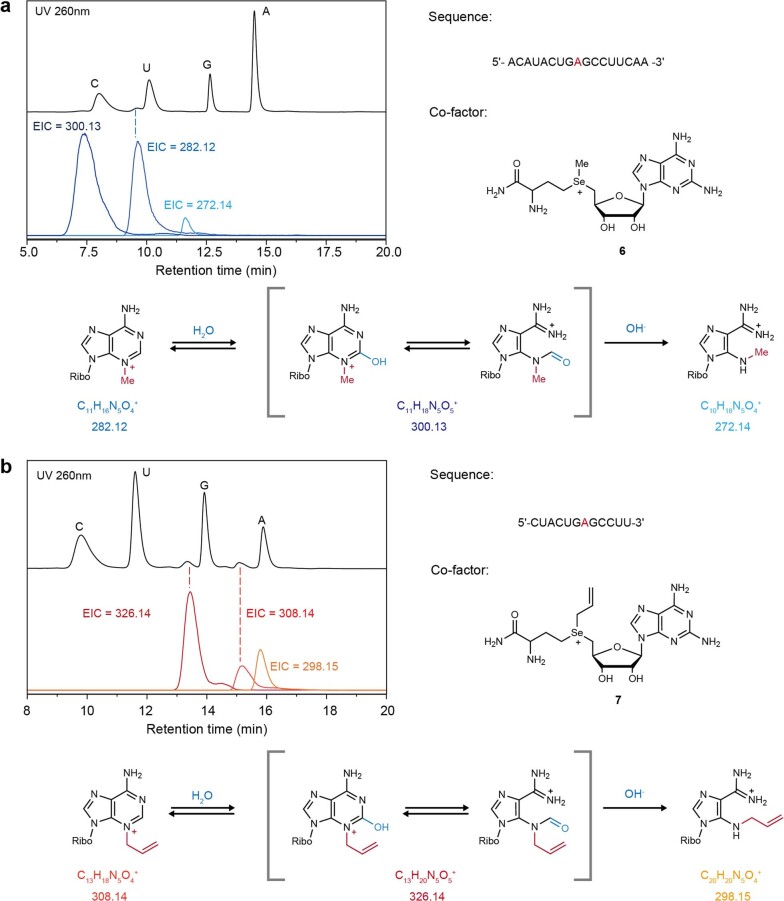
LC/MS analysis of digested RNA products containing methyl (a) and allyl (b) modification. a,b, After incubation with SAMURI and MeSeDMA or AllSeDMA, respectively, the RNAs were isolated by PAGE and then digested by bacterial alkaline phosphatase and snake venom phosphodiesterase (in 40 mM Tris-HCl, 20 mM MgCl_2_, pH 7.5). After extraction with chloroform, the aqueous layer was concentrated, and the residue was dissolved in 70 μL 5 mM NH_4_OAc and analyzed by LC-MS, using a Synergi Fusion RP HPLC column (Phenomenex, 4 μm, 250 × 2 mm). The analysis was run with a gradient of B from 0% - 5% (0 min to 15 min) and 5% - 72.5% (15 min to 45 min); solvent A was 10 mM NH_4_OAc (pH 5.3); solvent B was MeCN; flow rate was 0.2 mL/min at 25 °C with UV detection at 260 nm and ESI mass spectrometry in positive-ion mode.

Next, we examined the scope of alkyl groups that can be transferred by SAMURI. Allyl derivative **7**, containing an *sp*^2^ carbon in the β position to selenium, showed similar reactivity to ProSeDMA (Fig. 3f and Extended Data Fig. 6). On the other hand, the reaction with methyl derivative **6** was slowed down, indicating that the electrophilicity of the α carbon is dominant over the steric demand of the transferred alkyl group. At the standard concentration of 10 µM MeSeDMA, ~20% methylated RNA product was formed after 24 h. The methylated and allylated RNAs were confirmed by MS of intact RNA, and by LC-MS after digestion of the products into mononucleosides. Interestingly, we found differences in the stability of the N3-alkylated adenosines. In both cases of methyl or allyl modification, intact N3-alkylated adenosine was observed in addition to the hydrated and deformylated products (Extended Data Fig. 7).

Although the methylation rate with MeSeDMA was ~600-fold slower than propargylation with ProSeDMA, the above results suggested that native SAM could also be a productive cofactor for SAMURI. Indeed, methylated RNA product was observed upon incubation of the target RNA with SAMURI and SAM; however, compared to MeSeDMA, a much higher concentration of 3 mM SAM was required to reach comparable methylation levels. Similarly, the 2,6-diaminopurine analogue of SAM (called SDM) showed comparable RNA methylation levels when used at 3 mM (Fig. 3f and Extended Data Fig. 6). Consistent with the weak affinity, SAM was not able to compete with ProSeDMA in an inhibition assay. When the *trans*-propargylation reaction was performed with SAMURI and 10 µM ProSeDMA in the presence of increasing SAM concentration from 1 µM up to 1 mM, the yield of alkylated RNA product remained constant at the high level above 80% in 1 h (Fig. 3g). From these results we conclude that SAM is not a competitive inhibitor of SAMURI, which is an important finding for intracellular applications. In addition, we note that SAMURI enables the site-specific installation of m^3^A in RNA, a modification that is challenging to synthesize chemically^45^ and that has not yet been incorporated by solid-phase synthesis.

### Intracellular activity of SAMURI

**Fig. 4:**
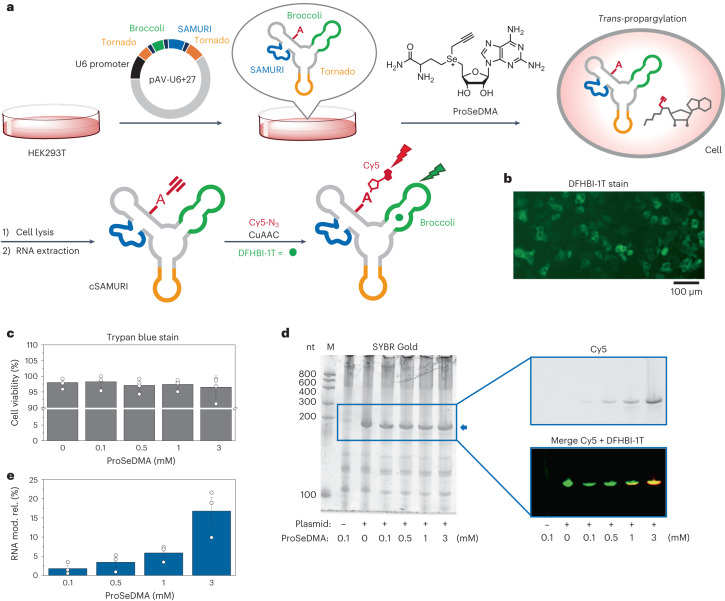
SAMURI activity in living cells. **a**, Schematic illustration of genetically encoded *cis*-active SAMURI. HEK293T cells grown in a cell culture dish were transfected with plasmid, and the cSAMURI-expressing cells were treated with ProSeDMA. The intracellularly propargylated RNA was isolated and labelled with Cy5 azide. The modification yield was quantified based on the fluorescence intensities of Cy5 and DFHBI-1T. **b**, Transfected cells stained with DFHBI-1T show expression of cSAMURI. **c**, Cell viability measured by trypan blue staining. Individual data points and the mean ± s.d. of four independent experiments are shown. **d**, Total RNA analysis on PAGE. The gel was visualized in the Cy5 channel, then stained with 20 μM DFHBI-1T and then stained by SYBR Gold. **e**, Intracellular labelling efficiency of cSAMURI calculated from the Cy5/DFHBI-1T ratio, relative to the in vitro labelling efficiency of RNA extracted from cells that were not treated with ProSeDMA. The reference was prepared in the test tube using 200 ng total RNA, 50 μM ProSeDMA and 10 mM MgCl_2_, pH 7.5 at 37 °C for 1 h. Individual data points and the mean ± s.d. of three independent experiments are shown. RNA mod. rel. = relative intracellular RNA modification efficiency. Source data

The findings that SAMURI was still active at low Mg^2+^ concentrations and was not inhibited by SAM encouraged the investigation of RNA-catalysed RNA propargylation in living cells. To explore this possibility, we designed plasmids for the expression of SAMURI in human cells. The *cis*-active SAMURI was placed in the F30 scaffold^46^ together with the fluorogenic aptamer Broccoli^47^ and the Tornado system for the production of circular RNA (cSAMURI, Fig. 4a), which has been shown to improve the stability of expressed functional RNAs in mammalian cells^48^. The SAMURI-encoding plasmid was transfected in HEK293T cells and, after 48-h incubation, the expression of target ribozyme was visualized by staining the Broccoli aptamer with DFHBI-1T (Fig. 4b).

**Extended Data Fig. 8:**
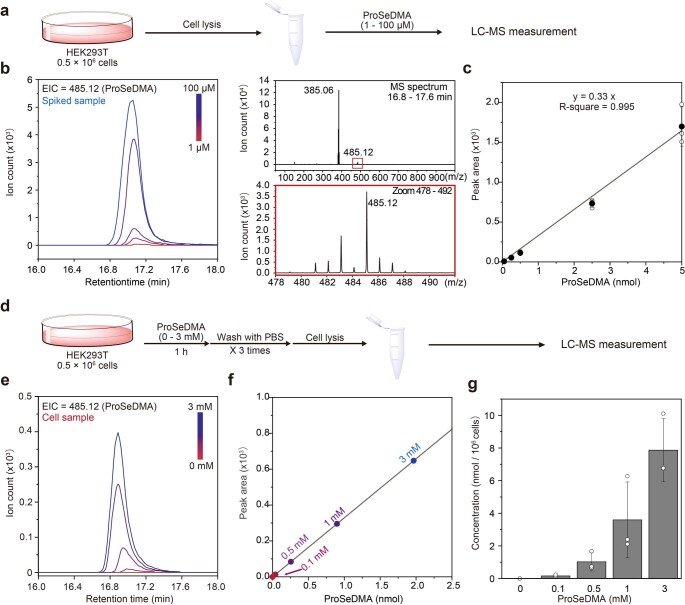
LC/MS analysis of ProSeDMA in HEK293T cell lysate. **a**. Schematic illustration for generation of a calibration curve with known concentrations of ProSeDMA spiked into HEK293T cell lysate. **b**. Extracted ion chromatogram (EIC) for ProSeDMA and MS spectrum at 17 min retention time in cell lysate. The fragmentation pattern was as detected by HR-ESI analysis of purified cofactor (shown in supporting information) **c**. Calibration curve of peak area versus injected amount of ProSeDMA; mean ± SD of three independent experiments. **d**. Schematic illustration of LC/MS analysis of ProSeDMA in the lysate of cells which were treated with various concentrations of ProSeDMA (0, 0.1, 0.5, 1, 3 mM in the medium). **e**. Detected ProSeDMA EIC chromatogram peak from cell samples. **f**. Detected peak area from the samples in **e** plotted on the calibration curve from **c**. **g**. ProSeDMA concentration in the cell lysate, calculated in nmol / 10^6^ cells. Individual data points and the mean ± SD of three independent experiments are shown. Source data

The cSAMURI-expressing cells were treated with increasing concentrations of ProSeDMA in growth medium (from 100 µM to 3 mM). Cell viability was not decreased under these conditions, suggesting that ProSeDMA is not strongly cytotoxic in this concentration range (Fig. 4c). We also estimated the cofactor uptake via LC-MS analysis of cleared cell lysate from untransfected cells (Extended Data Fig. 8). Using a calibration curve for the extracted ion count of ProSeDMA in cell lysate (generated by known spiked concentrations), we found ~3 nmol ProSeDMA per 10^6^ cells that were incubated in medium containing 1 mM ProSeDMA. These numbers are of the same order of magnitude as reported for bioorthogonal SAM analogues produced intracellularly from membrane-permeable amino acids and engineered SAM synthetases^18^. Therefore, ProSeDMA was sufficiently available for cSAMURI-expressing cells, from which total RNA was isolated and analysed. The intracellularly propargylated *cis*-active SAMURI was labelled with Cy5 azide, followed by visualization with in-gel fluorescence (Fig. 4d). The Cy5 channel image showed only one band, and its intensity increased with increasing concentrations of ProSeDMA in the medium. The same gel was stained by SYBR Gold to visualize total cellular RNA. The image shows the expected abundant cellular RNAs, such as transfer RNAs and ribosomal RNAs, and an additional band migrating approximately at the size of the 200-nt marker, which appeared only when the cells were transfected with the plasmid. This band stained with DFHBI-1T and represents the cyclized *cis*-active SAMURI-Broccoli-Tornado construct (cSAMURI). As expected, the ProSeDMA dose-dependent Cy5 signal (red) colocalized with the DFHBI-1T signal (green), indicated by the merged yellow colour in the multi-channel image of Cy5 and DFHBI-1T in Fig. 4d.

For comparison of the labelling efficiency, total RNA was also extracted from transfected cells that produced cSAMURI but were grown in the absence of ProSeDMA. The isolated RNA was then treated with the cofactor in vitro, followed by click labelling with Cy5. Using the normalized Cy5/DFHBI-1T ratio, we estimate that ~17% of cSAMURI was propargylated in the cell (Fig. 4e). Comparison to an in vitro-transcribed RNA, which was enzymatically processed and circularized, showed that the cSAMURI extracted from HEK293T cells retained its activity in the presence of total RNA. Overall, these results are encouraging for the future development of SAMURI as a self-labelling tag for any RNA of interest and for engineering *trans*-active SAMURI variants to target endogenous RNAs, similar to approaches recently shown for F30-MTR1 targeting *Escherichia coli* transfer RNA^31^, and circular ADAR-recruiting guide RNAs (cadRNAs)^49^ to target mRNAs.

## Conclusions

In summary, we have reported SAMURI as an alkyltransferase ribozyme in combination with ProSeDMA as a bioorthogonal and stabilized SAM analogue. The replacement of the terminal carboxylic acid by an amide improved the chemical stability of ProSeDMA compared to previously reported propargylic SAM and SeAM derivatives. The catalytic activity of SAMURI was demonstrated in vitro and in living cells. The successful intracellular application was possible due to the low toxicity of ProSeDMA and the high activity of SAMURI under physiological Mg^2+^ concentrations. Using atomic mutagenesis and MS, we fully characterized the RNA-catalysed reaction of SAMURI and found that the modification occurred at N3 of an unpaired adenosine between the binding arms. Although N3-alkylated adenosines are usually prone to depurination, we found that the enhanced electrophilicity at C2 provides a pathway for the favourable formation of a stable *N*-alkylated aminoimidazole nucleoside. Thus, SAMURI introduces the propargyl group site-specifically as a small clickable tag for RNA labelling. Moreover, SAMURI is permissive to ProSeDMA analogues, and can even use native SAM to synthesize m^3^A in RNA, which is a challenging chemical modification that cannot be prepared by solid-phase synthesis. This work expands the known repertoire of RNA-catalysed RNA modification reactions and suggests that appropriately designed cofactors and fine-tuned in vitro selection strategies are likely to create additional RNA-modifying ribozymes in the future. Desirable new ribozyme and cofactor combinations could then also enable strain-promoted click chemistry for applications in which the need for copper ions with the here-described propargyl groups is of any concern. Given the increasing importance of RNA technologies, we envision SAMURI to become a useful tool for RNA labelling and for studying the biology of damaged nucleobases in RNA.

## Methods

### RNA synthesis

RNA oligonucleotides were prepared by solid-phase synthesis using phosphoramidite chemistry with 2′-*O*-TOM-protection on controlled pore glass (CPG) solid supports^50^. RNA and DNA sequences are shown in Supplementary Tables 1 and 2. Modified phosphoramidites for atomic mutagenesis, c^1^A (ref. ^51^) and c^3^A (refs. ^52,53^), were prepared by following published procedures^54,55^. Other modified phosphoramidites were purchased. RNA oligonucleotides were deprotected with ammonia and methylamine (AMA), followed by 1 M tetrabutylammonium fluoride in THF, desalted and purified by denaturing PAGE. The quality of RNAs (purity and identity) was analysed by anion-exchange HPLC (Dionex DNAPac PA200, 2 × 250 mm, at 60 °C; solvent A was 25 mM Tris-HCl (pH 8.0) and 6 M urea; solvent B was 25 mM Tris-HCl (pH 8.0), 6 M urea and 0.5 M NaClO_4_; the gradient was linear, 0–40% solvent B, with a slope of 4% solvent B per column volume, flow rate 0.5 ml min^−1^, UV detection at 260 nm) and HR-ESI-MS (micrOTOF-Q III, negative-mode, direct injection). Measured and calculated masses are listed in Supplementary Table 3.

### Stability assay of ProSeDMA by HPLC analysis

1 mM ProSeDMA was mixed in a total volume of 100 μl of reaction buffer (50 mM HEPES, 120 mM KCl, 5 mM NaCl and 10 mM MgCl_2_, pH 6.0, 7.0 or 8.0) or with cell lysate generated from HEK293T cells with RIPA buffer (150 mM NaCl, 1.0% IGEPAL CA-630, 0.5% sodium deoxycholate, 0.1% SDS, 50 mM Tris, pH 7.5). The reaction mixtures were incubated at 37 °C, and 20 μl aliquots were taken from the samples to 40 μl of H_2_O + 0.1% trifluoroacetic acid (TFA) solution every 1 h. The collected samples were analysed by RP–HPLC (NUCLEOSIL 100-5 C18 column; 5 μm, 125 × 4 mm). The analysis was run with a linear gradient: B conc. 5–7% (0–15 min), 7–70% (15–30 min); solvent A was H_2_O + 0.1% TFA; solvent B was MeCN + 0.1% TFA; and flow rate was 0.7 ml min^−1^ at 30 °C with UV detection at 260 nm.

### In vitro selection

The initial library was prepared by in vitro transcription from double-stranded template DNA made from two synthetic DNA oligonucleotides (**D2** and **D4** with N40: A:C:G:T = 1:1:1:1) by overlap extension using Klenow fragment DNA polymerase. We used 500 pmol template DNA for in vitro transcription with T7 RNA polymerase in reaction buffer (40 mM Tris-HCl pH 8.0, 10 mM dithiothreitol (DTT), 4 mM NTP, 30 mM MgCl_2_ and 2 mM spermidine). For the first selection round, the 5 nmol RNA pool (with 10% 3′-fluorescently labelled RNA, prepared by sodium periodate oxidation labelling with Lucifer yellow carbohydrazide) was annealed in selection buffer (120 mM KCl, 5 mM NaCl and 50 mM HEPES, pH 7.5; 3 min at 95 °C, then 10 min at 25 °C). Cofactor **5** or **9** was added together with MgCl_2_ and the reaction filled with water to 1 ml, to give final concentrations of 5 μM RNA, 5 μM cofactor and 10 mM MgCl_2_. In further selection rounds, 1 nmol RNA was used in 200 µl total volume for the selection step. After incubation at 37 °C for 1 h, an excess amount of cofactor was removed by ethanol precipitation. The reacted RNA was labelled with biotin azide via CuAAC (100 μM RNA, 500 μM biotin azide, 1 mM CuBr and 2 mM Tris((1-benzyl-4-triazolyl)methyl)amine (TBTA) in 10 μl of H_2_O:DMSO:^*t*^BuOH (5:3:1)). The biotinylated RNAs were captured using either neutravidin- or streptavidin-coated magnetic beads (Dynabeads, Thermo Fisher Scientific, ~1 nmol RNA per mg of beads), washed according to the manufacturer’s instructions and eluted with formamide and amplified by PCR with reverse transcription (RT–PCR) with Ready-to-Go RT–PCR beads (Illustra). The second PCR was done with a primer containing the T7 promoter. In vitro transcription was performed (total volume of 100 μl), followed by PAGE purification to prepare the enriched RNA library for the next selection rounds. After eight rounds of selection, the library was cloned using the TOPO-TA cloning kit, and colonies were randomly picked up for sequencing.

The catalytic activity of ribozyme candidates was checked by a streptavidin gel shift assay on native PAGE (5 pmol RNA, 1 μg of streptavidin in 1× Tris-buffered saline (TBS) buffer) after 1 h incubation with cofactors **5** or **9**, respectively, followed by CuAAC with biotin azide. The candidate **Rz3** with the highest *cis*-activity was used for further experiments.

### Analysis of the propargylated RNA

For anion-exchange HPLC analysis, the substrate RNA was incubated with ProSeDMA in reaction buffer (10 μM substrate RNA, 10 μM SAMURI, 100 μM ProSeDMA and 10 mM MgCl_2_) at 37 °C for 1 h and analysed by anion-exchange HPLC on a Dionex DNAPac PA200 column (8 μm, 250 × 2 mm). The analysis was run with a linear gradient of B: conc. 0–48% (0–20 min). Solvent A was 25 mM Tris-HCl (pH 8.0) and 6 M urea; solvent B was 25 mM Tris-HCl (pH 8.0), 6 M urea and 0.5 M NaClO_4_. The flow rate was 0.5 ml min^−1^ at 60 °C with UV detection at 260 nm. For the digestion assay, 10 pmol unmodified and modified RNA were treated with 0.5 U of RNase T1 in 10 μl of reaction solution (50 mM Tris-HCl, pH 7.5) for 5 min at 37 °C or in 10 μl of alkaline hydrolysis solution (25 mM NaOH) for 3 min at 95 °C. For MALDI–TOF analysis, unmodified, modified and isolated RNAs were applied on a matrix (20 mg ml^−1^ 3-hydroxypicolinic acid and 10 mg ml^−1^ diammonium hydrogen citrate). After drying the matrix, mass data were collected by negative-mode ionization. For LC-MS analysis, the 500 pmol unmodified and modified RNA were digested by 6.0 U of bacterial alkaline phosphatase and 1.0 U of SVPD in reaction buffer (40 mM Tris-HCl, 20 mM MgCl_2_, pH 7.5). After extracting the digested nucleoside mixture with 100 μl of chloroform, the aqueous layer was concentrated by lyophilization, and the residue was dissolved in 70 μl of 5 mM NH_4_OAc and analysed by LC-MS, using an Synergi Fusion RP column (Phenomenex, 4 μm, 250 × 2 mm). The analysis was run with a gradient of 0–5% (0–15 min) and 5–72.5% (15–45 min) of solvent B. Solvent A was 10 mM NH_4_OAc (pH 5.3), solvent B was MeCN and the flow rate was 0.2 ml min^−1^ at 25 °C with UV detection at 260 nm and online MS in a microTOF-Q III system in positive-ion mode.

### Kinetic assays of SAMURI-catalysed *trans*-alkylation reactions

We mixed 10 pmol of Cy5-labelled RNA and 100 pmol of SAMURI in reaction buffer (50 mM HEPES, 120 mM KCl and 5 mM NaCl). After an annealing step (3 min at 95 °C and 10 min at 25 °C), 10 mM MgCl_2_ and 100 pmol of cofactor were added and the water was filled up until the total volume was 10 μl. The mixture was incubated at 37 °C and 1 μl aliquots were taken at desired time points and quenched by adding 4 μl of stop solution (80% formamide, 89 mM Tris-HCl, 89 mM boric acid and 50 mM EDTA). Each time-point sample was analysed by denaturing PAGE (20% polyacrylamide), and the band intensities were quantified by fluorescence imaging using a 695/55-nm emission filter. The yield versus time data were fitted to *Y* = *Y*_max_(1 − exp(−*k*_obs_*t*) using Origin (2019). All kinetic assays were carried out as three independent replicates. For determination of apparent *K*_m_ values, *k*_obs_ was determined with different concentrations of ProSeDMA and the *k*_obs_ versus concentration data were fitted using the Michaelis–Menten equation.

### Construction of *cis*-active SAMURI containing plasmids

The insert of *cis*-active SAMURI was prepared by overlap extension of amplicon from previously reported plasmid^31^ and synthetic DNA oligonucleotides (details are provided in Supplementary Fig. 1). The insert DNA was cloned between the NotI and SacII sites of pAV-U6 + 27-Tornado–Broccoli (Addgene plasmid no. 25709). The sequence of the insert and successful ligation into the plasmid were confirmed by Sanger sequencing.

### Cell culture and transfection

1 × 10^5^ HEK293T cells (ATCC) were cultured in 1 ml of 1× Dulbecco’s modified Eagle medium (Life Technologies) with 10% fetal bovine serum, 100 U ml^−1^ penicillin and 100 μg ml^−1^ streptomycin in a 12-well culture plate. Cells were plated for transfection using FuGENE HD (Promega), according to the manufacturer’s instructions, with 2 μg cSAMURI plasmid in OptiMEM I reduced serum medium (Gibco). RNA expression was checked by fluorescent microscopy (ZOE, Bio-Rad) with DFHBI-1T. After 48 h of incubation, total RNA was collected from the cultured cells by mixing with Trizol LS reagent (Invitrogen).

### Cell viability assay

HEK293T cells were cultured in 1 ml of 1× Dulbecco’s modified Eagle medium with 10% fetal bovine serum, 100 U ml^−1^ penicillin and 100 μg ml^−1^ streptomycin in a 24-well culture plate. After 24 h, the medium was replaced by 0.5 ml OptiMEM containing 0.1–3 mM ProSeDMA and 50 mM HEPES (pH 7.5) and the cells were incubated for 6 h. The cells were then extensively washed with PBS and mixed with 0.4% trypan blue solution (Thermo Fisher Scientific) in a 1:1 ratio. After 5 min of incubation at room temperature, total and stained cells were counted and cell viability was calculated as (1 − (number of stained cells/number of total cells)) × 100 (%).

### Analysis of cellular uptake of ProSeDMA by LC-MS

HEK293T cells were seeded in a 12-well culture plate and grown for 24 h. The medium was replaced by 1 ml of OptiMEM containing 0, 0.1, 0.5, 1 or 3 mM ProSeDMA, 5 μg ml^−1^ actD and 50 mM HEPES (pH 7.5) and incubated for 1 h. The incubated cells were washed three times with 1× PBS and suspended in 200 μl of 50% methanol + 0.1% formic acid. The suspended cells were completely disrupted by sonication. The resulting cell lysate was extracted with 200 μl of chloroform and the water phase was evaporated. After evaporation, the residue was dissolved in 100 μl of H_2_O containing 0.1% formic acid. After filtration, 50 μl of the sample was injected into an LC/MS system using an RP-column (NUCLEOSIL 100-5 C18 column, 5 μm, 125 × 4 mm). The analysis was run with a linear gradient of B: conc. 3% (0–15 min), 3–23% (15–30 min); solvent A was H_2_O + 0.1% formic acid, solvent B was MeCN + 0.1% formic acid and the flow rate was 0.2 ml min^−1^ at 25 °C with UV detection at 260 nm. The micrOTOF-Q III with an ESI ion source was operated in positive-ion mode (MS range, 50–2,500), with a capillary voltage of 4.5 kV, end plate offset of 500 V, nitrogen nebulizer pressure of 1.4 bar, dry gas flow of 9 l min^−1^ and dry temperature of 200 °C. Data were analysed with Data Analysis software DA 4.2 (Bruker Daltonics). For the calibration curve, multiple concentrations of ProSeDMA (0.05, 0.25, 0.5, 2.5 and 5 nmol) were spiked into cell lysate generated from cells that were not treated with ProSeDMA, and the peak areas of the ProSeDMA extracted ion chromatograms were plotted against the ProSeDMA amount injected. The slope was determined and used for calculation of the concentration of ProSeDMA in the lysate of cells that were fed with ProSeDMA in the medium.

### Intracellular activity assay of *cis*-active ribozyme

After transfection, cells were cultured for 48 h and then the medium was replaced by 1 ml of OptiMEM containing 0.1–3 mM ProSeDMA, 5 μg ml^−1^ actD (RNA transcription inhibitor) and 50 mM HEPES (pH 7.5). After 6 h of incubation, the cells were extensively washed and total RNAs were collected by Trizol LS reagent. The collected RNA was labelled by the CuAAC reaction (200 ng RNA, 500 μM CuBr, 1 mM TBTA and 1 mM Cy5 azide were incubated in H_2_O/DMSO/^*t*^BuOH = 5/3/1 solution at 37 °C for 1 h). Labelled RNAs were analysed by 5% denaturing PAGE, and the gel was imaged in the Cy5 channel before staining with DFHBI-1T and SYBR Gold. The band intensities were quantified and the intracellular activity was calculated from the Cy5/DFHBI-1T ratio, relative to the in vitro labelling efficiency of total RNA extracted from cells that were not treated with ProSeDMA. This control was prepared by labelling in the test tube (200 ng total RNA, 50 μM ProSeDMA and 10 mM MgCl_2_, pH 7.5 at 37 °C for 1 h).

### Reporting summary

Further information on research design is available in the Nature Portfolio Reporting Summary linked to this Article.

## Online content

Any methods, additional references, Nature Portfolio reporting summaries, source data, extended data, supplementary information, acknowledgements, peer review information; details of author contributions and competing interests; and statements of data and code availability are available at 10.1038/s41557-023-01320-z.

### Supplementary information

Supplementary Information Supplementary Tables 1–4, Fig. 1, methods with schemes 1–3, NMR spectra, HR-ESI-MS spectra and HPLC chromatograms.

Reporting Summary

### Source data

Source Data Fig. 2 Unprocessed full size gels for Fig. 2c,e.

Source Data Fig. 2 Statistical source data for Fig. 2e.

Source Data Fig. 3 Unprocessed full size gels for Fig. 3a,b,d,f.

Source Data Fig. 3 Source data for plots in Fig. 3d,e,f,g.

Source Data Fig. 4 Unprocessed full size gels for Fig. 4d.

Source Data Fig. 4 Statistical source data for Fig. 4c,e.

Source Data Extended Data Fig. 1 Unprocessed gels for Extended Data Fig. 1b,c.

Source Data Extended Data Fig. 3 Unprocessed gels for Extended Data Fig. 3.

Source Data Extended Data Fig. 4 Unprocessed gels for Extended Data Fig. 4.

Source Data Extended Data Fig. 8 Source data for plots in Extended Data Fig 8.

## Data Availability

The data generated and analysed during this study are included in this published Article, in the Supplementary Information and in the source data files. Source data are provided with this paper.
