# Supplementary material for: A SAM analogue-utilizing ribozyme for site-specific RNA alkylation in living cells
Source: Nat Chem. 2023 Sep 4;15(11):1523–31. doi: 10.1038/s41557-023-01320-z (PMC10624628; doi:10.1038/s41557-023-01320-z)
Supplement: Supplementary file 1 — Supplementary Tables 1–4, Fig. 1, methods with schemes 1–3, NMR spectra, HR-ESI-MS spectra and HPLC chromatograms. [file 41557_2023_1320_MOESM1_ESM.pdf]

nature chemistry

Article https://doi.org/10.1038/s41557-023-01320-z

A SAM analogue-utilizing ribozyme for site-specific RNA alkylation in living cells

In the format provided by the authors and unedited

Table of contents page

Supplementary Tables and Figures

Supplementary Table 1: DNA oligonucleotides used as primers and templates 2

Supplementary Table 2: RNA oligonucleotides prepared by solid-phase synthesis 3

Supplementary Table 3: MALDI/ESI-MS data for RNA substrates and alkylated products 3

Supplementary Table 4: Ribozyme sequence constructs for cellular experiments 4

Supplementary Figure 1: Schematic presentation of plasmid preparation 4

General materials and synthetic procedures 5

Synthesis of SeDMA analogues 5

Synthesis of reference materials 14

NMR spectra 15

HR-ESI mass spectra 35

HPLC chromatograms 37

Supplementary References 38

Supplementary Tables and Figures

Supplementary Table 1. DNA oligonucleotides: Primers and transcription templates

No.	Description	5'-sequence-3'
D1	T7 promotor	CTGTAATACGACTCACTATA
D2	DNA pool	GGTAAGGTGGACATACTG-N40-GCCTTCAAGGATGGTAGGCTGG
D3	Selection primer (forward for 1st PCR)	CTTCAACCAGCCTACCATCC
D4	Selection primer (forward for 2nd PCR)	CTGTAATACGACTCACTATAGGACATACTGAGCCTTCAACCAGCCTACCATCC
D5	Selection primer (reverse)	GGTAAGGTGGACATACTG
D6	Cloning primer (reverse)	TAAATAAAATAACTGTAATACGACTCACTATAGGACATACTGAGC
D7	Template (Rz3_C40U)	GGACATACTGCAGCAACGCTTCCGTCACCGGAGCGCCCTGAGCCGTCTGAGCCTTCAACCTATAGTGAGTC GTATTACAG
D8	Template (Rz3_C45A)	GGACATACTGCAGCAACGCTTCCGTCACCGGAGCGCCCTGAGCCTTCTGGGCCTTCAACCTATAGTGAGTC GTATTACAG
D9	Template (Rz3_Loop1)	GGACATACTGCAGCGGCGCTGACCGAAGTCAGCGCCCTGAGCCGTCTGGGCCTTCAACCTATAGTGAGTCG TATTACAG
D10	Template (Rz3_Loop2)	GGACATACTGCAGCGGACCGAAGTCCCTGAGCCGTCTGGGCCTTCAACCTATAGTGAGTCGTATTACAG
D11	Template (Rz3_Loop3)	GGACATACTGCAGGGACCGAAGTCCCTGAGCCGTCTGGGCCTTCAACCTATAGTGAGTCGTATTACAG
D12	Template (Rz3_Loop4)	GGACATACTGCAGGCCGAAGCCTGAGCCGTCTGGGCCTTCAACCTATAGTGAGTCGTATTACAG
D13	Template (Rz3_G75D)	GGACATACTGCAGAACGCTTCCGTCACCGGAGCGCCCTGAGCCGTCTGGGCCTTCAACCTATAGTGAGTCG TATTACAG
D14	Template (Rz3_U82A)	GGACATTCTGCAGCAACGCTTCCGTCACCGGAGCGCCCTGAGCCGTCTGGGCCTTCAACCTATAGTGAGTC GTATTACAG
D15	Template (Rz3')	GGACATACTGCAGCGGACCGAAGTCCCTGAGCCGTCTGAGCCTTCAACCTATAGTGAGTCGTATTACAG
D16	Template (Rz3")	GGACATACTGACTGCAGCGGACCGAAGTCCCTGAGCCGTGCCTTCAACCTATAGTGAGTCGTATTACAG
D17	Template (SAMURI)	GGACATACTGACTGCAGCGGACCGAAGTCCCTGAGCCATGCCTTCAACCTATAGTGAGTCGTATTACAG
D18	Template (SAMURI_short binding_arm)	CATACTGACTGCAGCGGACCGAAGTCCCTGAGCCATGCCTTCCTATAGTGAGTCGTATTACAG
D19	SAMURI_Tornado (Insert fraction 1)	CATGCCGAGTGCGGCCGCTTGCCATGTGTATGTGGGAGACGGT
D20	SAMURI_Tornado (Insert fraction 2)	TGAGCCTTCAAGGATGGTAGGCTGGTT
D21	SAMURI_Tornado (Insert fraction 3)	AACCAGCCTACCATCCTTGAAGGCTCAGATGGCTCAGGGACTTCGATCCGCTGCAGTATGTCCAT
D22	SAMURI_Tornado (Insert fraction 4)	TCCACGCCGACCGCGGCCACTTGCCATGAATGATCCATGGACATACTGCAGCGGATCGAAGTCCCTG
D23	Template（SAMURI GAA）	GGACATACTGACTGCAGCGGACCGAAGTCCCTGAGCCATACCTTCAACCTATAGTGAGTCGTATTACAG
D24	Template (SAMURI AAG)	GGACATACTAACTGCAGCGGACCGAAGTCCCTGAGCCATGCCTTCAACCTATAGTGAGTCGTATTACAG
D25	Template (substrate RNA GAA)	TTGAAGGCTTAGTATGTCCTATAGTGAGTCGTATTACAG
D26	Template (substrate RNA AAG)	TTGAAGGTTCAGTATGTCCTATAGTGAGTCGTATTACAG
D27	Primer (extension stop assay)	Alk-CAAGGATGGTAGGCTGGT

Supplementary Table 2. RNA oligonucleotides: substrate RNA

No.	Description	5'-sequence-3'
R1	Substrate RNA for SAMURI	ACAUACUGAGCCUUCAA-NH2
R2	Substrate RNA for SAMURI (LCMS)	ACAUACUGAGCCUUCAA
R3	Substrate RNA for SAMURI (LCMS2)	CUACUGAGCCUU
R4	Substrate RNA for SAMURI (with c1A)	ACAUACUG(c1A)GCCUUCAA-NH2
R5	Substrate RNA for SAMURI (with c3A)	ACAUACUG(c3A)GCCUUCAA-NH2
R6	Substrate RNA for SAMURI (with c7A)	ACAUACUG(c7A)GCCUUCAA-NH2
R7	Substrate RNA for SAMURI (with dA)	ACAUACUG(dA)GCCUUCAA-NH2

Supplementary Table 3. ESI-MS analysis of synthetic and SAMURI-alkylated RNAs

No.	Description	Chemical formula	Mass calculated	Mass found
R1	Substrate RNA for SAMURI	$C_{167}H_{215}N_{64}O_{118}P_{17}$	5530.83 Da	5530.86 Da
R2	Substrate RNA for SAMURI (LCMS)	$C_{161}H_{201}N_{63}O_{115}P_{16}$	5351.76 Da	5351.76 Da
R3	Substrate RNA for SAMURI (LCMS2)	$C_{112}H_{141}N_{40}O_{84}P_{11}$	3730.51 Da	3730.50 Da
R4	Substrate RNA for SAMURI (with c1A)	$C_{168}H_{216}N_{63}O_{118}P_{17}$	5529.84 Da	5529.85 Da
R5	Substrate RNA for SAMURI (with c3A)	$C_{168}H_{216}N_{63}O_{118}P_{17}$	5529.84 Da	5529.86 Da
R6	Substrate RNA for SAMURI (with c7A)	$C_{168}H_{216}N_{63}O_{118}P_{17}$	5529.84 Da	5529.88 Da
R7	Substrate RNA for SAMURI (with dA)	$C_{167}H_{215}N_{64}O_{117}P_{17}$	5514.84 Da	5514.86 Da
R1Cy5	Substrate RNA for SAMURI with Cy5	$C_{199}H_{251}N_{66}O_{125}P_{17}S_{2}$	6155.03 Da	6155.04 Da
R1Cy5-Me	R1 modified by MeSeDMA	$C_{200}H_{253}N_{66}O_{125}P_{17}S_{2}$	6169.05 Da	6169.03 Da
R1Cy5-All	R1 modified by AllSeDMA	$C_{202}H_{255}N_{66}O_{125}P_{17}S_{2}$	6195.06 Da	6195.00 Da
R1Cy5-Pro*	R1 modified by ProSeDMA	$C_{202}H_{253}N_{66}O_{125}P_{17}S_{2}$	6193.05 Da	6193.06 Da
R1Cy5-Pro	R1 modified by ProSeDMA (amino imidazole)	$C_{201}H_{255}N_{66}O_{125}P_{17}S_{2}$	6183.06 Da	6183.08 Da
R4Cy5-Pro	R4 modified by ProSeDMA	$C_{203}H_{254}N_{65}O_{125}P_{17}S_{2}$	6192.05 Da	6192.04 Da
R6Cy5-Pro	R6 modified by ProSeDMA	$C_{203}H_{254}N_{65}O_{125}P_{17}S_{2}$	6192.05 Da	6192.06 Da

*The mass was measured by MALDI-TOF before PAGE purification.

Supplementary Table 4. Sequences for intracellular application

No.	Description	5'-sequence-3'
P1	F30-Broccoli-MTR1cis (Broccoli green, substrate red, MTR1 blue, binding arms underlined)	GGGUUGCCAUGUGUAUGUGGGAGACGGUCGGGUCCAGAUAUUCGUAUCUGUCGAGUAGAGUGUGGGCUCCCAUAUACUCUGAUGAUCCUCGAGGACAUACUG**A**GCCUUCAACCAGCCUACCAUCCUUGAAGGCUGACCGACCCCCCGAGUUCGCUCGGGGACAACUAGACAUACAGUAUGUCCAUGGAUCAUUCAUGGCAAGC
P2	F30-Broccoli-SAMURIcis_Tornado (Broccoli green, substrate red, SAMURI blue, binding arms underlined, Tornado orange)	GGCCGCACUCGCCGGUCCCAAGCCCGGAUAAAAUGGGAGGGGGCGGGAAACCGCCUAACCAUGCCGAGUGCGGCCGCUUGCCAUGUGUAUGUGGGAGACGGUCGGGUCCAGAUAUUCGUAUCUGUCGAGUAGAGUGUGGGCUCCCAUAUACUCUGAUGAUCCUCGAGGACAU**A**CUGAGCCUUCAACCAGCCUACCAUCCUUGAAGGCUCAGAUGGCUCAGGGACUUCGAUCCGCUGCAGUAUGUCCAUGGAUCAUUCAUGGCAAGUGGCCGCGGUCGGCGUGGACUGUAGAACACUGCCAAUGCCGGUCCCAAGCCCGGAUAAAAGUGGAGGGUACAGUCCACGC

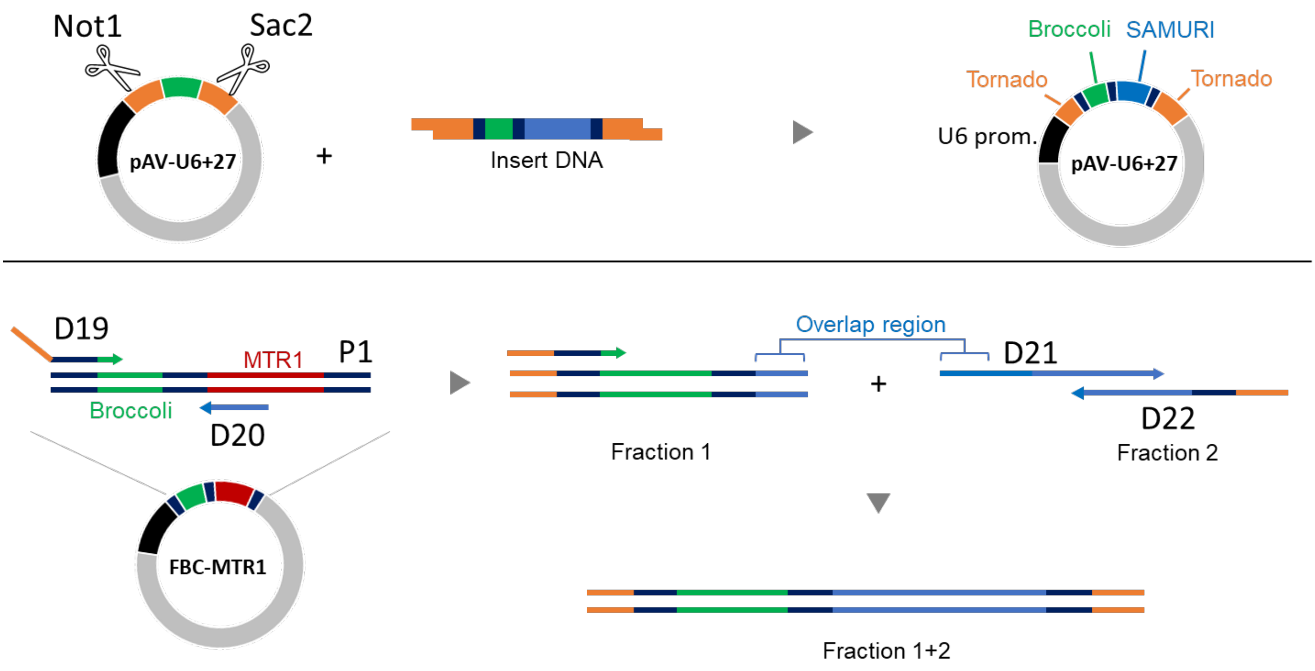

Supplementary Fig. 1 Schematic illustration of plasmid preparation. F30-Broccoli scaffold was taken from the previously reported FBC-MTR1 plasmid with Tornado conjugated primer D19. SAMURI region was consisted by overlap PCR and these two fractions were also connected by overlap PCR. The insert and pAV-U6+27-Tornado-Broccoli (Addgene, plasmid no. 25709) were treated under double digestion condition (Not1 and Sac2) and cut fractions were purified by 0.8% agarose gel (75 V, 45 min). The sequence of the insert and successful ligation into the plasmid was confirmed by Sanger sequencing.

General materials and methods

All standard chemicals and solvents were purchased from commercial suppliers. Dry solvents (dichloromethane, THF, DMF, acetonitrile) were obtained from a solvent purification system (SPS). Water for in vitro experiment was obtained from a Sartorius Arium® pro ultrapure water system. Silica gel plates coated with fluorescent indicator were used for thin layer chromatography (TLC) and the plates were visualized with UV light. Silica gel (Kieselgel 60, Merck, 0.063 – 0.200 mm) was used for column chromatography. NMR spectra were measured on a Bruker Avance III HD 400 spectrometer at 400 MHz. Spectral assignments were verified by 2D NMR experiments. High resolution ESI mass spectra were measured on a Bruker micrOTOF-Q III spectrometer. Monoisotopic masses for oligonucleotides were obtained by charge deconvolution of the raw spectra. Unmodified DNA oligonucleotides were purchased from Microsynth and purified by PAGE. Dynabeads streptavidin T1 and speedbead neutravidin coated-magnetic particles were purchased from Thermo Fisher scientific and GE-healthcare respectively. Fluorophores were purchased from lumiprobe. All other chemicals were purchased from Sigma-Aldrich or ABCR and used without further purification. HPLC grade solvents were purchased from VWR. Fluorescent imaging of the kinetic and activity assay gels was taken using a BioRad Chemidoc gel-documentation device.

Synthesis of ProSeDMA analogues

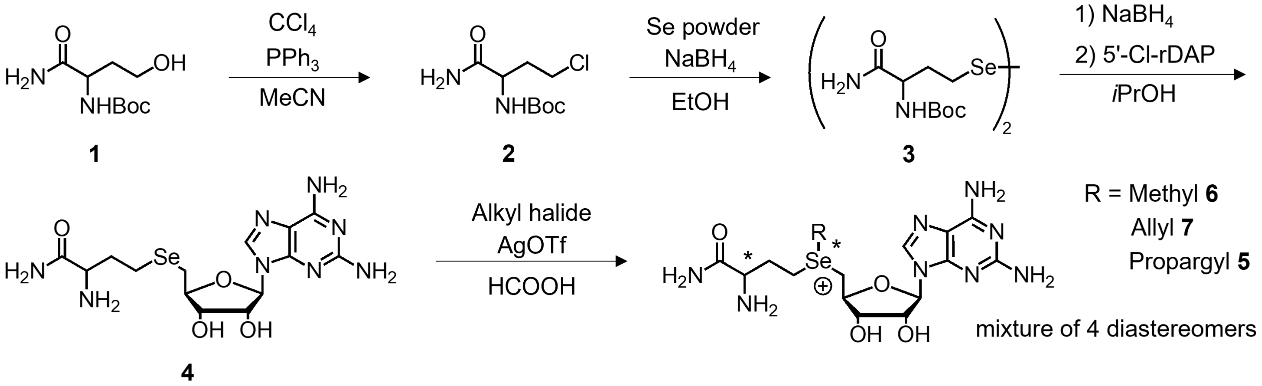

Supplementary Scheme 1. Synthesis of ProSeDMA NH2

N$^{\alpha}$-Boc-2-Amino-4-chlorobutanamide (2)

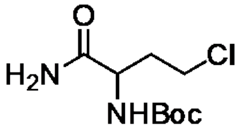

To a solution of compound **1** (530 mg, 2.4 mmol, obtained in two steps form racemic α-amino-γ-butyrolactone[1]) in dry acetonitrile (24 mL), CCl_4 (284 µL, 2.9 mmol) and triphenylphosphine (957mg, 3.7 mmol) were added under stirring at room temperature. The reaction was continued for 1 h with

heating under reflux. After quenching with MeOH, solvent was removed by evaporation and the residue was extracted with EtOAc / saturated $NaHCO_3$ aq., water, brine. The organic layer was dried by Na_2SO_4 and evaporated. The crude product was purified by column chromatography on SiO_2 with DCM / *i*PrOH = 95 / 5. Yield: 302 mg of **2** as white solid (53 %).

^{1}H NMR (400 MHz, DMSO-d6) δ 7.30 (s, 1H), 7.04 (s, 1H), 6.98 (d, *J* = 8.3 Hz, 1H), 3.99 (dt, *J* = 8.3, 7.3 Hz, 1H), 3.60 (t, J = 7.3 Hz, 2H), 2.05 – 1.93 (m, 2H), 1.39 (s, 9H). **^{13}C NMR** (100 MHz, DMSO) δ 173.92, 155.86, 78.60, 52.29, 42.37, 35.09, 28.63.

ESI-MS (*m/z*): $[M+Na]^+$ calcd for $C_9H_{17}ClN_2NaO_3$, 259.08199; found 259.08252.

N$^\alpha$-Boc-selenohomocystineamide (3)

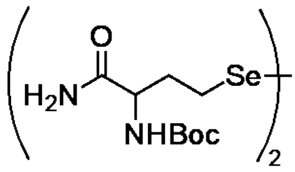

To a suspension of selenium powder (804 mg, 10.2 mmol) in ethanol (30 mL), sodium borohydride (257 mg, 6.8 mmol) was added. After stirring for 30 min at room temperature, compound **2** (800 mg, 3.4 mmol) was added in the solution and the reaction was allowed to proceed next 15 h. The solvent was switched to EtOAc and extracted by saturated $NaHCO_3$ aq., water and brine. The organic layer was dried over Na_2SO_4 and evaporated. The crude product was purified by column chromatography on SiO_2 with DCM / MeOH = 95 / 5. Yield: 583 mg of **3** as slightly yellow foam (61 %).

^{1}H NMR (400 MHz, DMSO-d6) δ 7.28 (s, 1H), 7.03 (s, 1H), 6.88 (d, *J* = 8.3 Hz, 1H), 3.93 (dt, *J* = 8.3, 7.3 Hz, 1H), 2.88 (t, *J* = 7.7 Hz, 2H), 1.96 (dt, *J* = 7.7, 7.3 Hz, 2H), 1.38 (s, 9H). **^{13}C NMR** (100 MHz, DMSO) δ 174.03, 155.77, 78.56, 54.49, 34.14, 28.66, 25.97.

ESI-MS (*m/z*): $[M+Na]^+$ calcd for $C_{18}H_{34}N_4NaO_6Se_2$, 585.07010; found 585.07142.

Se-2,6-Diaminopurineribosyl-selenohomocysteineamide (4)

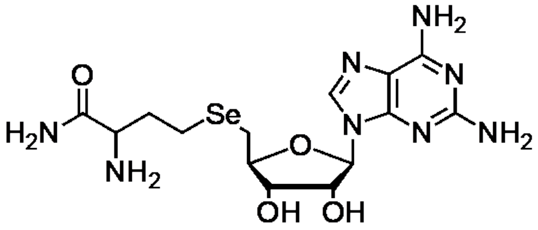

Compound **3** (10 mg, 18 µmol) was activated by sodium borohydride (7.5 mg, 200 µmol) in *i*PrOH (1 mL). After 15 min, 9-(5'-chloro-5'-deoxy-β-D-ribofuranosyl)-2,6-diamiopurine (= 5'-chloro-2-aminoadenosine) (9 mg, 30 µmol) was added and the reaction was proceeded for 2 h with heating

under reflux. The reaction mixture was diluted by DCM and extracted with water. The aqueous phase was dried by evaporation and the residue was dissolved in formic acid (1 mL). After overnight Boc-deprotection, the solvent was removed and the crude mixture was purified by C18 reverse phase HPLC (2 mL/min, NUCLEODUR® C18 Pyramid VP 250/21 column, linear gradient B conc. 5% - 7% (0 min to 15 min), 7% - 70% (15min to 30 min); solvent A was H_2O + 0.1% TFA; solvent B was MeCN + 0.1% TFA; flow rate was 2 ml/min at 30 °C with UV detection at 260 nm.) Fractions containing the desired material were combined and the solvent was removed by lyophilization. Yield: 17 µmol (determined by UV absorbance at 279 nm, $\varepsilon_{279\ nm}$ = 9,894 $Lmol^{-1}cm^{-1}$) of **4** as white foam (57 %).

^{1}H NMR (400 MHz, D_2O) δ 7.97 (s, 1H), 5.74 (d, *J* = 4.8 Hz, 1H), 4.66 (m, 1H) 4.24 (dd, *J* = 5.0 Hz, 4.8 Hz, 1H), 4.16 (m, 1H), 3.94 (m, 1H), 2.96 – 2.82 (m, 2H), 2.53 (m, 2H), 2.13 – 2.03 (m, 2H). **^{13}C NMR** (100 MHz, D_2O) δ 171.26, 152.05, 151.44, 149.53, 140.29, 111.25, 87.66, 83.65, 73.21, 72.85, 52.82, 31.68, 25.59, 17.99

ESI-MS (*m/z*): $[M+H]^+$ calcd for $C_{14}H_{23}N_8O_4Se$, 447.10020; found 447.10173.

Propargylic *Se*-2,6-diaminopurineribosyl-selenomethionineamide (5)

***Se*-2,6-diaminopurineribosyl-selenomethionineamide (6)**

Allylic *Se*-2,6-diaminopurineribosylselenomethionineamide (7)

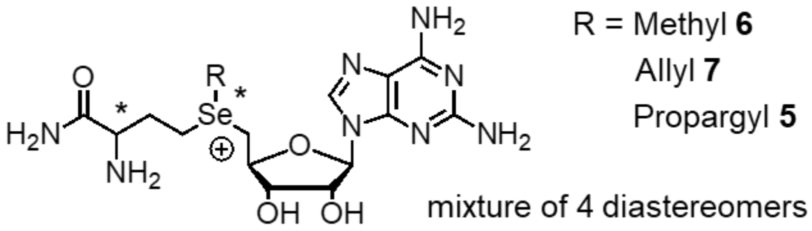

Compound **4** (4.5 mg, 10 µmol) was dissolved in formic acid (1 mL) and the solution was cooled in an ice bath. Alkyl halide (**5**: propargyl bromide, 80% in toluene, ca. 9.2 mol/L, 109 µL, 1 mmol, **6**: methyl iodide, 62 µL, 1 mmol, **7**: allyl bromide, 86 µL, 1 mmol) and silver triflate (13 mg, 50 µmol) were added and the reaction mixture was stirred for 48 h. The reaction was quenched by MeOH (500 µL) and the solvent was removed by evaporation. The residue was suspended in H_2O and insoluble Ag salt was filtered off. The crude mixture was purified by C18 reverse phase HPLC (2 mL/min, NUCLEODUR® C18 Pyramid VP 250/21 column, linear gradient B conc. 5% - 7% (0 min to 15 min), 7% - 70% (15min to 30 min); solvent A was H_2O + 0.1% TFA; solvent B was MeCN + 0.1% TFA; flow rate was 2 ml/min at 30 °C with UV detection at 260 nm.) Fractions containing the desired material were combined and the solvent was removed by lyophilization.

Yield: 0.6 µmol (determined by UV absorbance at 279 nm, $\varepsilon_{279\ nm}$ = 9,894 $Lmol^{-1}cm^{-1}$) of **5** as white foam (6 %). **^{1}H NMR** (400 MHz, D_2O) δ 8.02 (m, 1H), 5.97 – 5.70 (m, 1H), 4.63 – 4.52 (m, 1H), 4.46

– 4.38 (m, 1H), 4.30 (m, 1H), 4.13 – 3.98 (m, 2H), 3.86 (m, 2H), 3.36 (m, 2H), 3.18 – 2.96 (m, 1H), 2.57 – 2.25 (m, 2H).**^{13}C NMR** (100 MHz, D_2O) δ 169.91, 152.66, 151.45, 150.16, 140.94, 114.83, 89.33, 80.63, 79.13, 73.42, 72.98, 70.57, 67.40, 41.20, 33.13, 27.80, 25.90.
ESI-MS (*m/z*): $[M]^+$ calcd for $C_{17}H_{25}N_8O_4Se$, 485.11585; found 485.11789

2 μmol (determined by UV absorbance at 279 nm, $\varepsilon_{279\ nm}$ = 9,894 $Lmol^{-1}cm^{-1}$) of **6** as white foam (20 %). **^{1}H NMR** (400 MHz, D_2O) δ 7.97 – 7.96 (m, 1H), 5.84 – 5.83 (m, 1H), 4.50 – 4.45 (m, 1H), 4.41 – 4.33 (m, 1H), 3.99 – 3.95 (m, J = 6.4, 3.7 Hz, 1H), 3.84 – 3.71 (m, 2H), 3.38 – 3.19 (m, 2H), 2.75 – 2.70 (m, J = 11.6 Hz, 3H), 2.32 – 2.16 (m, 2H). **^{13}C NMR** (100 MHz, D_2O) δ 169.90, 152.12, 151.39, 149.73, 140.95, 111.86, 89.32, 78.81, 72.94, 72.74, 51.75, 41.84, 33.89, 26.15, 19.63.
ESI-MS (*m/z*): $[M]^+$ calcd for $C_{15}H_{25}N_8O_4Se$, 461.11585; found 461.11594.

0.4 μmol (determined by UV absorbance at 279 nm, $\varepsilon_{279\ nm}$ = 9,894 $Lmol^{-1}cm^{-1}$) of **7** as white foam (4 %). **^{1}H NMR** (400 MHz, D_2O) δ 7.97 (s, 1H), 5.84 (d, J = 3.5 Hz, 1H), 5.81 – 5.70 (m, 1H), 5.51 – 5.39 (m, 2H), 4.53 – 4.47 (m, 2H), 4.40 – 4.34 (m, 2H), 4.01 – 3.94 (m, 3H), 3.77 – 3.65 (m, 2H), 3.24 – 3.15 (m, 2H), 2.30 – 2.15 (m, 2H). **^{13}C NMR** (100 MHz, D_2O) δ 169.92, 152.12, 151.38, 149.77, 141.07, 127.66, 124.28, 111.91, 89.46, 78.54, 72.96, 72.67, 51.79, 41.01, 39.69, 31.92, 26.26.
ESI-MS (*m/z*): $[M]^+$ calcd for $C_{17}H_{27}N_8O_4Se$, 487.13150; found 487.13096.

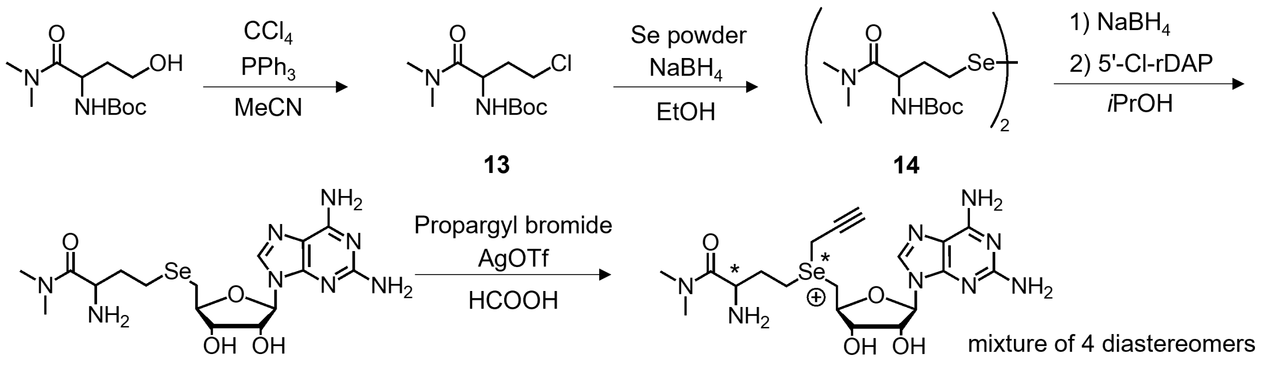

Supplementary Scheme 2. Synthesis of ProSeDMA NMe₂

N$^\alpha$-Boc-2-Amino-4-chlorobutan-*N,N*-dimethylamide (13)

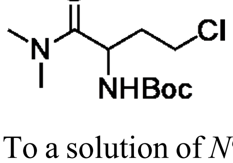

To a solution of *N$^\alpha$*-Boc-2-amino-4-hydroxybutan-dimethylamide (500 mg, 2.3 mmol, obtained in two steps form racemic α-amino-γ-butyrolactone[1]) in dry acetonitrile (23 mL), CCl_4 (280 μL, 2.8 mmol)

and triphenylphosphine (905mg, 3.5 mmol) were added under stirring at room temperature. The reaction was continued for 1 h with heating under reflux. After quenching with MeOH, solvent was removed by evaporation and the residue was extracted with EtOAc / saturated $NaHCO_3$ aq., water, and brine. The organic layer was dried over Na_2SO_4 and evaporated. The crude product was purified by column chromatography on SiO_2 with DCM / EtOAc = 1 / 1. Yield: 600 mg of **13** as white solid (98%).
^{1}H NMR (400 MHz, DMSO-d6) δ 7.10 (d, J = 8.4 Hz, 1H), 4.55 (dt, J = 8.4, 5.2 Hz, 1H), 3.63 (t, J = 6.9 Hz, 2H), 3.03 (s, 3H), 2.83 (s, 3H), 1.94 (dt, J = 6.9, 5.2 Hz, 2H), 1.37 (s, 9H). **^{13}C NMR** (100 MHz, DMSO) δ 171.34, 155.87, 78.60, 48.30, 42.52, 40.59, 36.77, 35.74, 34.60, 28.61.
ESI-MS (*m/z*): $[M+Na]^+$ calcd for $C_{11}H_{21}ClN_2NaO_3$, 287.11329; found 287.11394.

***N^α*-Boc-selenohomocystine-*N*,*N*-dimethylamide (14)**

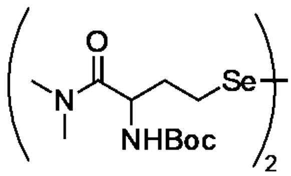

To a suspension of selenium powder (447 mg, 5.7 mmol) in ethanol (20 mL), sodium borohydride (144 mg, 3.8 mmol) was added. After stirring for 30 min at room temperature, compound **13** (500 mg, 1.9 mmol) was added in the solution and the reaction was allowed to proceed next 15 h. The solvent was switch to EtOAc and extracted by saturated $NaHCO_3$ aq., water, brine. The organic layer was dried by Na_2SO_4 and evaporated. The crude product was purified by column chromatography on SiO_2 with DCM / EtOAc = 2 / 3. Yield: 310 mg of **14** as slightly yellow foam (53 %).
^{1}H NMR (400 MHz, DMSO-d6) δ 7.02 (d, J = 6.1 Hz, 1H), 4.48 (td, J = 8.5, 6.1 Hz, 1H), 3.02 (s, 3H), 2.90 (t, J = 8.0 Hz, 2H), 2.83 (s, 3H), 1.97 – 1.82 (m, 2H), 1.37 (s, 9H). **^{13}C NMR** (100 MHz, DMSO) δ 171.52, 155.85, 78.54, 50.38, 36.88, 35.73, 33.12, 28.65, 25.89.
ESI-MS (*m/z*): $[M+Na]^+$ calcd for $C_{22}H_{42}N_4NaO_6Se_2$ 641.13270; found 641.13527.

***Se*-2,6-Diaminopurineribosylselenohomocysteine-*N*,*N*-dimethylamide (15)**

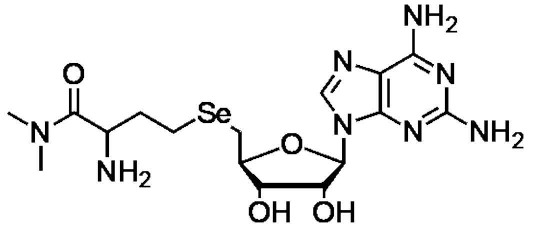

Compound **14** (10 mg, 16 μmol) was activated by sodium borohydride (6.1 mg, 160 μmol) in *i*PrOH (1 mL). After 15 min, 5'-chloro-2-aminoadenosine (9 mg, 30 μmol) was added and the reaction was

proceeded for 2 h with heating under reflux. The reaction mixture was diluted by DCM and extracted with water. The aqueous phase was dried by evaporation and the residue was dissolved in formic acid (1 mL). After overnight Boc-deprotection, the solvent was removed and the crude mixture was purified by C18 reverse phase HPLC (2 mL/min, NUCLEODUR® C18 Pyramid VP 250/21 column, linear gradient B conc. 5% - 7% (0 min to 15 min), 7% - 70% (15min to 30 min); solvent A was H_2O + 0.1% TFA; solvent B was MeCN + 0.1% TFA; flow rate was 2 ml/min at 30 °C with UV detection at 260 nm.) Fractions containing the desired material were combined and removal of solvent by lyophilization. Yield: 3.8 µmol (determined by UV absorbance at 279 nm, $\varepsilon_{279\ nm} = 9{,}894\ Lmol^{-1}cm^{-1}$) of **15** as white foam (24 %).

^{1}H NMR (400 MHz, D_2O) δ 8.04 (s, 1H), 5.81 (d, J = 4.9 Hz, 1H), 4.44 – 4.40 (m, 1H), 4.30 – 4.27 (m, 1H), 4.22 – 4.17 (m, 1H), 3.02 – 2.87 (m, 5H), 2.81 (s, 3H), 2.60 – 2.48 (m, 2H), 2.12 – 1.97 (m, 2H). **^{13}C NMR** (100 MHz, D_2O) δ 168.66, 152.27, 151.63, 149.81, 140.38, 111.54, 87.59, 83.88, 72.99, 72.85, 50.37, 36.80, 35.68, 30.81, 25.58, 18.03.

ESI-MS (*m/z*): $[M+H]^+$ calcd for $C_{16}H_{27}N_8O_4Se$, 475.13150; found 475.13268.

Propargylic *Se*-2,6-diaminopurineribosylselenomethionine-N,N-dimethylamide (8)

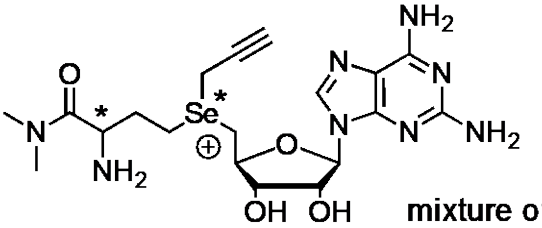

mixture of 4 diastereomer

Compound **15** (4.7 mg, 10 µmol) was dissolved in formic acid (1 mL) and the solution was cooled on ice bath. Propargyl bromide,80% in toluene, ca. 9.2 mol/L, 109 µL, 1 mmol and silver triflate (13 mg, 50 µmol) were added and the reaction mixture was stirred next 48 h. The reaction was quench by MeOH (500 µL) and the solvent was removed by evaporation. The residue was suspended in H_2O and insoluble Ag salt was filtered off. The crude mixture was purified C18 reverse phase HPLC (2 mL/min, NUCLEODUR® C18 Pyramid VP 250/21 column, linear gradient B conc. 5% - 7% (0 min to 15 min), 7% - 70% (15min to 30 min); solvent A was H_2O + 0.1% TFA; solvent B was MeCN + 0.1% TFA; flow rate was 2 ml/min at 30 °C with UV detection at 260 nm.) Fractions containing the desired material were combined and removal of solvent by lyophilization. Yield: 1 µmol (determined by UV absorbance at 279 nm, $\varepsilon_{279\ nm} = 9{,}894\ Lmol^{-1}cm^{-1}$) of **8** as white foam (10 %).

^{1}H NMR (400 MHz, D_2O) δ 7.99 – 7.98 (m, 1H), 5.86 – 5.85 (m, 1H), 4.60 – 4.55 (m, 1H), 4.55 – 4.50 (m, 1H), 4.43 – 4.38 (m, 1H), 4.16 – 4.00 (m, 2H), 3.93 – 3.76 (m, 2H), 3.40 – 3.19 (m, 2H), 3.17 – 3.07 (m, 1H), 2.98 – 2.75 (m, 6H), 2.32 – 2.16 (m, 2H).

^{13}**C NMR** (100 MHz, D_2O) δ 167.30, 154.06, 153.24, 152.23, 140.80, 114.81, 89.47, 79.02, 73.34, 72.90, 65.49, 49.25, 41.45, 36.90, 32.78, 26.16, 25.30.

ESI-MS (*m/z*): $[M]^+$ calcd for $C_{19}H_{29}N_8O_4Se$, 513.14715; found 513.14589

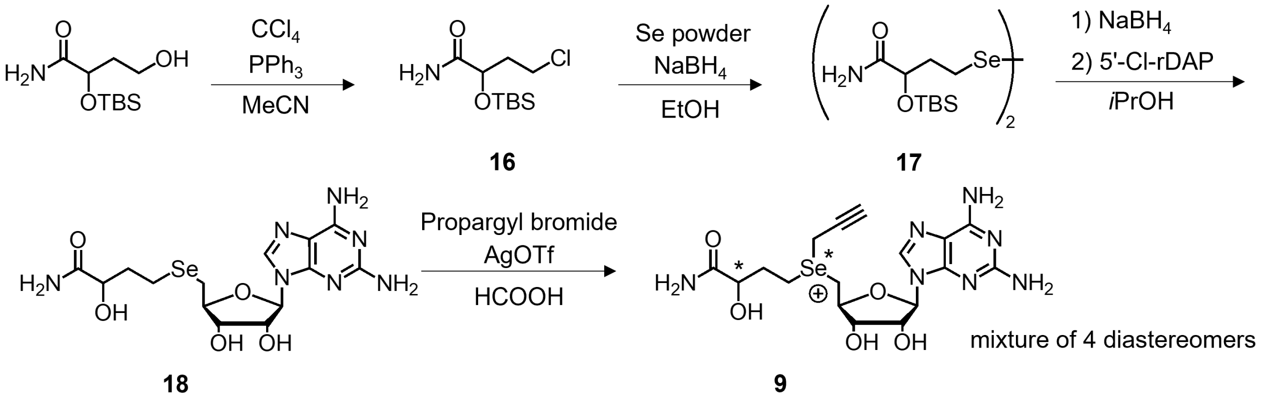

Supplementary Scheme 3. Synthesis of ProSeDMA OH

2-((*tert*-butyldimethylsilyl)oxy)-4-chlorobutanamide (16)

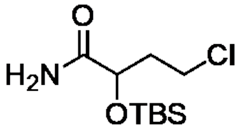

To a solution of 2-((tert-butyldimethylsilyl)oxy)-4-hydroxybutanamide (500 mg, 2.1mmol, obtained in two steps from α-hydroxy-γ-butyrolactone[2,3]) in dry acetonitrile (23 mL), CCl_4 (280 µL, 2.8 mmol) and triphenylphosphine (905mg, 3.5 mmol) were added under stirring at room temperature. The reaction was continued for 1 h with heating under reflux. After quenching with MeOH, solvent was removed by evaporation and the residue was extracted with EtOAc / saturated $NaHCO_3$ aq., water, brine. The organic layer was dried by Na_2SO_4 and evaporated. The crude product was purified by column chromatography on SiO_2 with DCM / *i*PrOH = 95 / 5. Yield: 381 mg of **16** as white solid (72%).

^{1}**H NMR** (400 MHz, DMSO-d6) δ 7.21 (s, 1H), 6.92 (s, 1H), 4.03 (t, J = 3.8 Hz, 1H), 3.66 – 3.50 (m, 3H), 1.97 – 1.83 (m, 2H), 0.80 (s, 9H), 0.00 (s, 6H). ^{13}**C NMR** (100 MHz, DMSO) δ 174.99, 70.79, 41.77, 38.54, 26.17, 18.37, -4.56, -4.78.

ESI-MS (*m/z*): $[M+Na]^+$ calcd for $C_{10}H_{22}ClNNaO_2Si$, 274.10005; found 274.09900.

4,4'-diselenediylbis(2-((*tert*-butyldimethylsilyl)oxy)butanamide) (17)

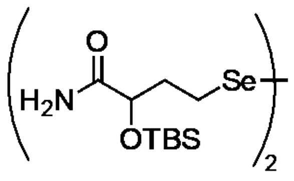

To a suspension of selenium powder (447 mg, 5.7 mmol) in ethanol (20 mL), sodium borohydride (144 mg, 3.8 mmol) was added. After stirring for 30 min at room temperature, compound **16** (477 mg, 1.9 mmol) was added in the solution and the reaction was allowed to proceed next 15 h. The solvent was switch to EtOAc and extracted by saturated $NaHCO_3$ aq., water, brine. The organic layer was dried by Na_2SO_4 and evaporated. The crude product was purified by column chromatography on SiO_2 with Hex / EtOAc = 1 / 1. Yield: 359 mg of **17** as slightly yellow foam (64 %).

^{1}H NMR (400 MHz, DMSO-d6) δ 7.21 (s, 1H), 6.83 (s, 1H), 3.98 (t, J = 5.6 Hz, 1H), 2.88 – 2.75 (m, 2H), 1.91 (dt, J = 7.4, 5.6 Hz, 2H), 0.81 (s, 9H), 0.00 (s, 6H). **^{13}C NMR** (100 MHz, DMSO) δ 174.93, 73.10, 37.26, 26.22, 24.75, 18.36, -4.56, -4.57.

ESI-MS (*m/z*): $[M+Na]^+$ calcd for $C_{20}H_{44}N_2NaO_4Se_2Si_2$ 615.10620; found 615.11124.

***Se*-2,6-Diaminopurineribosyl-2-hydroxy-4-selenobutanamide (18)**

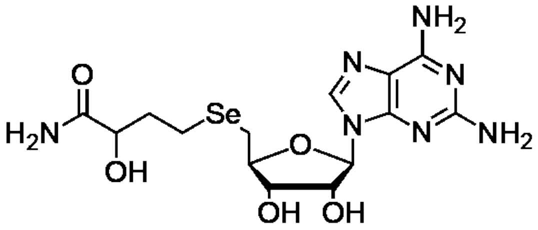

Compound **17** (10 mg, 17 µmol) was activated by sodium borohydride (6.1 mg, 160 µmol) in *i*PrOH (1 mL). After 15 min, 5'-chloro-2-aminoadenosine (9 mg, 30 µmol) was added and the reaction was proceeded for 2 h with heating under reflux. The reaction mixture was diluted by DCM and extracted water. The aqueous phase was dried by evaporation and the residue was dissolved in formic acid (1 mL). After overnight TBS-deprotection, the solvent was removed and the crude mixture was purified C18 reverse phase HPLC (2 mL/min, NUCLEODUR® C18 Pyramid VP 250/21 column, linear gradient B conc. 5% - 7% (0 min to 15 min), 7% - 70% (15min to 30 min); solvent A was H_2O + 0.1% TFA; solvent B was MeCN + 0.1% TFA; flow rate was 2 ml/min at 30 °C with UV detection at 260 nm.) Fractions containing the desired material were combined and removal of solvent by lyophilization. Yield: 6.12 µmol (determined by UV absorbance at 279 nm, $\varepsilon_{279\,nm}$ = 9,894 $Lmol^{-1}cm^{-1}$) of **18** as white foam (36 %).

^{1}**H NMR** (400 MHz, D_2O) δ 8.03 (s, 1H), 5.79 (d, J = 5.0 Hz, 1H), 4.79 – 4.76 (m, 1H), 4.31 – 4.28 (m, 1H), 4.23 – 4.19 (m, 1H), 4.07 – 4.03 (m, 1H), 2.92 – 2.88 (m, 2H), 2.65 – 2.46 (m, 2H), 1.96 – 1.74 (m, 2H). ^{13}**C NMR** (100 MHz, D_2O) δ 179.37, 152.31, 151.61, 149.87, 140.43, 111.58, 87.53, 84.01, 72.94, 72.90, 70.57, 34.29, 25.57, 19.75.

ESI-MS (*m/z*): $[M+Na]^+$ calcd for $C_{14}H_{21}N_7NaO_5Se$, 470.06616; found 470.06534.

***Se*-Propargyl-*Se*-2,6-diaminopurineribosyl-2-hydroxy-4-selenobutanamide (9)**

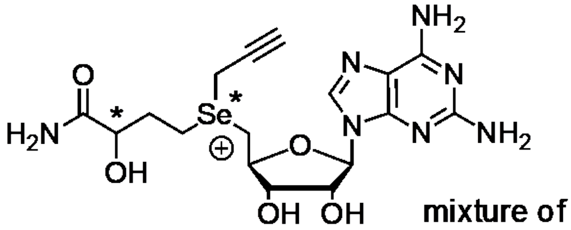

mixture of 4 diastereomer

Compound **18** (4.5 mg, 10 µmol) was dissolved in formic acid (1 mL) and the solution was cooled on ice bath. Propargyl bromide,80% in toluene, ca. 9.2 mol/L, 109 µL, 1 mmol and silver triflate (13 mg, 50 µmol) were added and the reaction mixture was stirred next 48 h. The reaction was quench by MeOH (500 µL) and the solvent was removed by evaporation. The residue was suspended in H_2O and insoluble Ag salt was filtered off. The crude mixture was purified C18 reverse phase HPLC (2 mL/min, NUCLEODUR® C18 Pyramid VP 250/21 column, linear gradient B conc. 5% - 7% (0 min to 15 min), 7% - 70% (15min to 30 min); solvent A was H_2O + 0.1% TFA; solvent B was MeCN + 0.1% TFA; flow rate was 2 ml/min at 30 °C with UV detection at 260 nm.) Fractions containing the desired material were combined and removal of solvent by lyophilization. Yield: 0.2 µmol (determined by UV absorbance at 279 nm, $\varepsilon_{279\ nm}$ = 9,894 $Lmol^{-1}cm^{-1}$) of **9** as white foam (2 %).

^{1}**H NMR** (400 MHz, D_2O) δ 8.07 – 7.94 (m, 1H), 5.91 – 5.77 (m, 1H), 4.83 – 4.70 (m, 1H), 4.60 – 4.51 (m, 1H), 4.44 – 4.31 (m, 1H), 4.23 – 4.13 (m, 1H), 4.11 – 3.88 (m, 2H), 3.87 – 3.61 (m, 2H), 3.48 – 3.30 (m, 2H), 3.16 – 2.95 (m, 1H), 2.29 – 1.90 (m, 2H). ^{13}**C NMR** (100 MHz, D_2O) δ 177.84, 152.19, 151.50, 149.84, 140.84, 114.83, 89.33, 79.94, 79.40, 73.41, 72.92, 71.05, 69.89, 40.89, 36.12, 28.60, 25.46.

ESI-MS (*m/z*): $[M]^+$ calcd for $C_{17}H_{24}N_7O_5Se$, 486.09986; found 486.10022.

Synthesis of *N*^{6}-propargyl-adenosine[4]

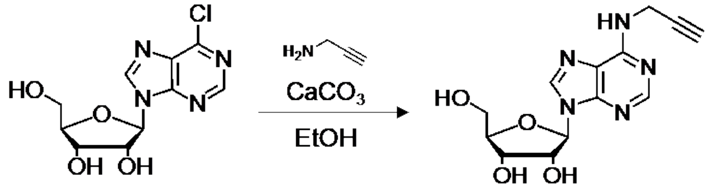

6-Chloropurine nucleoside (500 mg, 1.7 mmol) was suspended in ethanol (125 mL) and calcium carbonate (350 mg, 3.5 mmol) and propargylamine (0.6 mL, 8.8 mmol) were added. The mixture was refluxed for 12 hours. Then the excessive $CaCO_3$ was removed by filtration, and filtrate was cooled at -20 °C for 1 h. The white precipitate was collected and dried in vacuo. The precipitate was purified by re-crystallization in ethanol. Yield: 400 mg of *N*^{6}-propargyl-adenosine as white solid (79 %). Analytical data in agreement with previous reports[4]

Synthesis of 1-propargyl-adenosine[5]

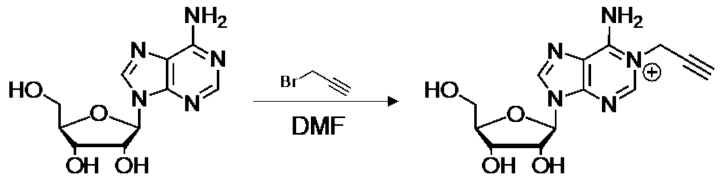

Adenosine (200 mg, 0.75 mmol) was suspended in DMF (0.5 mL) and propargyl bromide (80% in toluene) (0.3 mL, 3.8 mmol) was added to a solution. After stirring at 50 °C for 24 h, the solvent was removed under reduced pressure. The residue was purified by column chromatography on SiO_2 with DCM / MeOH = 8 / 2. Yield: 120 mg of *N1*-propargyl-adenosine as brown form (52 %).

Analytical data are in agreement with previous reports[5]

Synthesis of 2'-*O*-propargyl-adenosine[6]

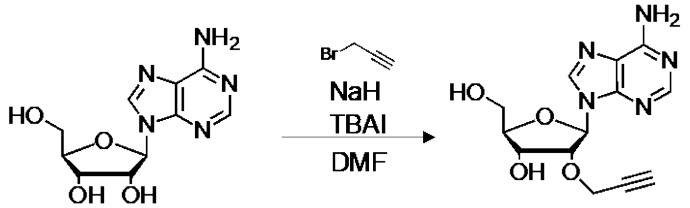

Adenosine (200 mg, 0.75 mmol) was suspended in DMF (0.5 mL) and solution was cooled by ice bath. NaH (40 mg, 0.9 mmol, 60% dispersion in mineral oil) was added, followed by the addition of TBAI (60 mg, 0.16 mmol) and propargyl bromide (80% in toluene) (66 µL, 0.83 mmol). After stirring at

60 °C for 24 h, the solvent was removed under reduced pressure. The residue was purified by column chromatography on SiO_2 with DCM / MeOH = 93 / 7. Yield: 73 mg of 2'-*O*-Propargyl-adenosine as yellow solid (32 %). Analytical data are in agreement with previous reports[6]

Synthesis of S-(2,6-Diaminopurine-ribosyl)-Methionine[7]

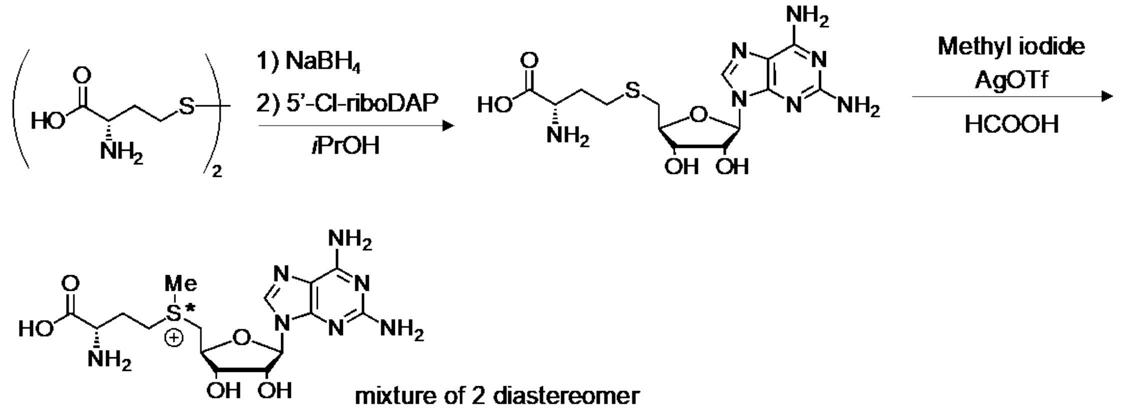

L-homocystine (10 mg, 4 µmol) was activated by sodium borohydride (1.5 mg, 40 µmol) in *i*PrOH (200 µL). After 1 h, 9-(5'-chloro-5'-deoxy-β-D-ribofuranosyl)-2,6-diamiopurine (= 5'-chloro-2-aminoadenosine) (2 mg, 6 µmol) was added and the reaction was proceeded for 2 h with heating under reflux. The reaction mixture was diluted by DCM and extracted with water. The aqueous phase was dried by evaporation and the residue was dissolved in formic acid (200 mL) and the solution was cooled in an ice bath. Methyl iodide (12.4 µL, 200 µmol) and silver triflate (2.6 mg, 10 µmol) were added and the reaction mixture was stirred for 48 h. The reaction was quenched by MeOH (500 µL) and the solvent was removed by evaporation. The residue was suspended in H_2O and insoluble Ag salt was filtered off. The crude mixture was purified by C18 reverse phase HPLC (2 mL/min, NUCLEODUR® C18 Pyramid VP 250/21 column, linear gradient B conc. 5% - 7% (0 min to 15 min), 7% - 70% (15min to 30 min); solvent A was H_2O + 0.1% TFA; solvent B was MeCN + 0.1% TFA; flow rate was 2 ml/min at 30 °C with UV detection at 260 nm.) Fractions containing the desired material were combined and the solvent was removed by lyophilization. The target product was identified by LC/MS analysis. **ESI-MS** (*m/z*): $[M]^+$ calcd for $C_{15}H_{24}N_7O_5S^+$, 414.1554; found 414.1610. Yield: 0.4 µmol (determined by UV absorbance at 279 nm, $\varepsilon_{279\ nm}$ = 9,894 $Lmol^{-1}cm^{-1}$) as white foam (6.6 %). Analytical data are in agreement with previous reports.[8]

4. NMR spectra

Compound **2** ^{1}H NMR

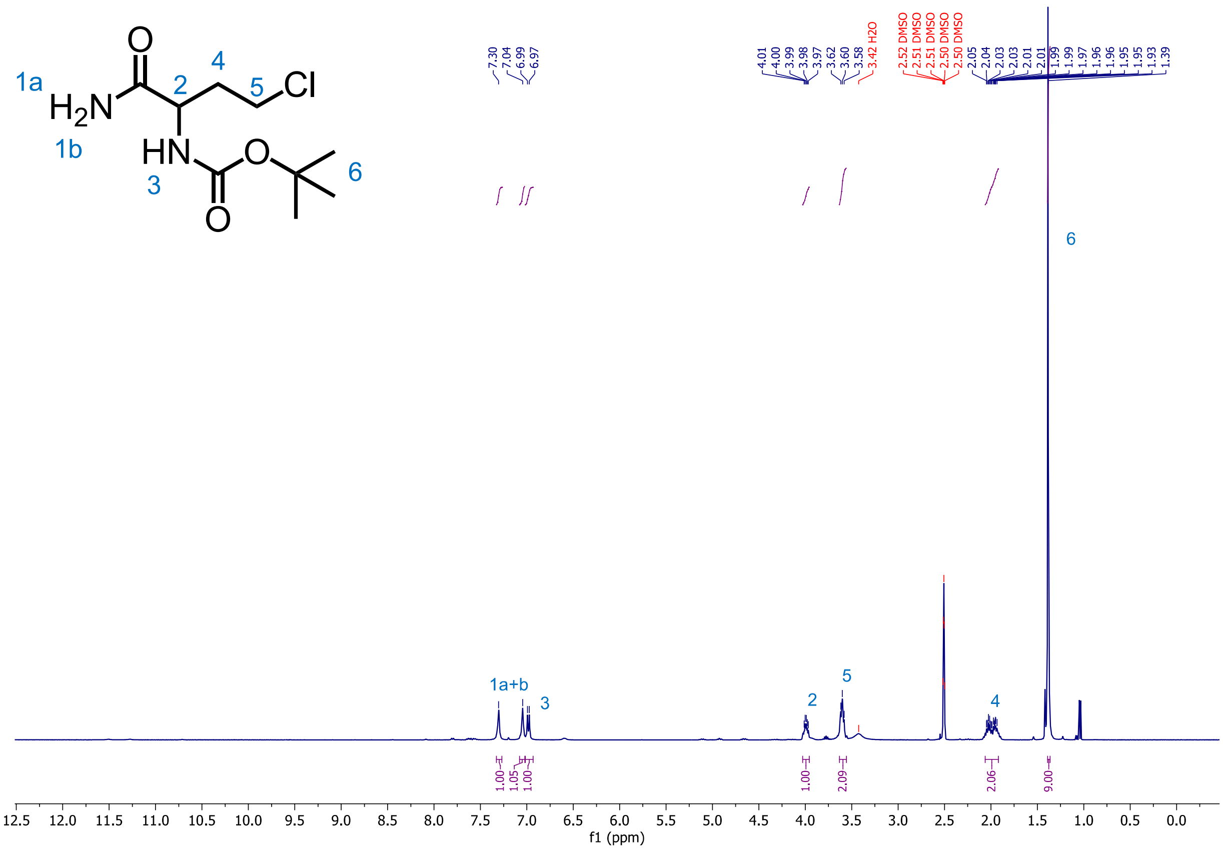

Compound **2** ^{13}C NMR

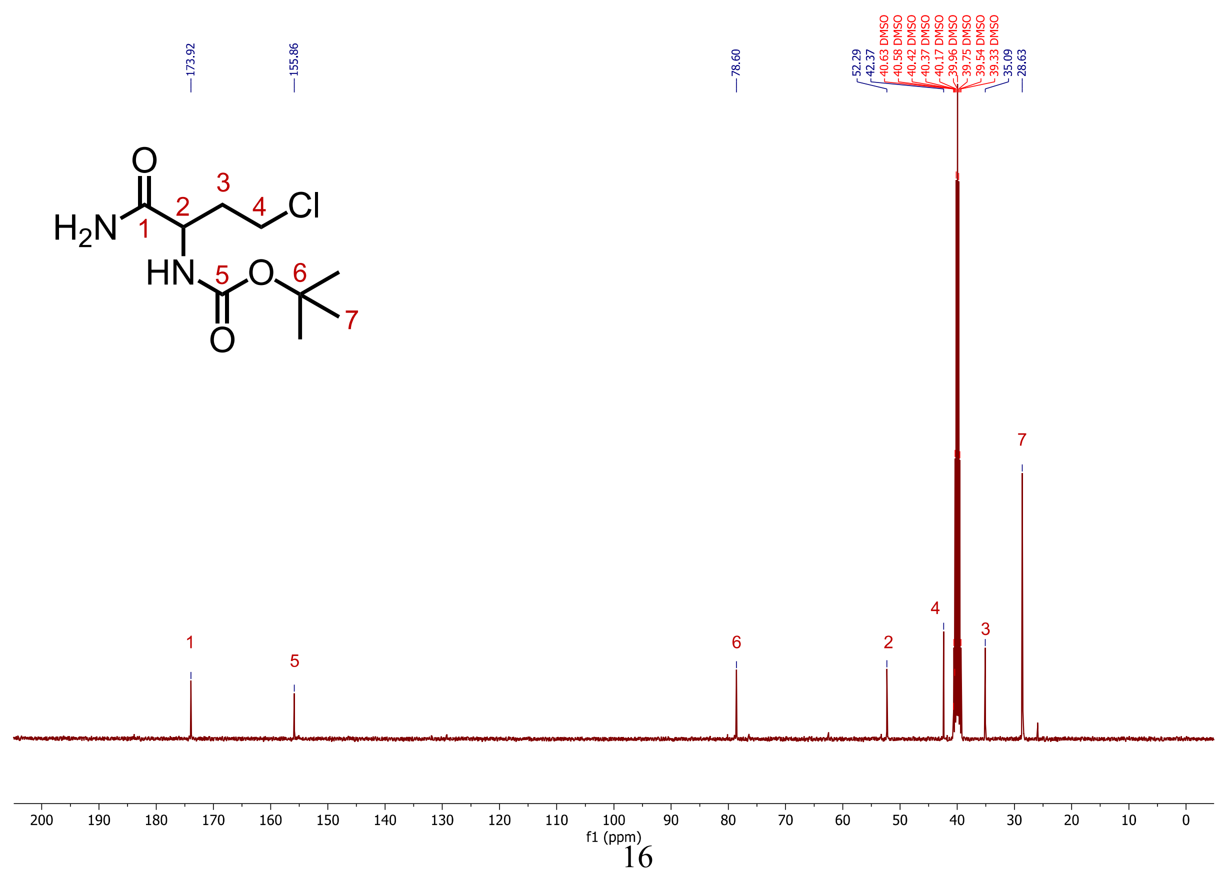

Compound **3** ^{1}H NMR

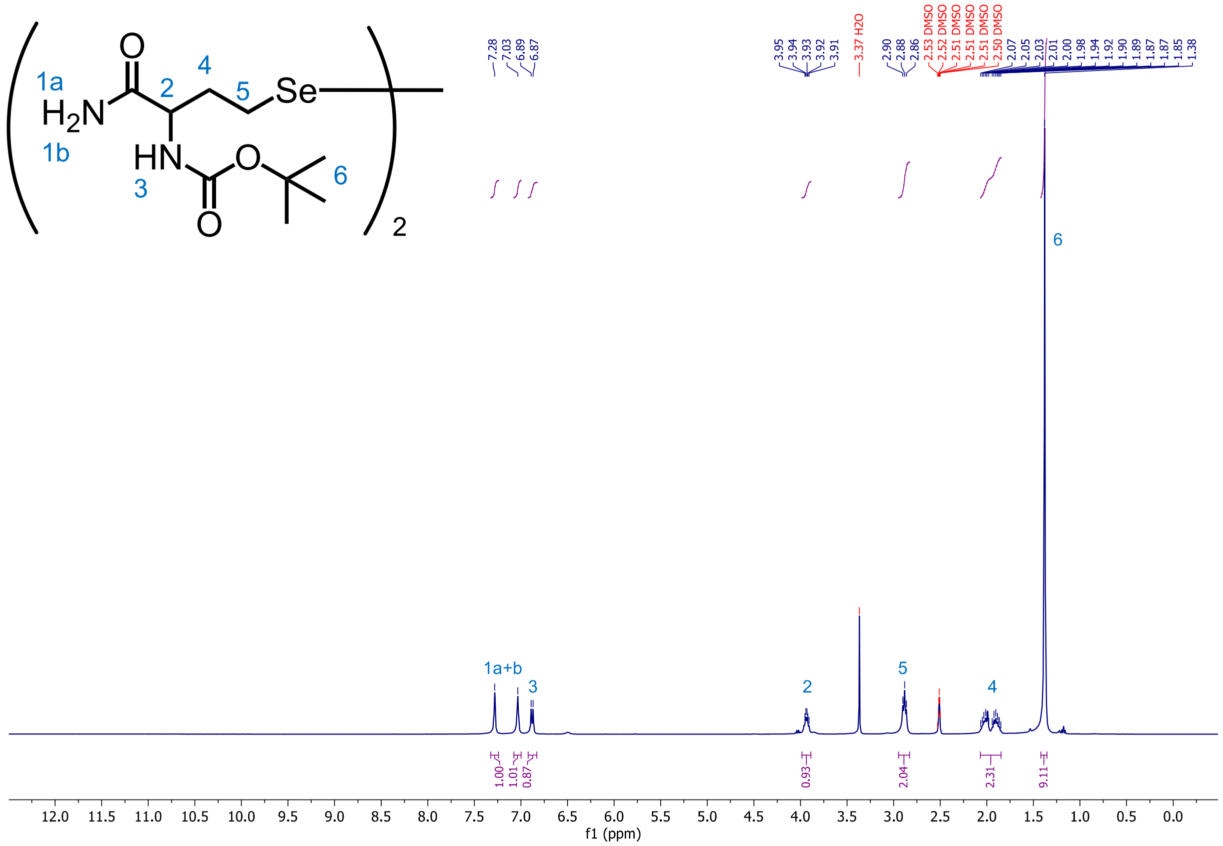

Compound **3** ^{13}C NMR

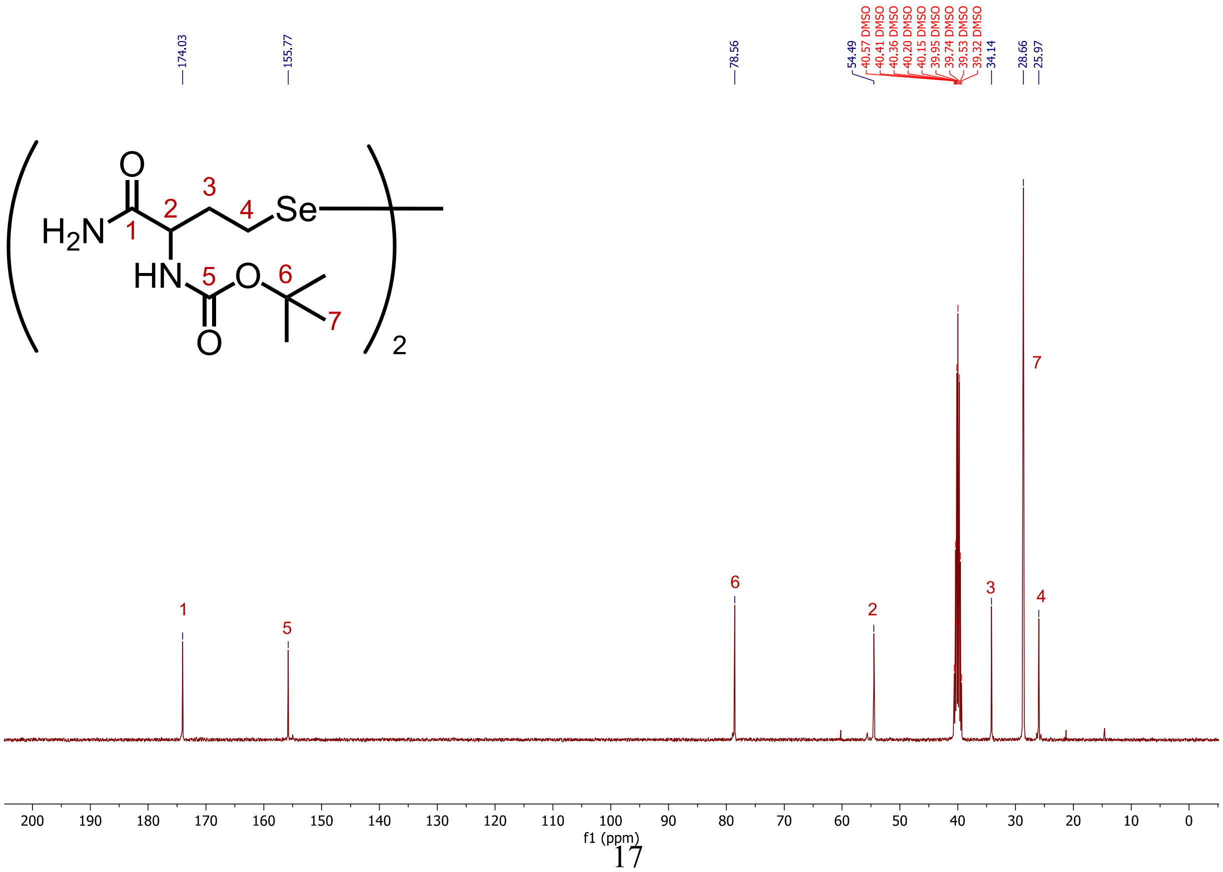

Compound **4** ^{1}H NMR

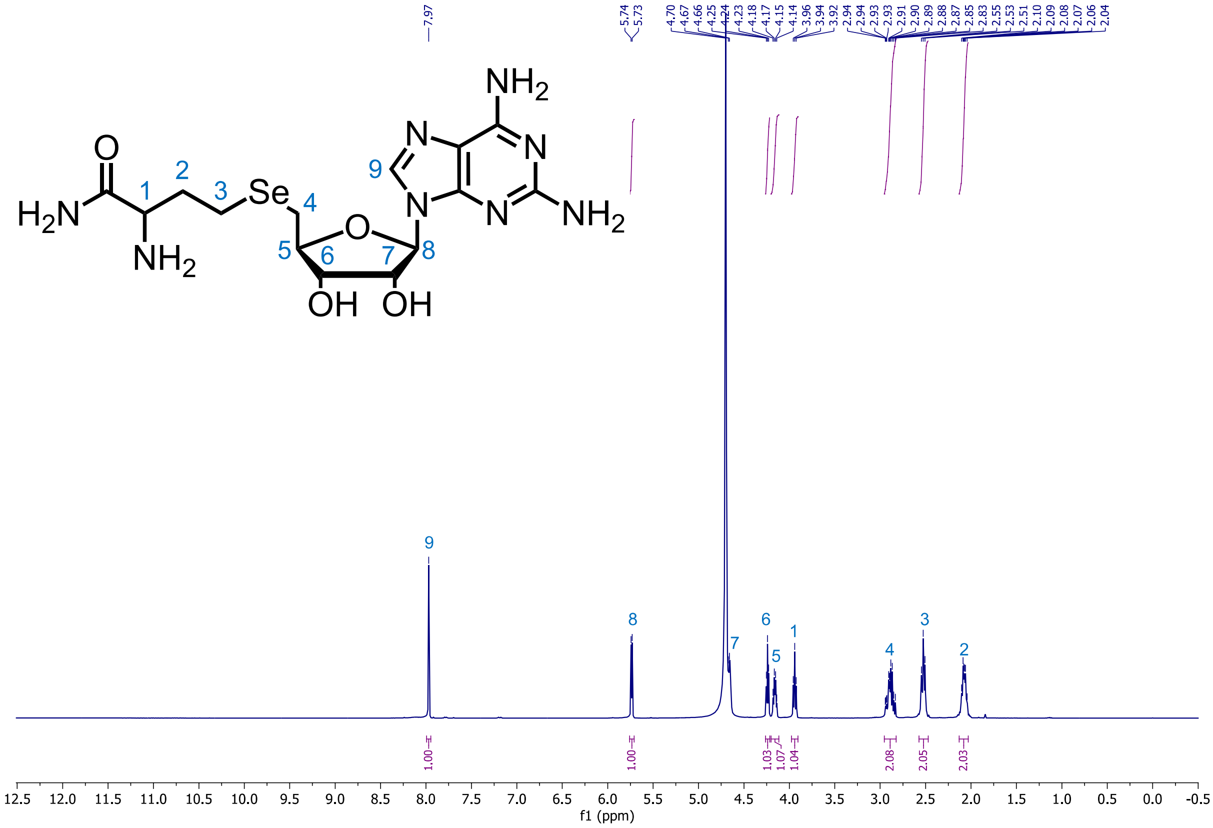

Compound **4** ^{13}C NMR

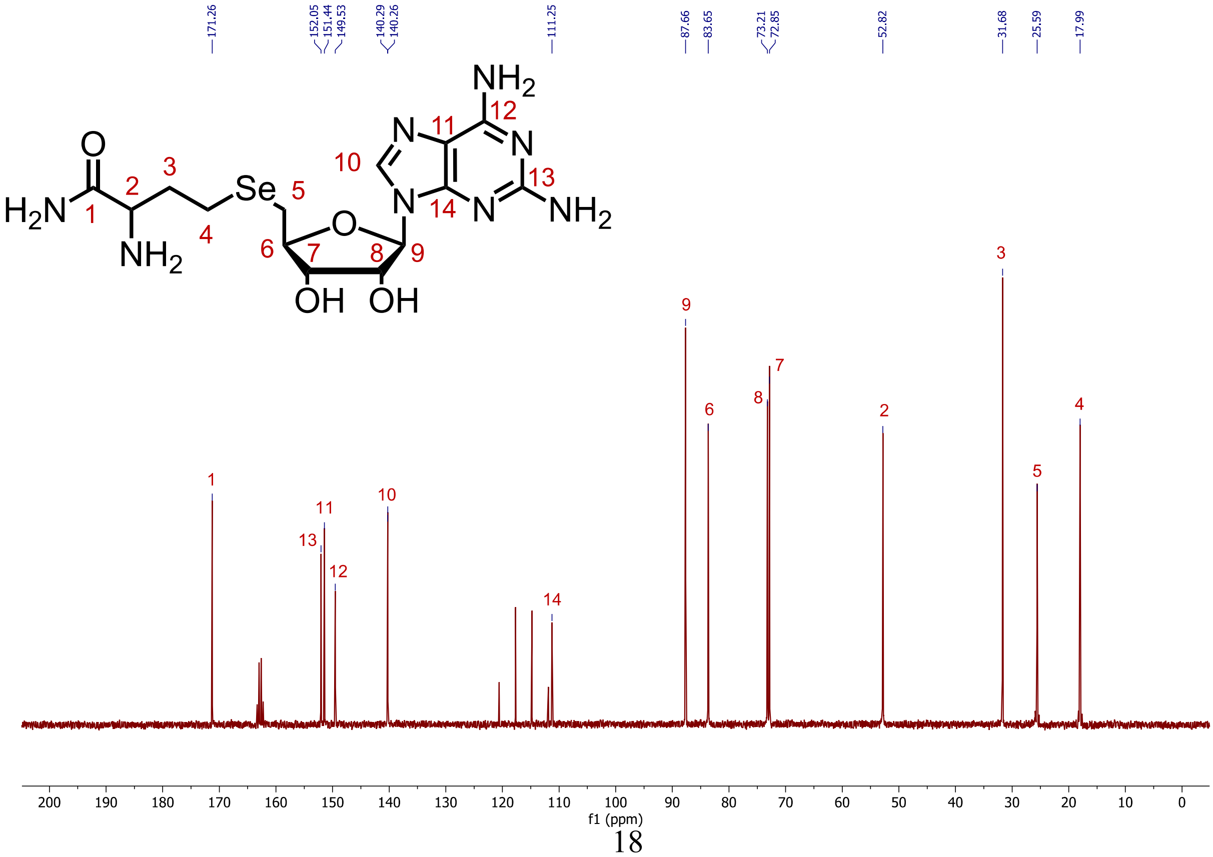

Compound **5** ^{1}H NMR

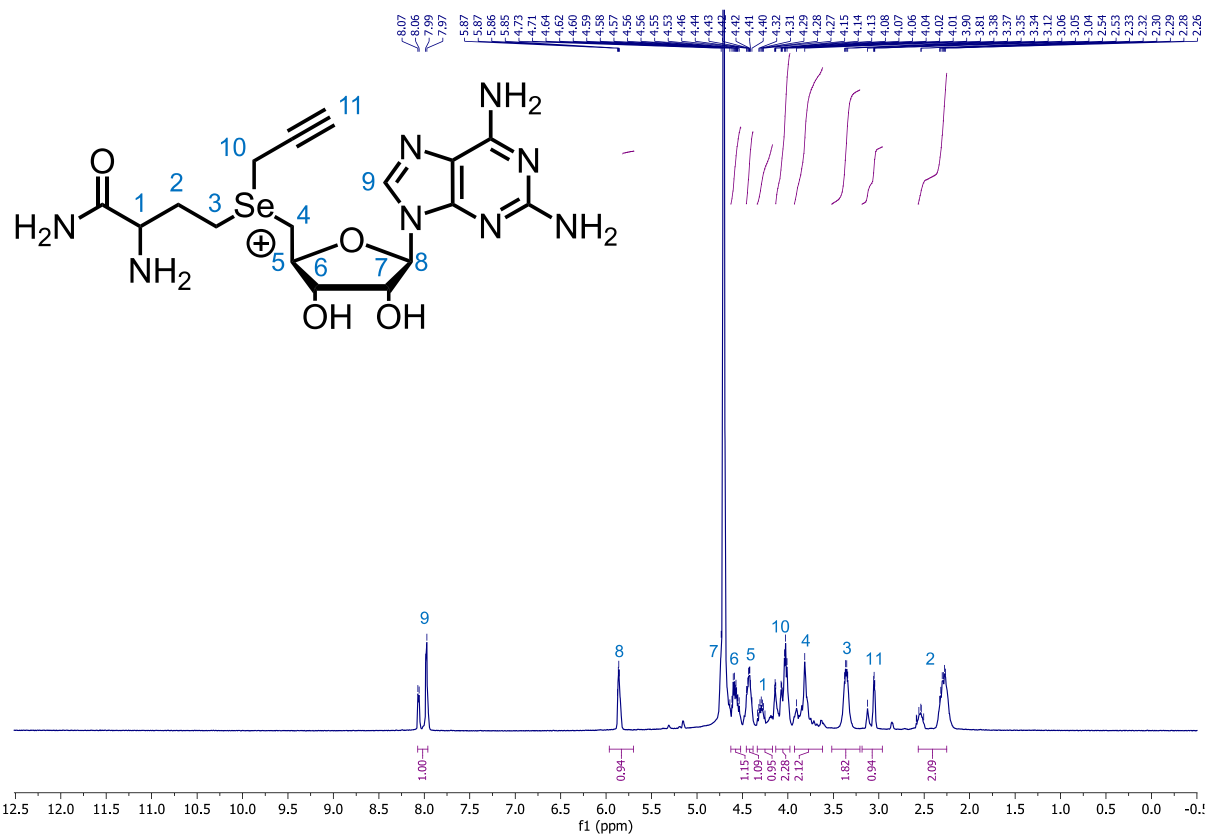

Compound **5** ^{13}C NMR

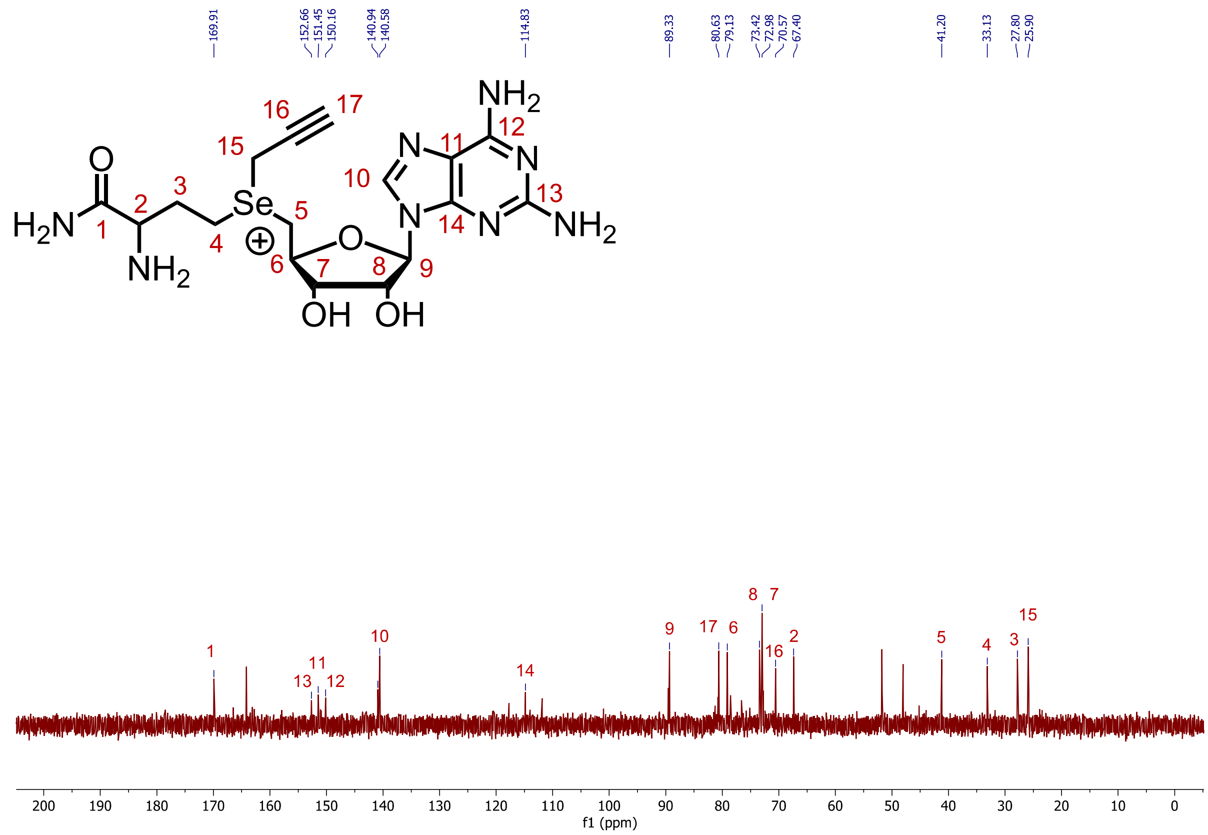

Compound **5** HSQC

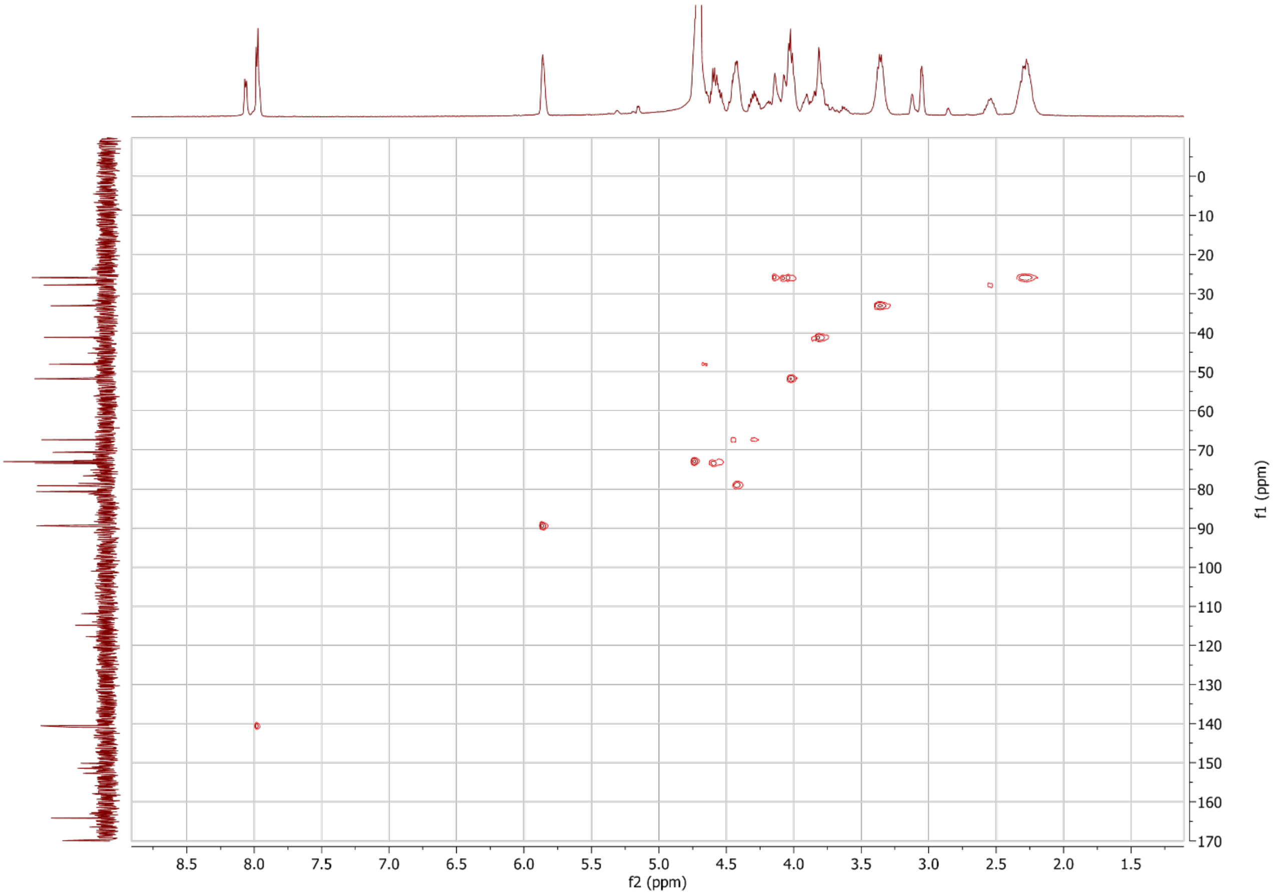

Compound **5** HMBC

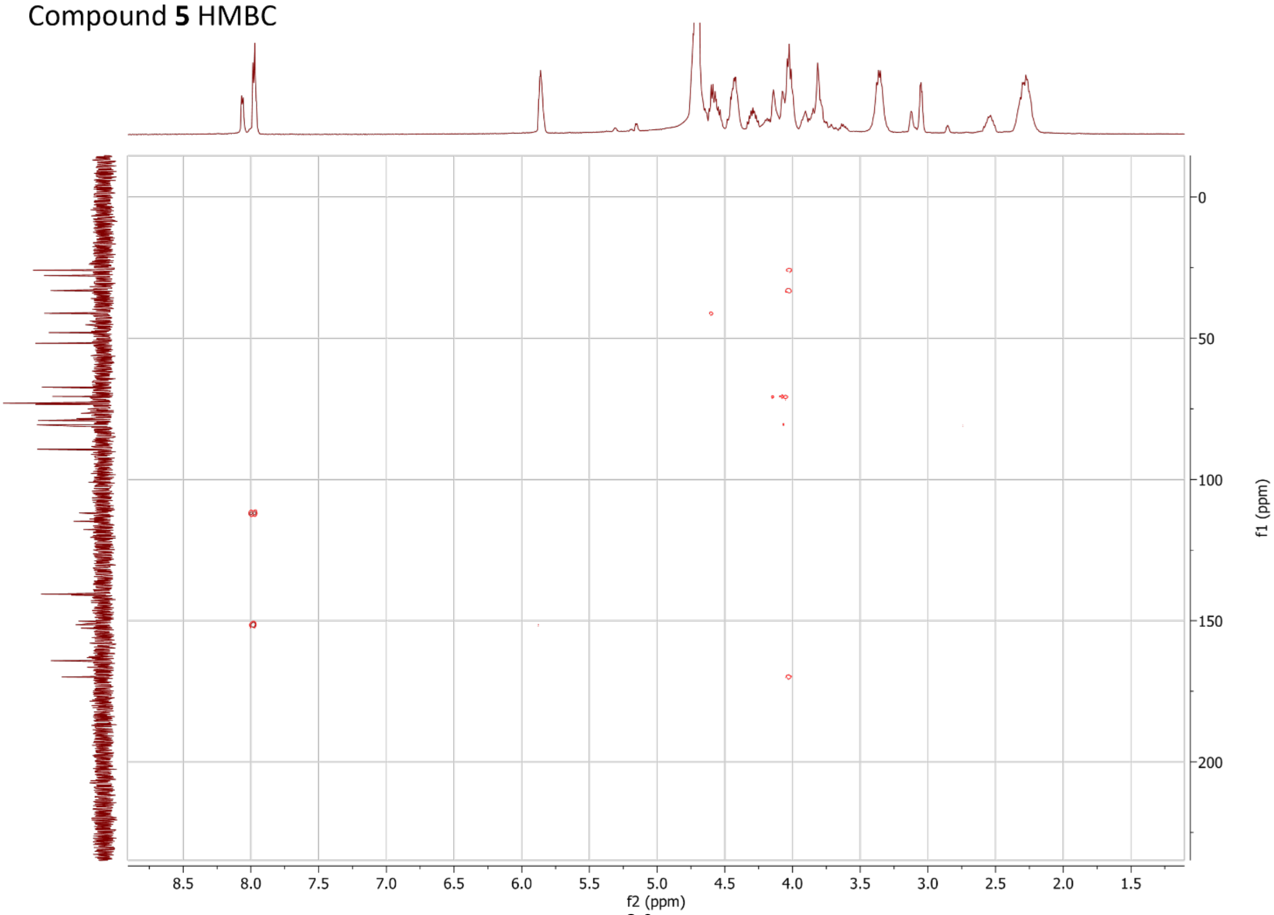

Compound **6** ^{1}H NMR

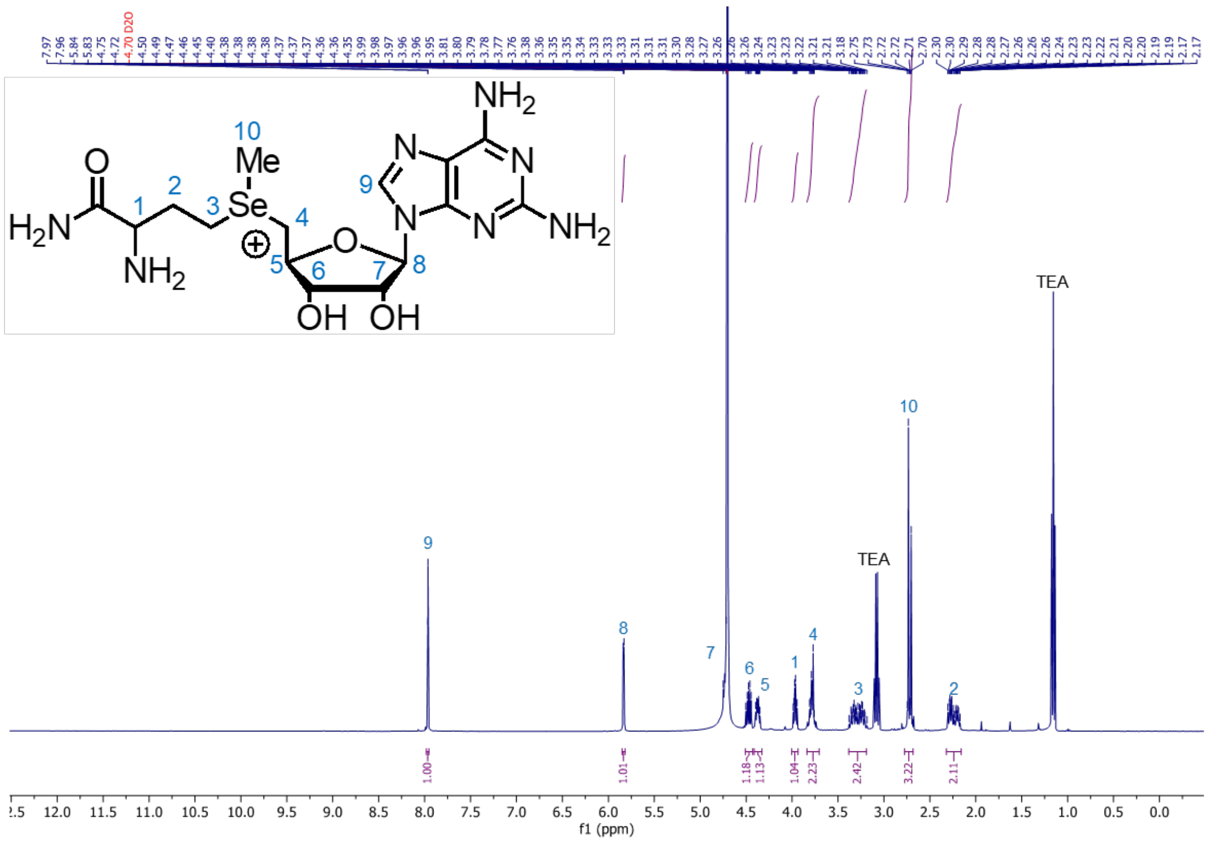

Compound **6** ^{13}C NMR

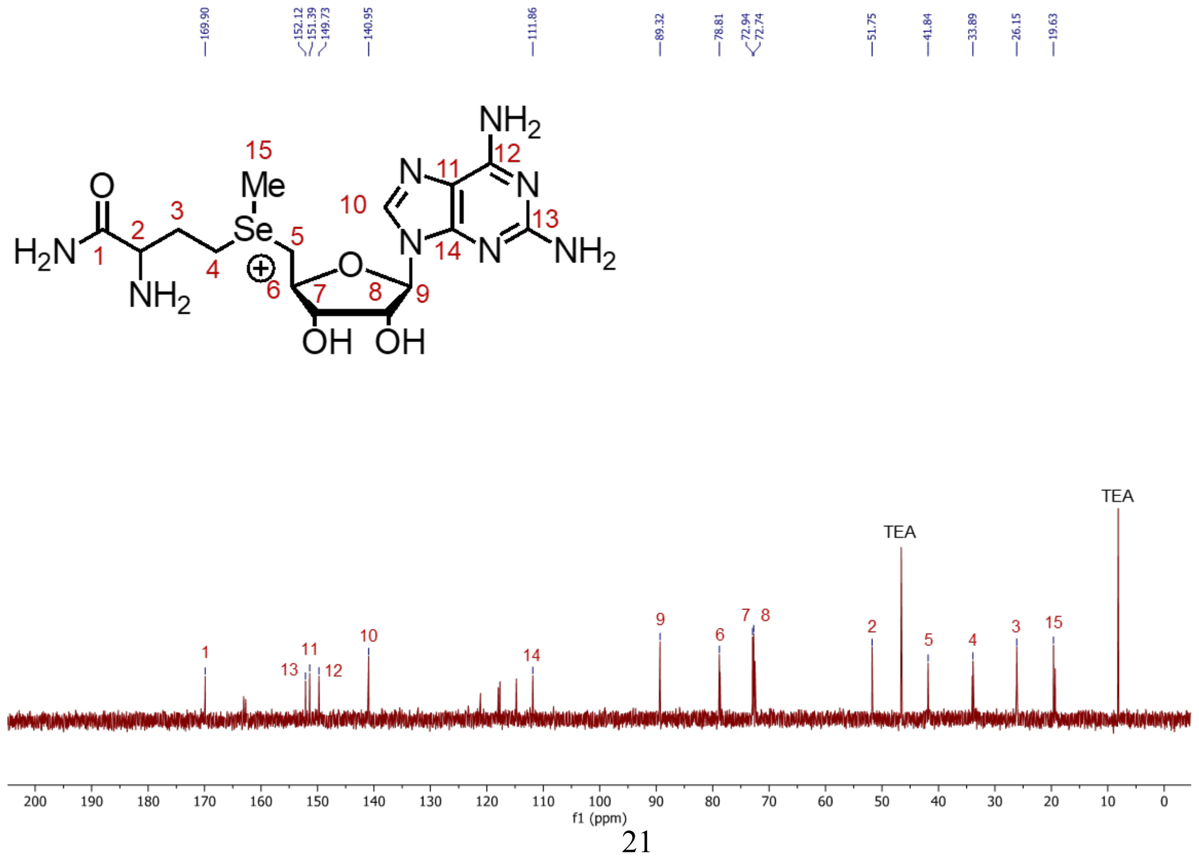

Compound **6** HSQC

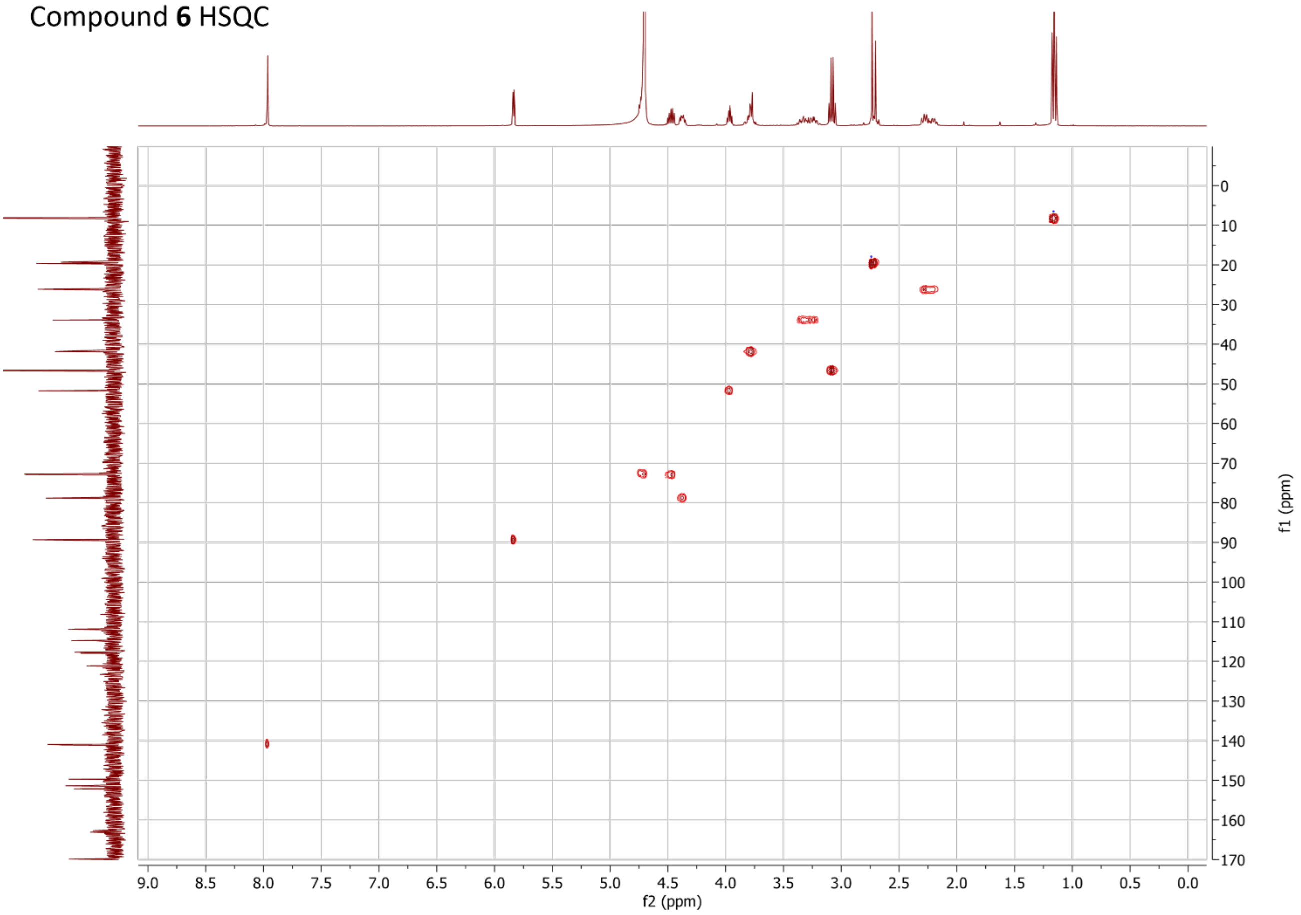

Compound **6** HMBC

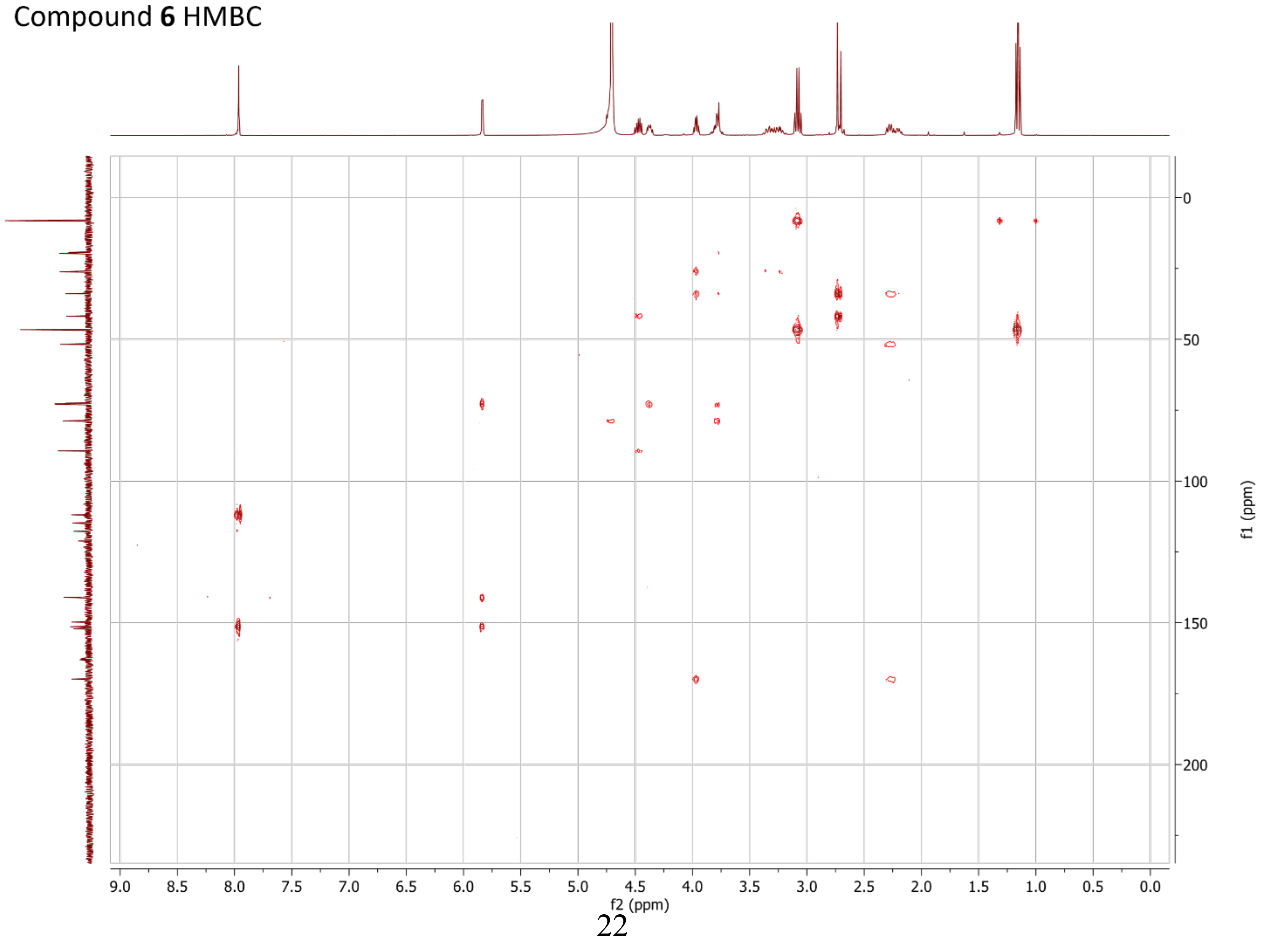

Compound **7** ^{1}H NMR

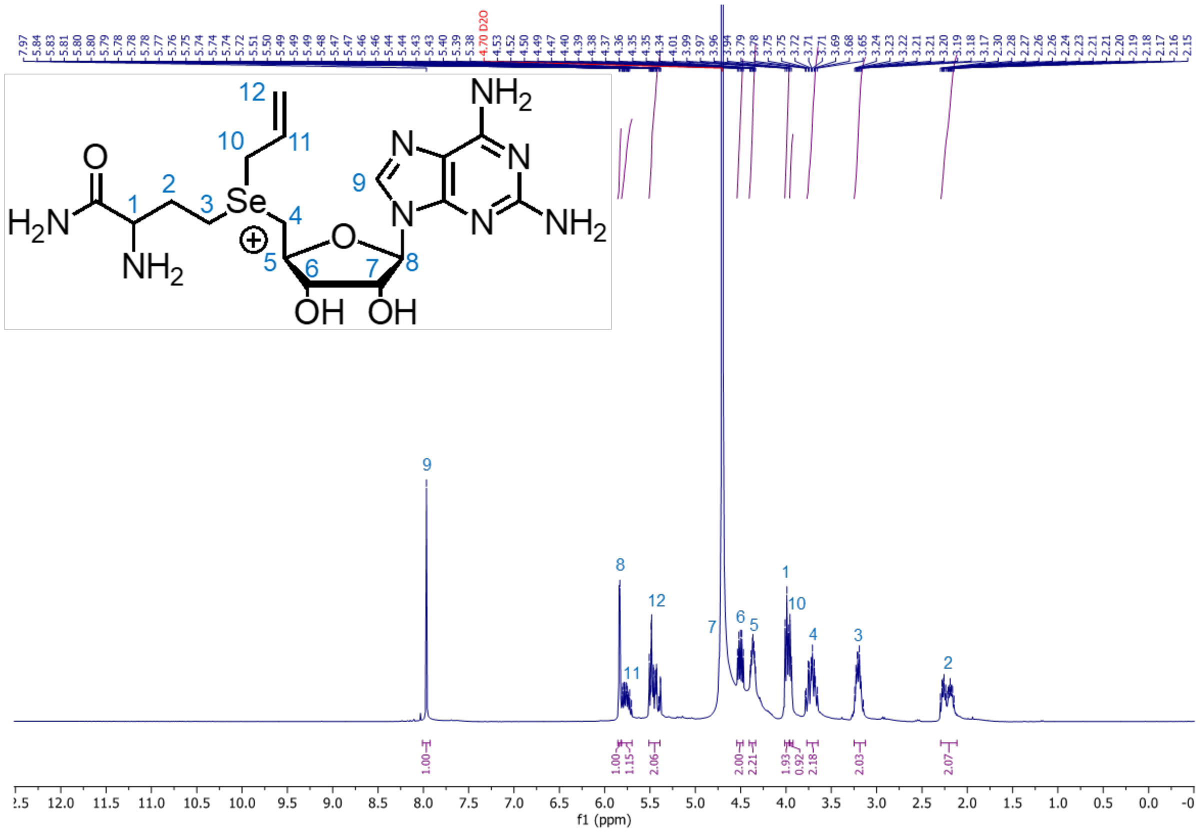

Compound **7** ^{13}C NMR

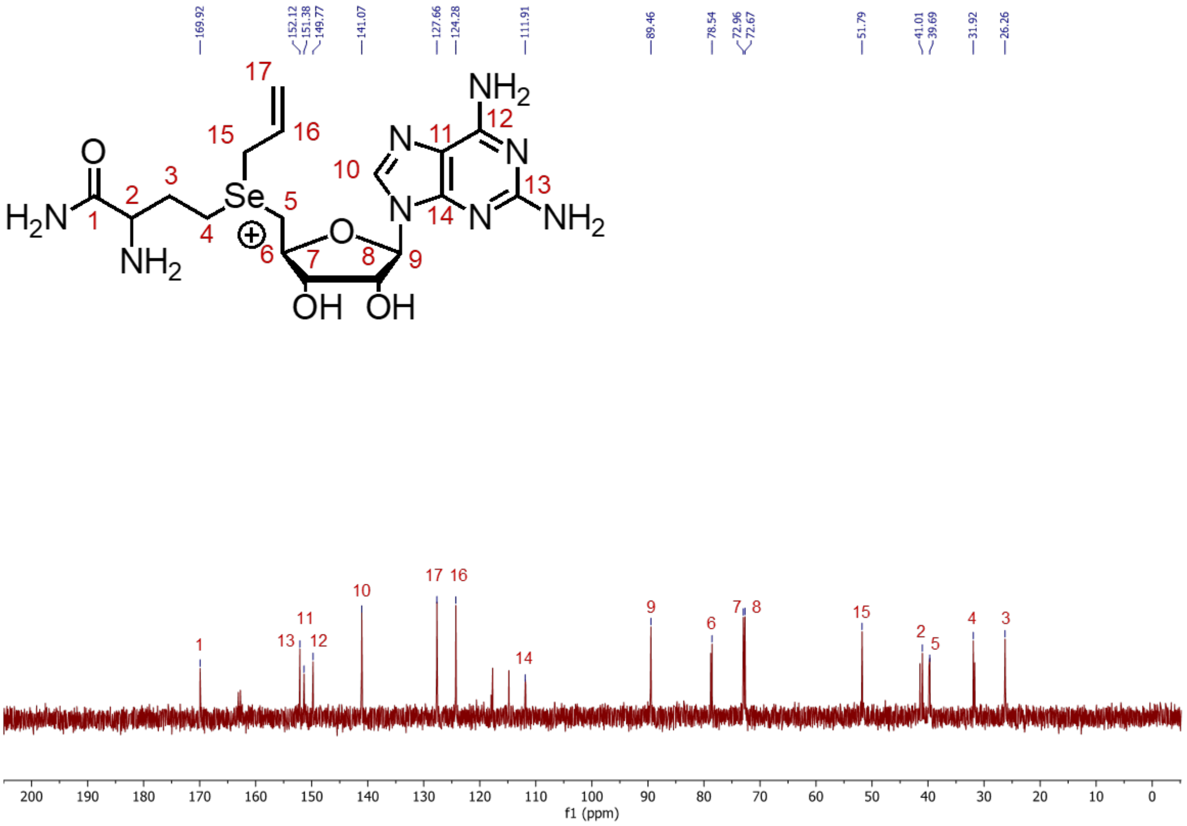

Compound **7** HSQC

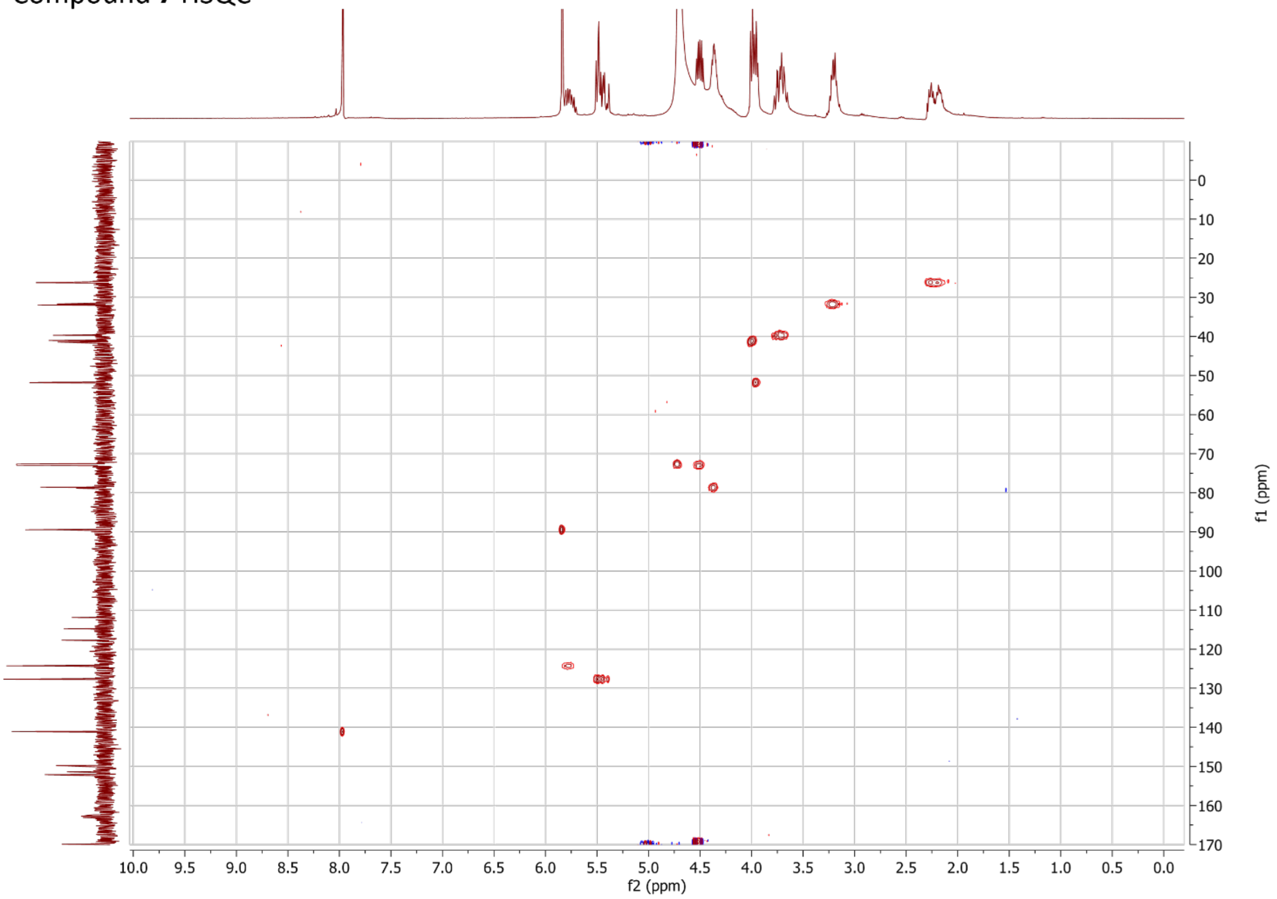

Compound **7** HMBC

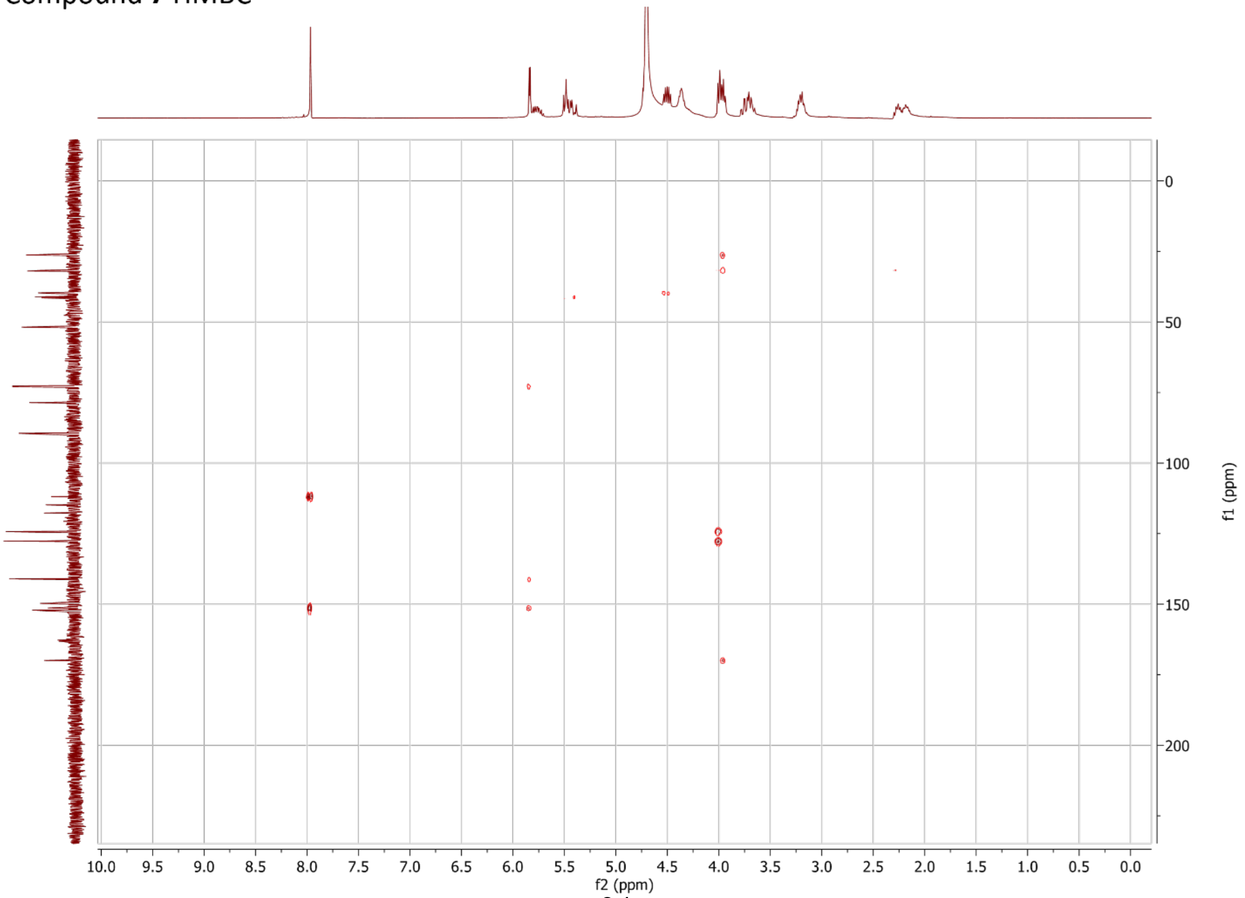

Compound **8** ^{1}H NMR

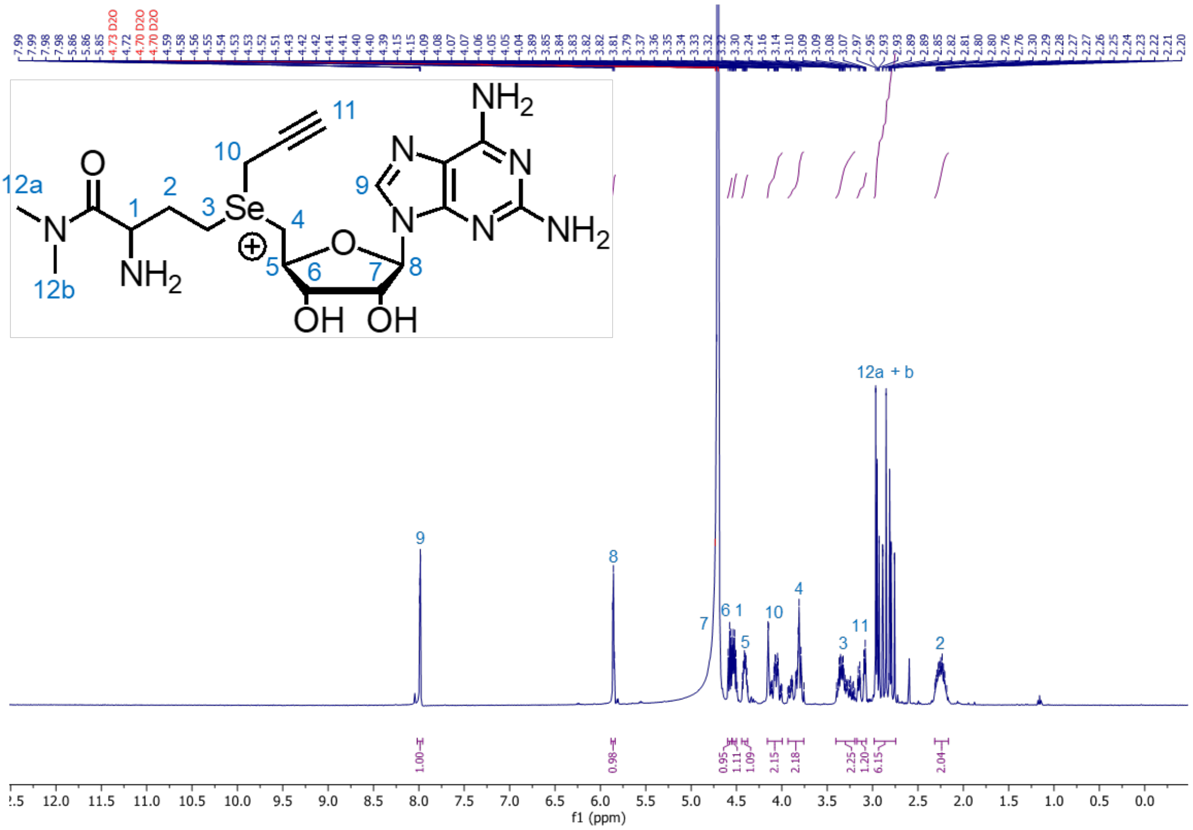

Compound **8** ^{13}C NMR

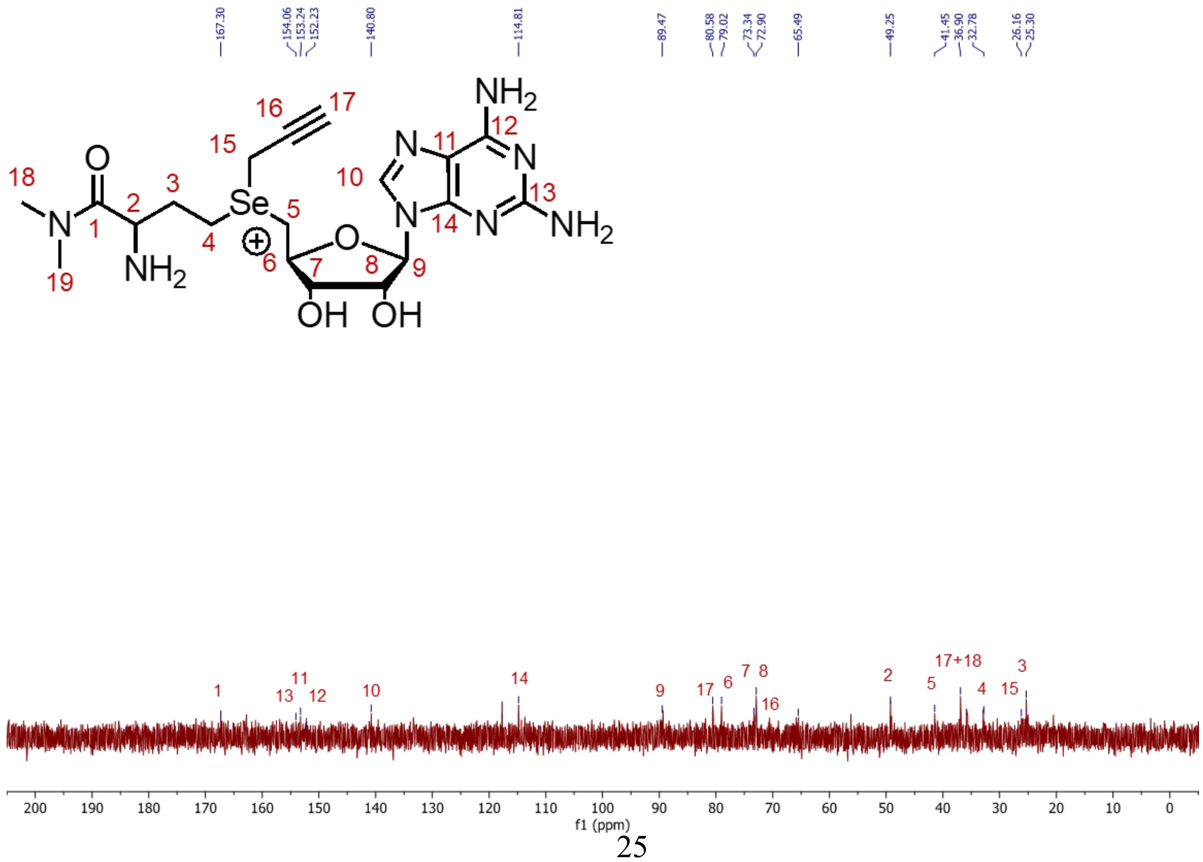

Compound **8** HSQC

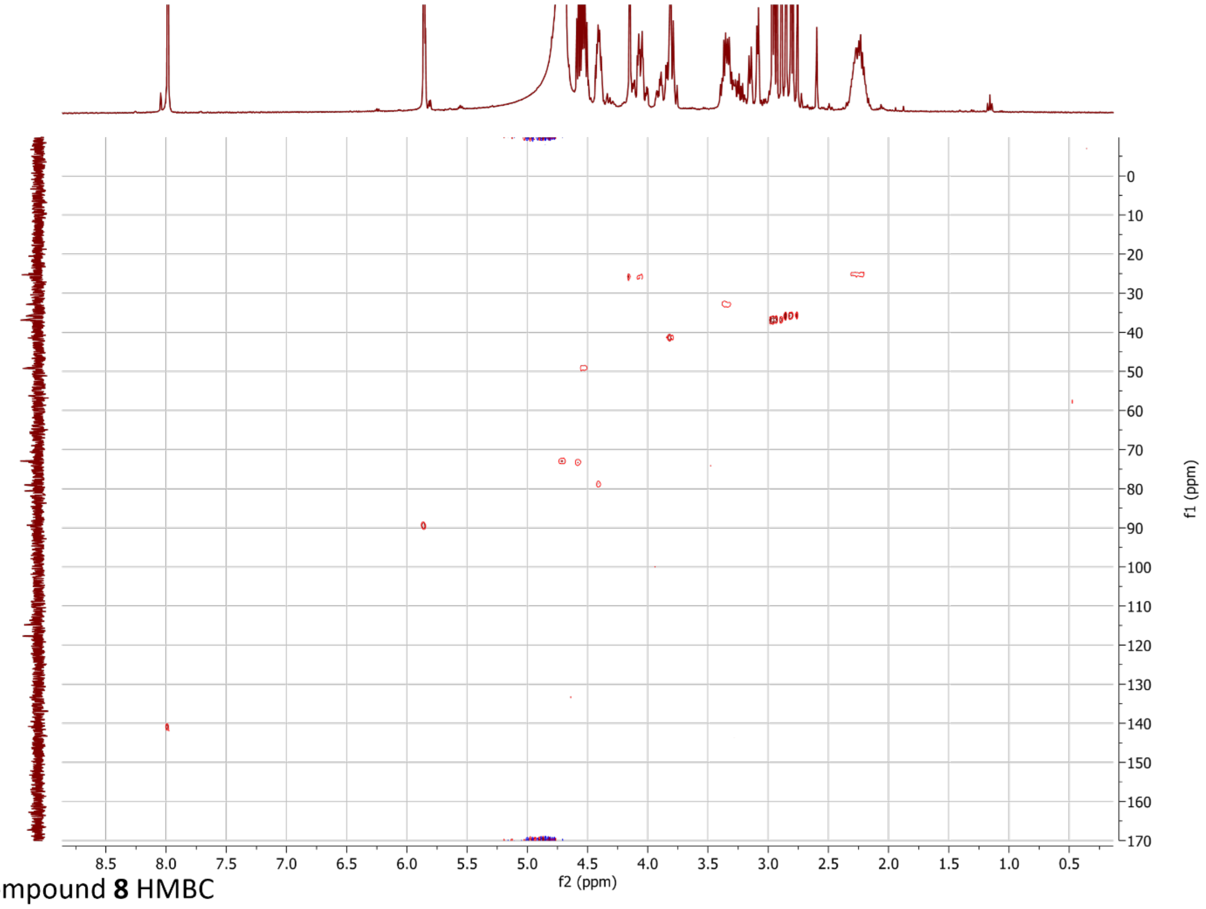

Compound **8** HMBC

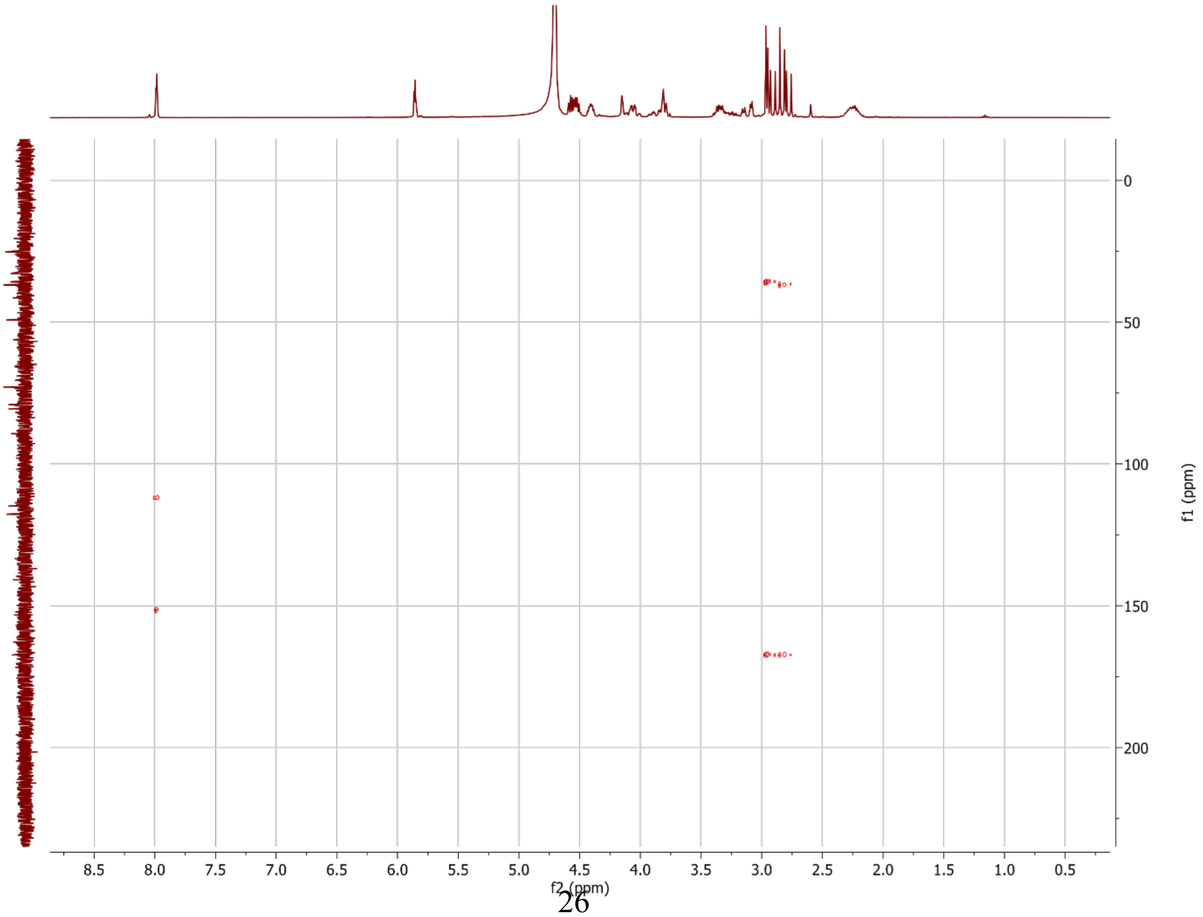

Compound **9** ^{1}H NMR

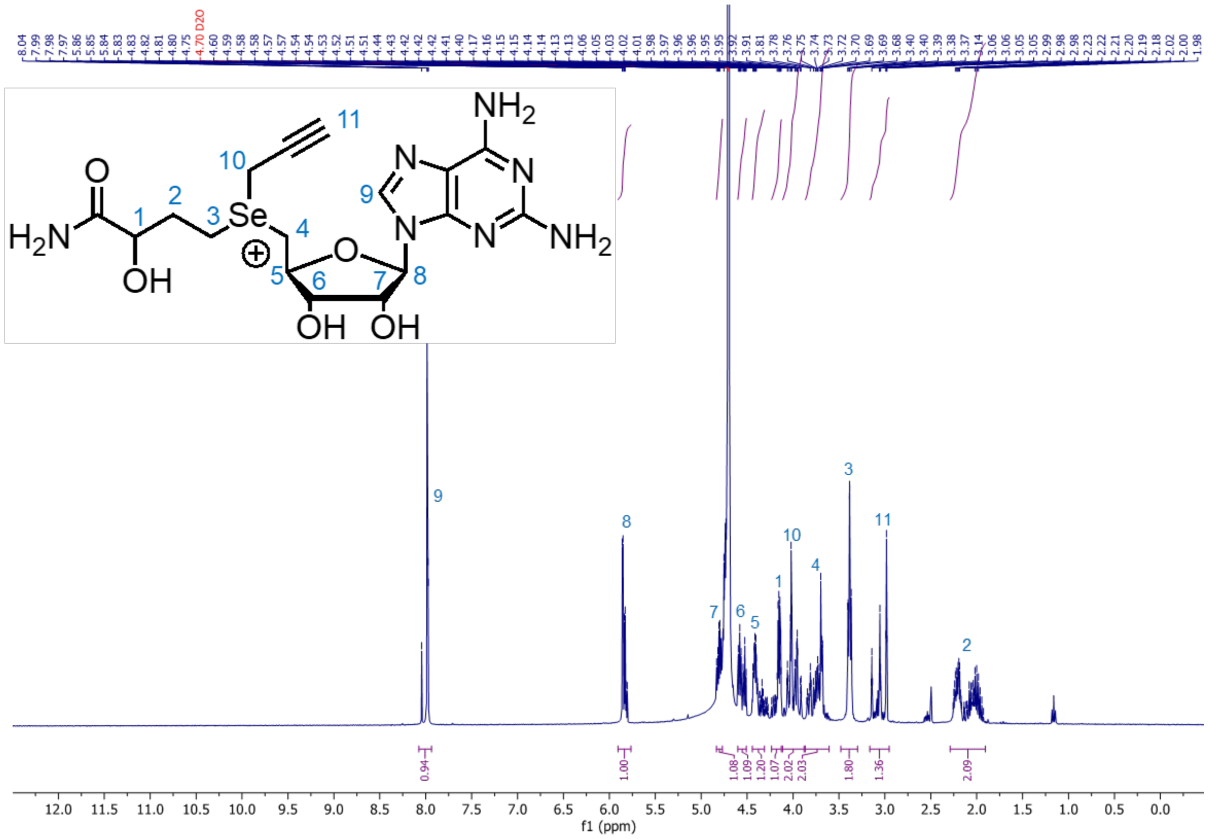

Compound **9** ^{13}C NMR

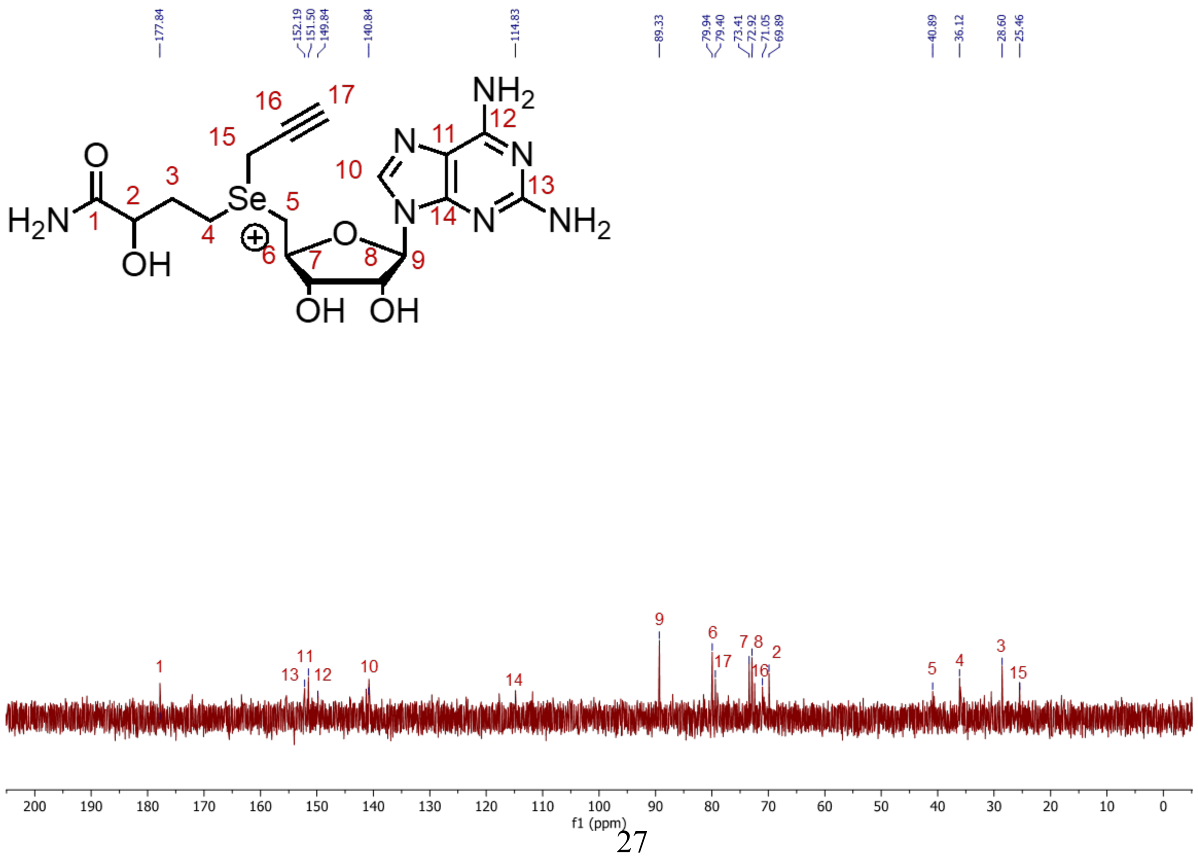

Compound **9** HSQC

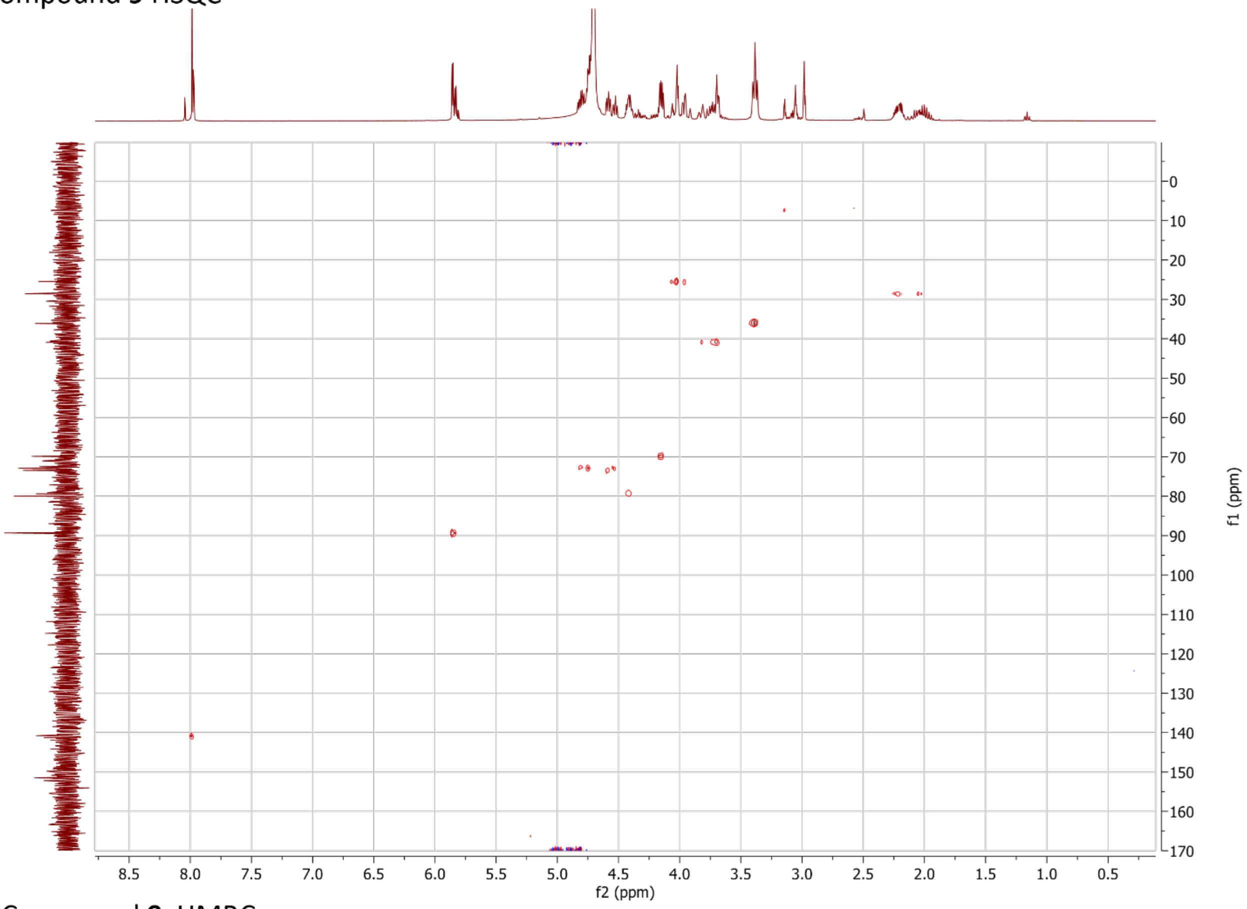

Compound **9** HMBC

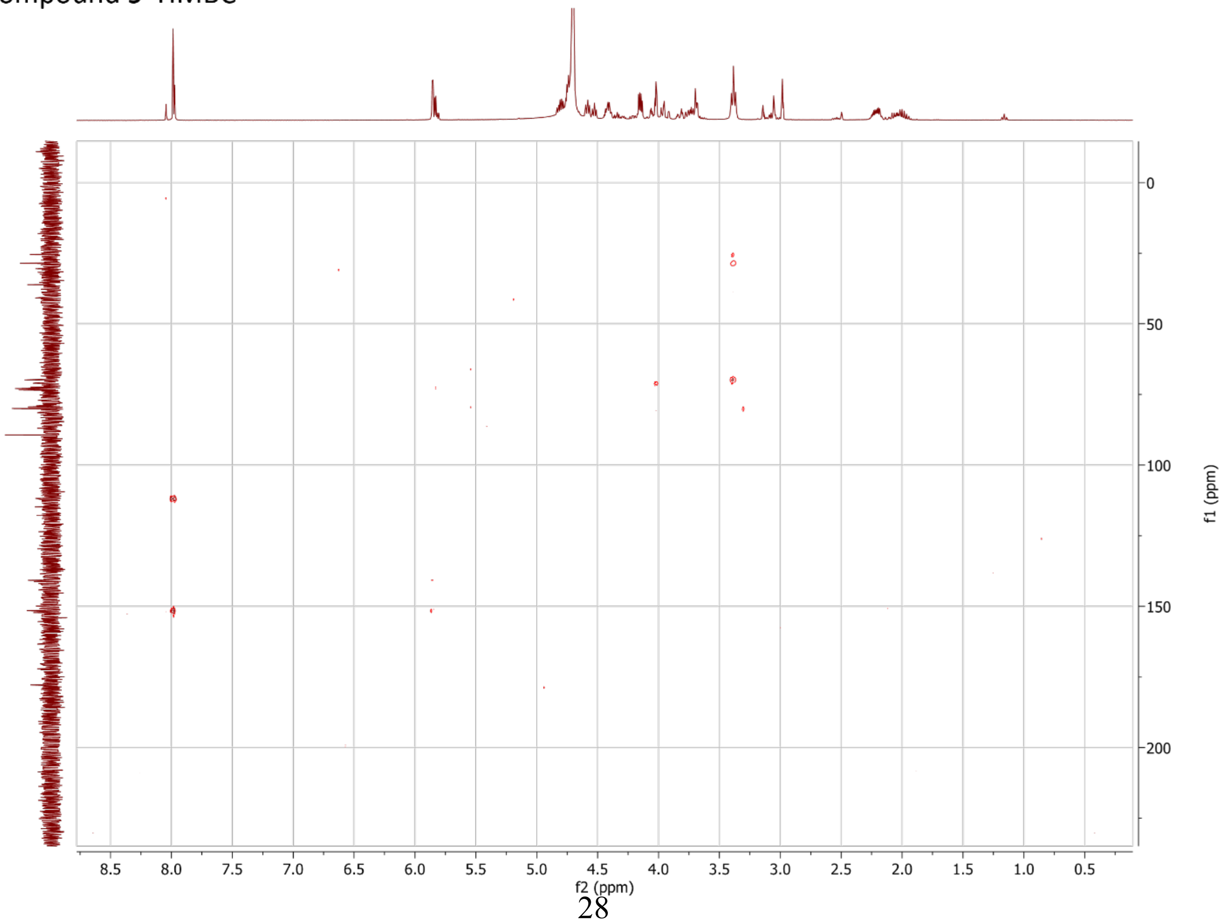

Compound **13** ^{1}H NMR

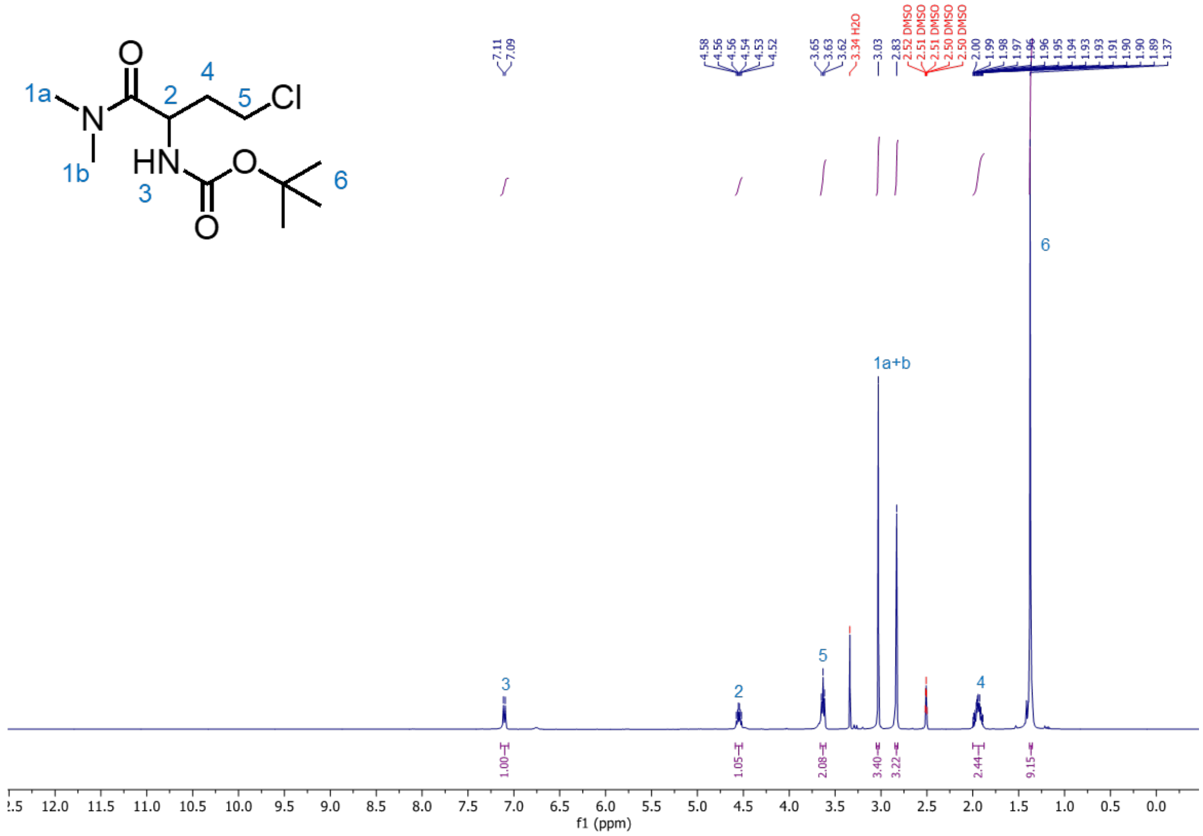

Compound **13** ^{13}C NMR

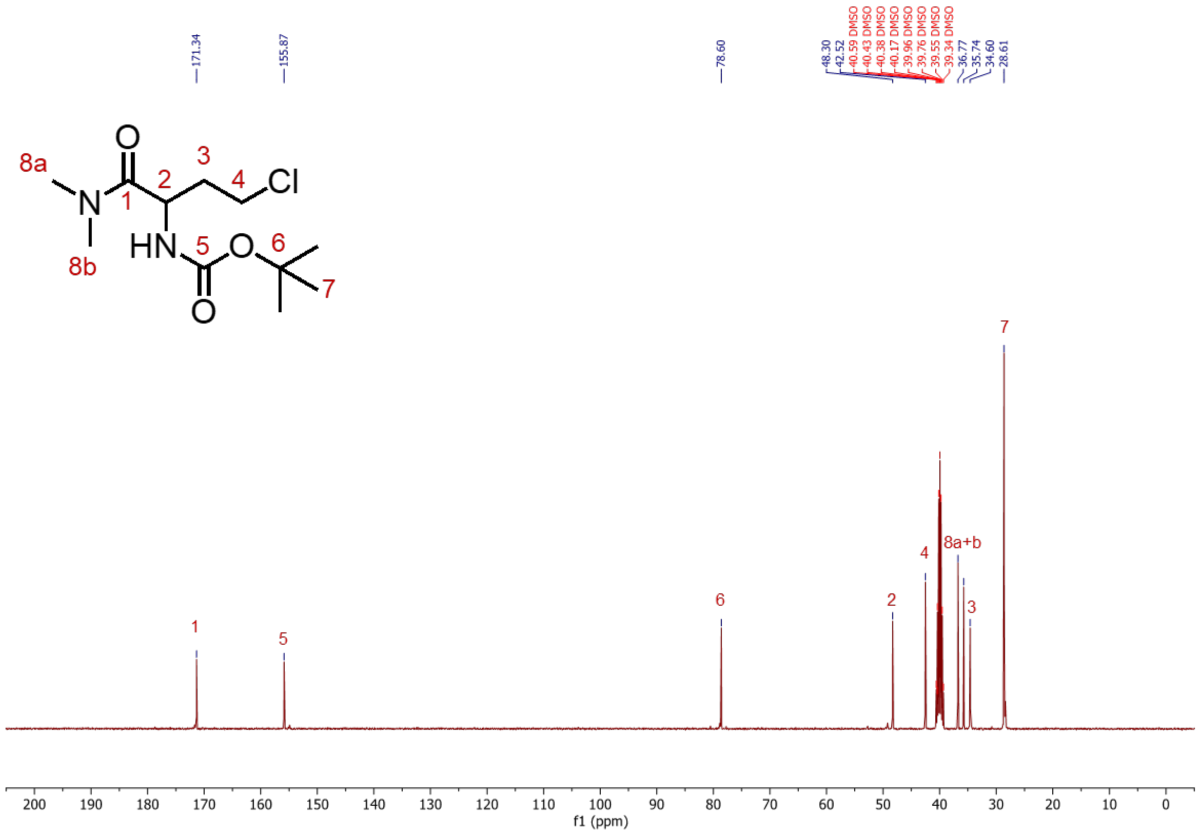

Compound **14** ^{1}H NMR

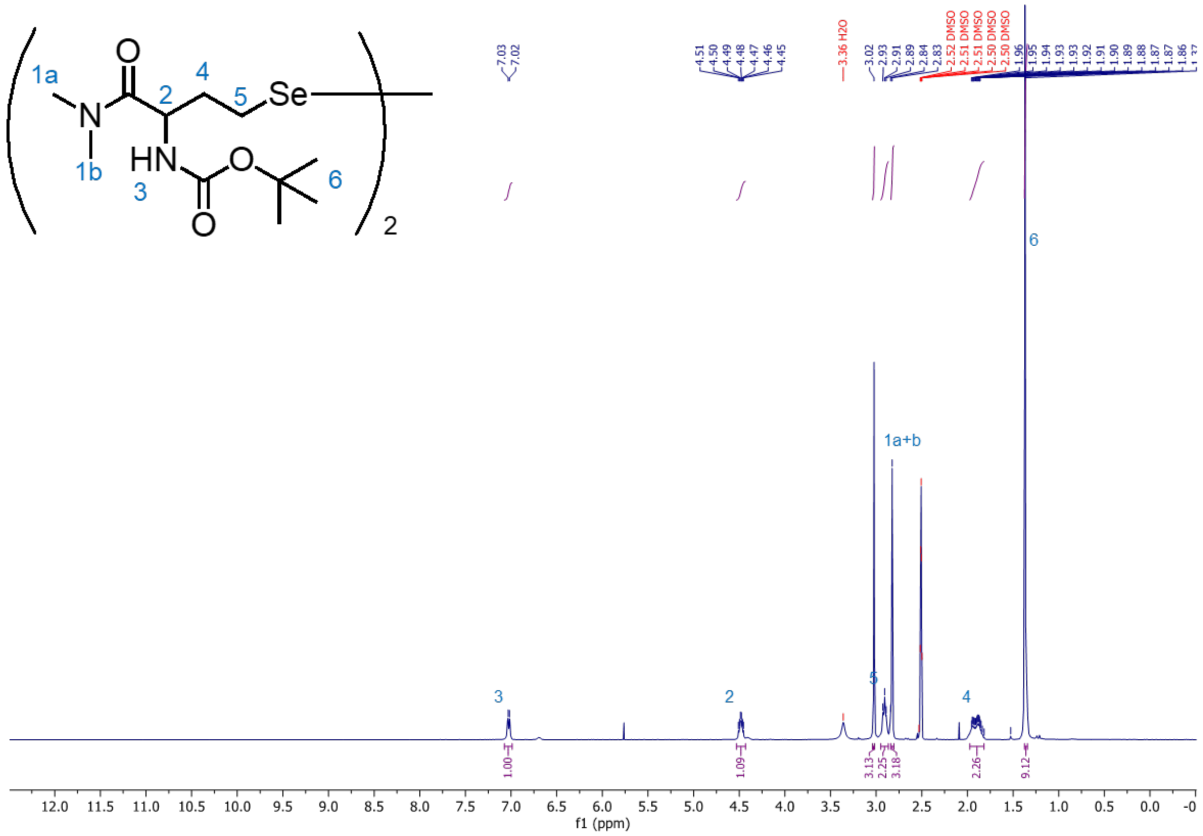

Compound **14** ^{13}C NMR

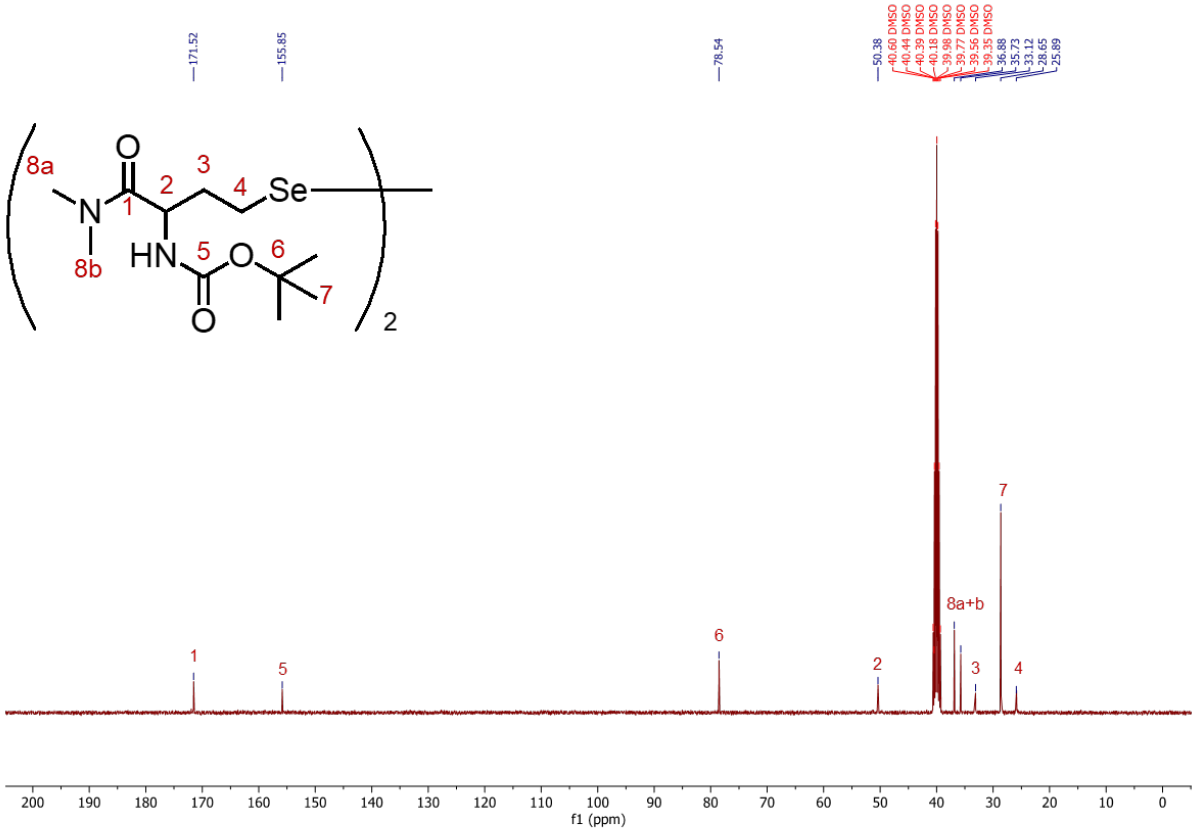

Compound **15** ^{1}H NMR

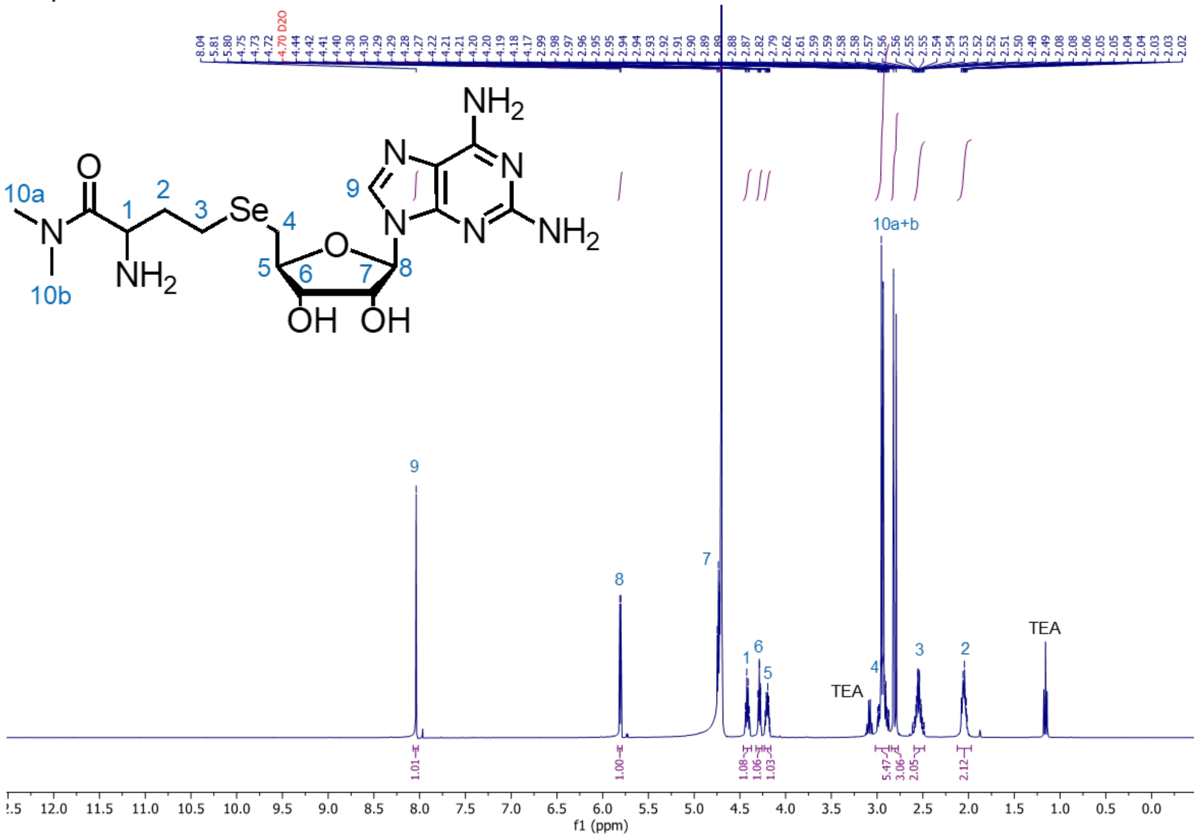

Compound **15** ^{13}C NMR

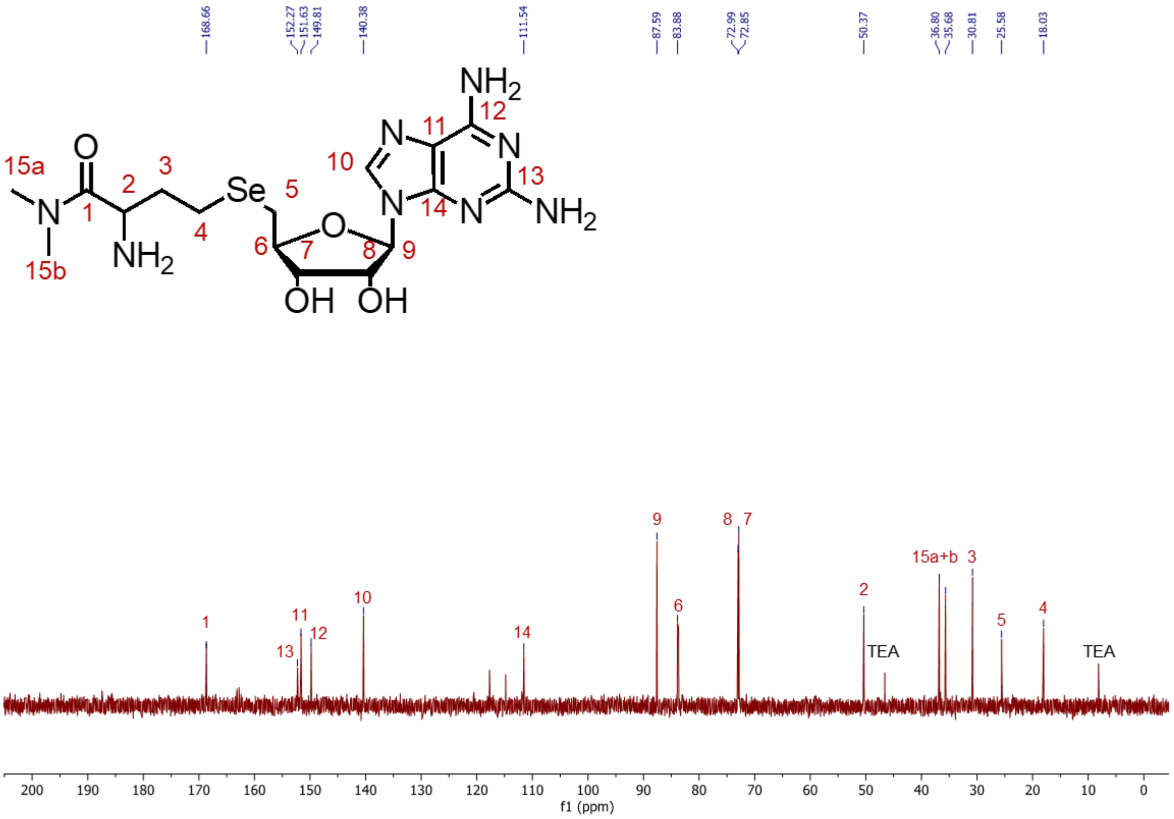

Compound **16** ^{1}H NMR

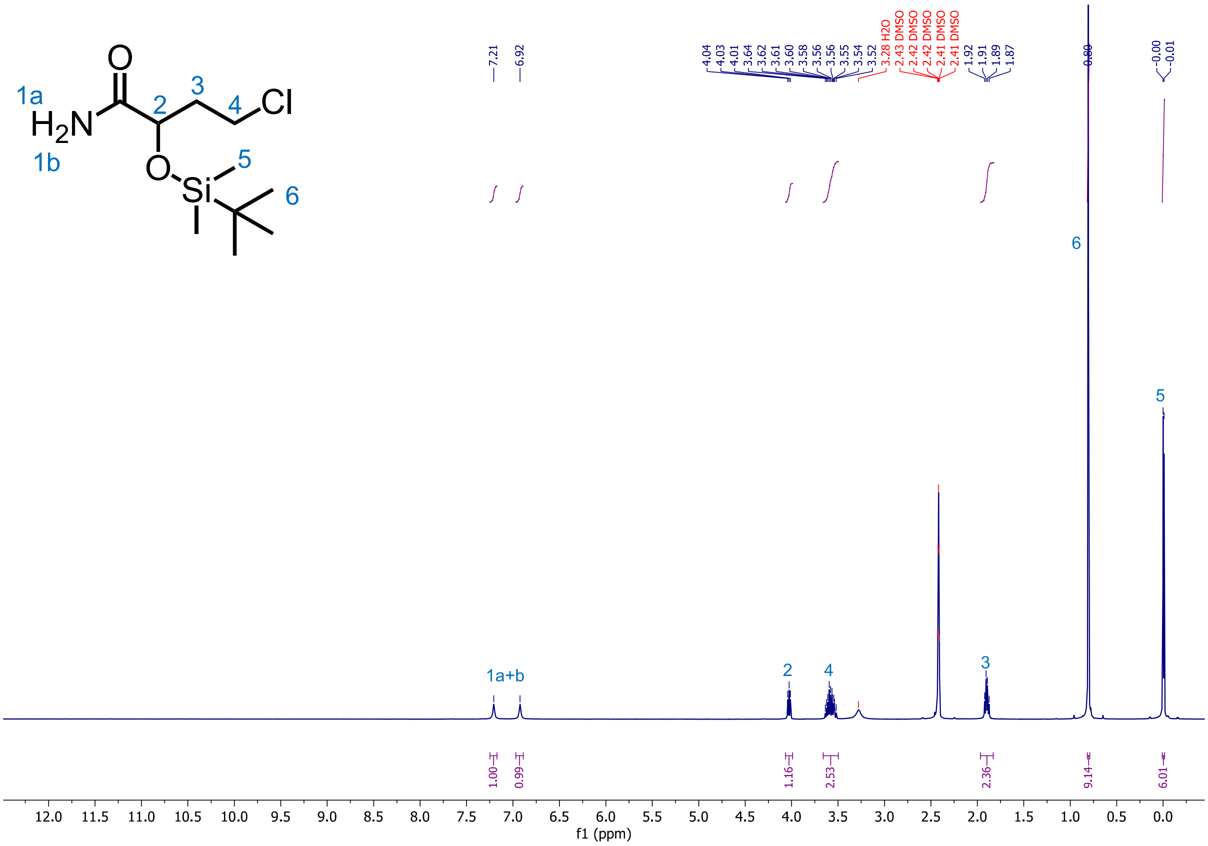

Compound **16** ^{13}C NMR

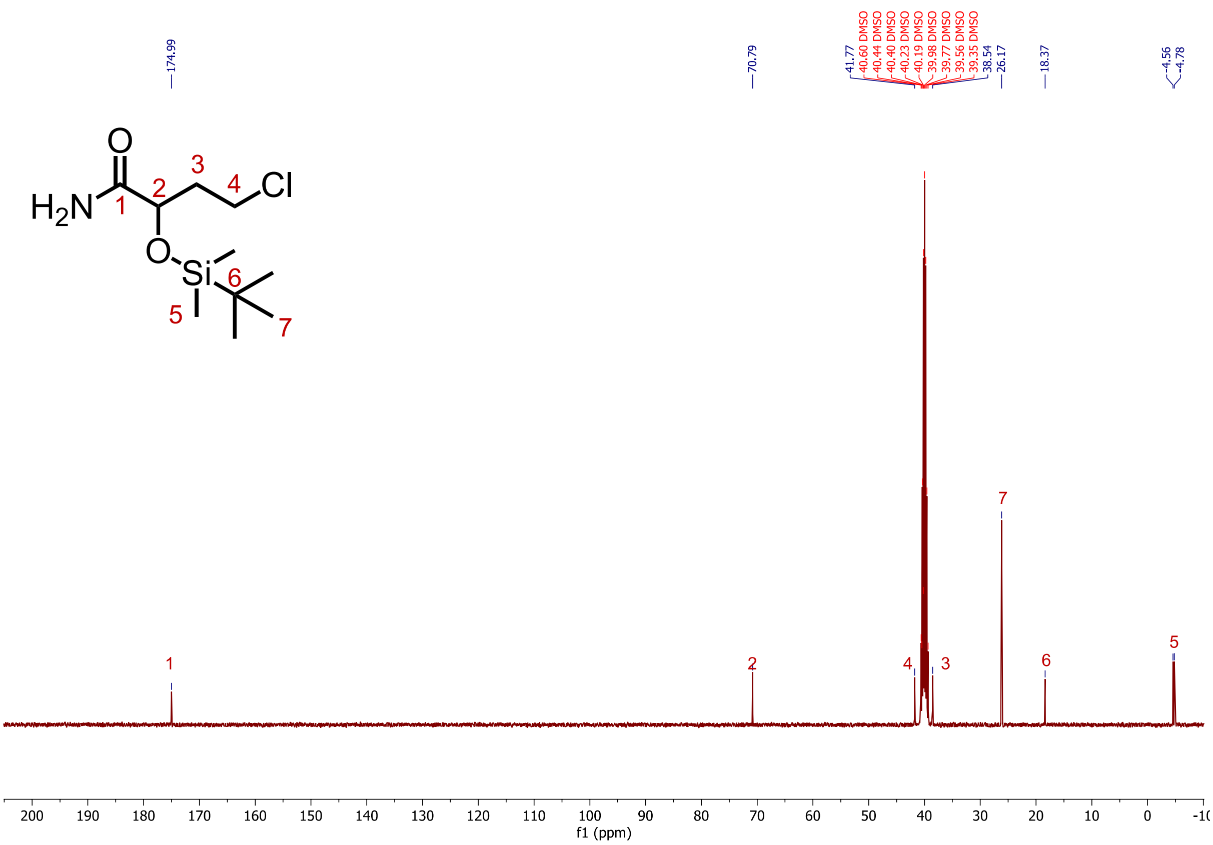

Compound **17** ^{1}H NMR

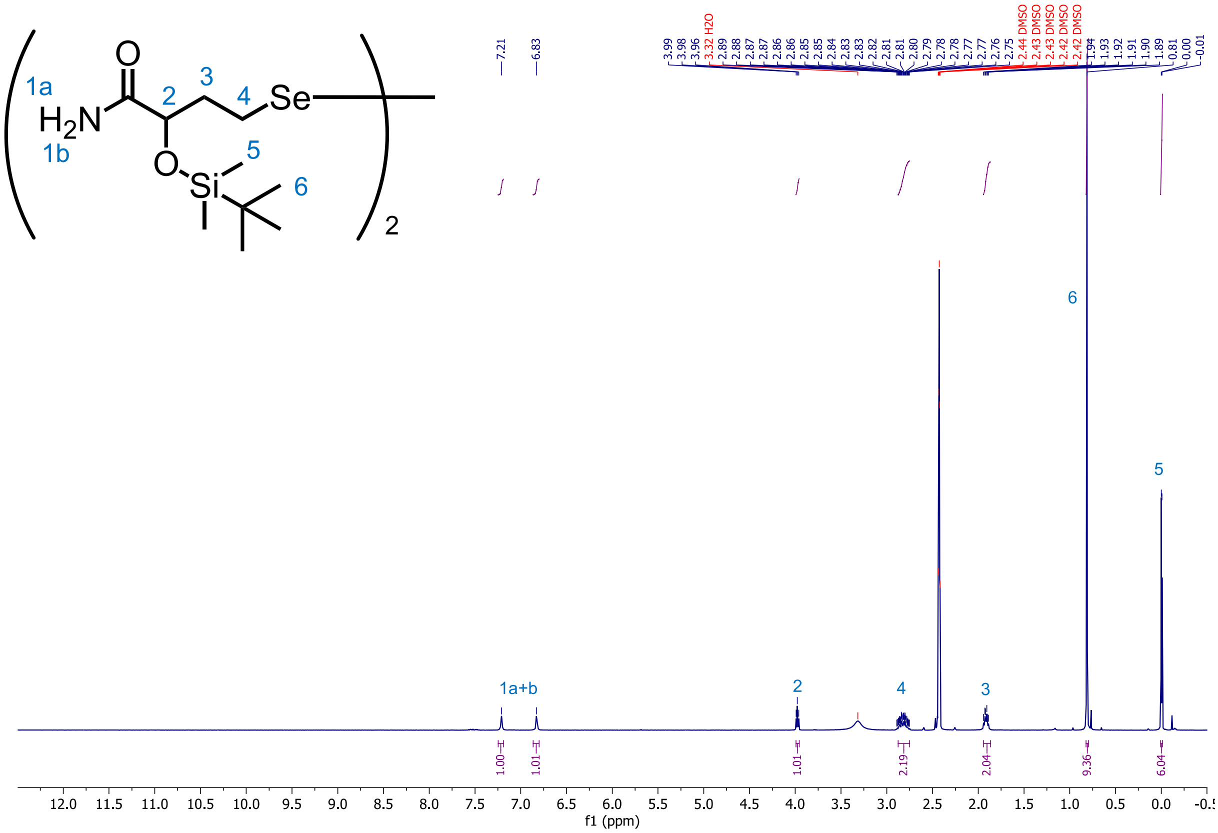

Compound **17** ^{13}C NMR

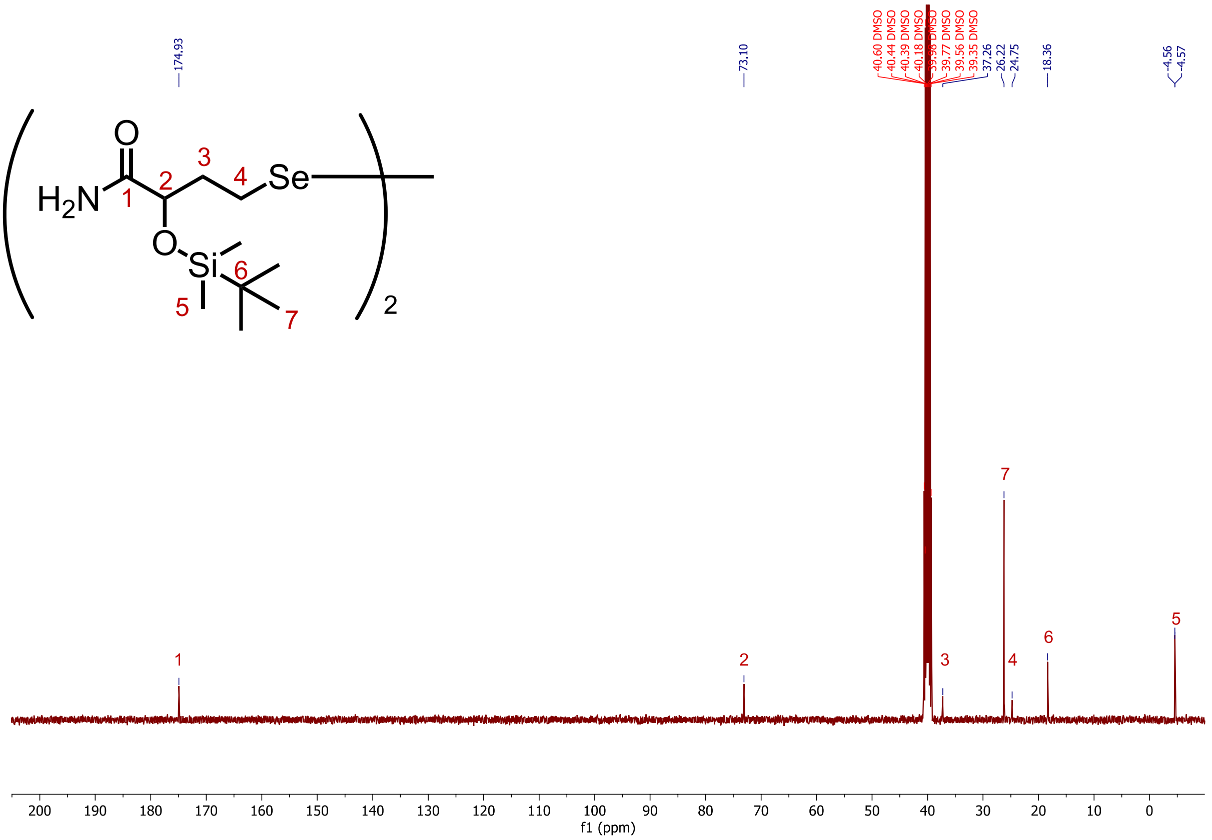

Compound **18** ^{1}H NMR

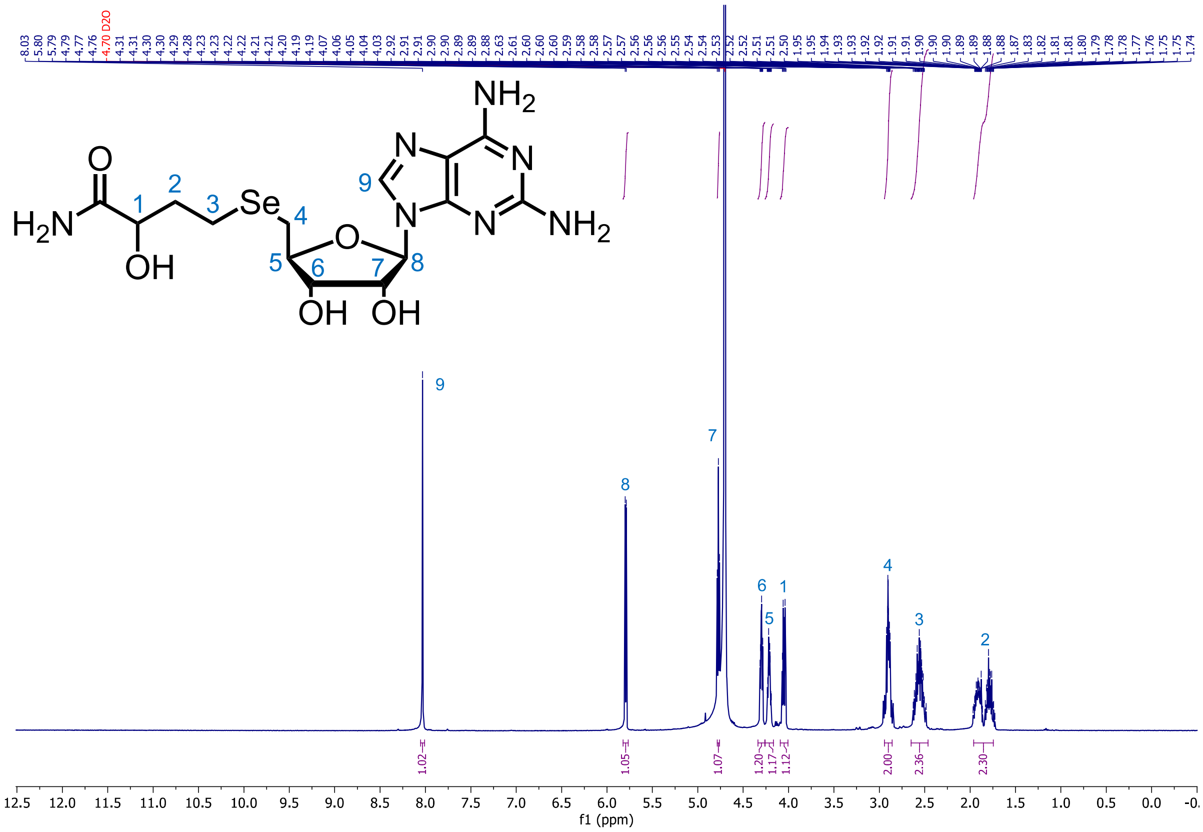

Compound **18** ^{13}C NMR

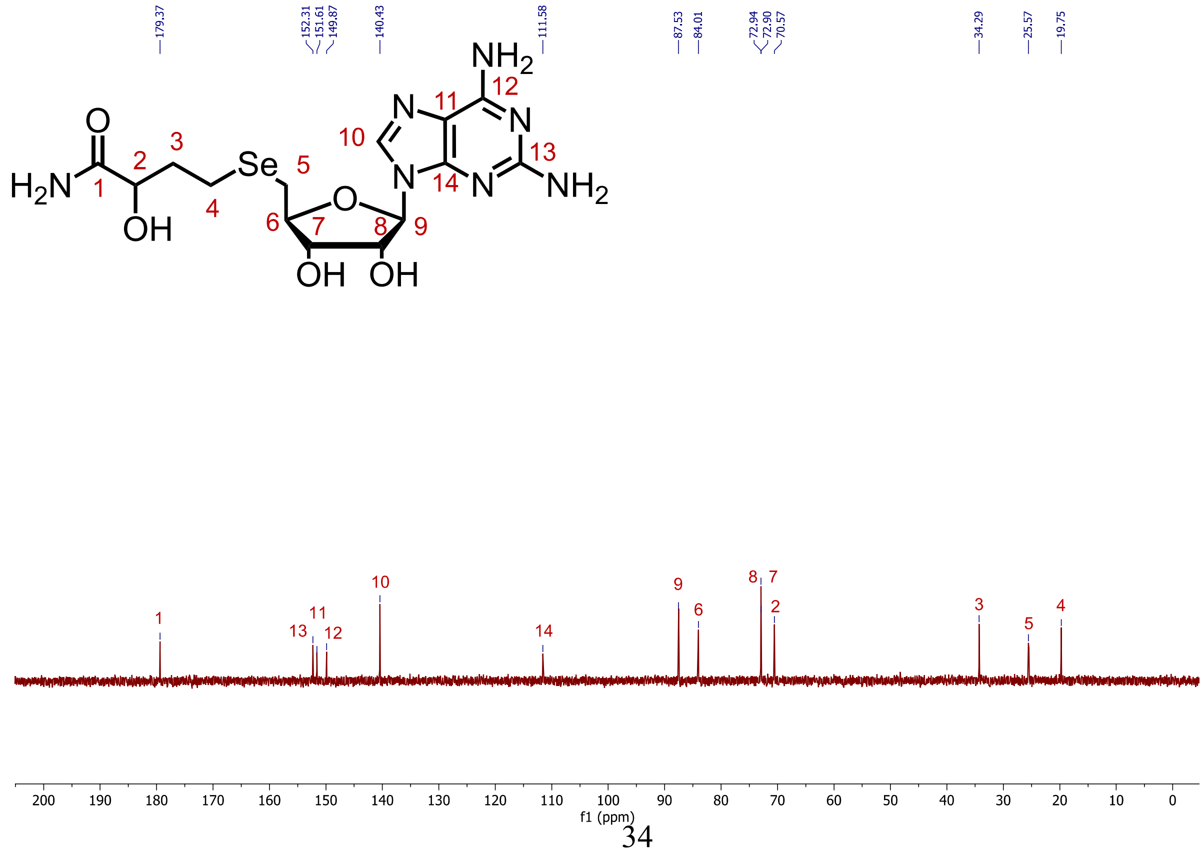

HR-ESI mass spectra

ProSeDMA (5)

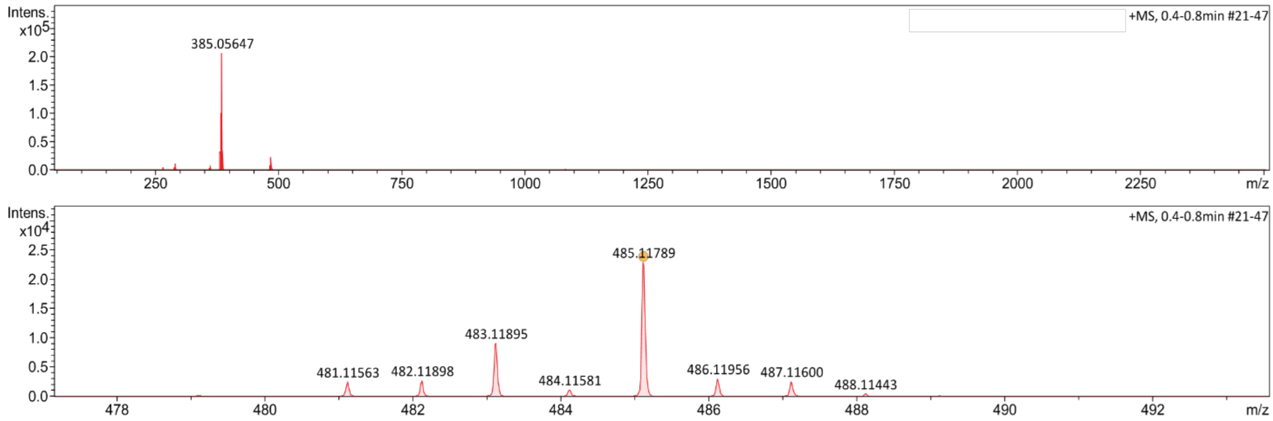

MeSeDMA (6)

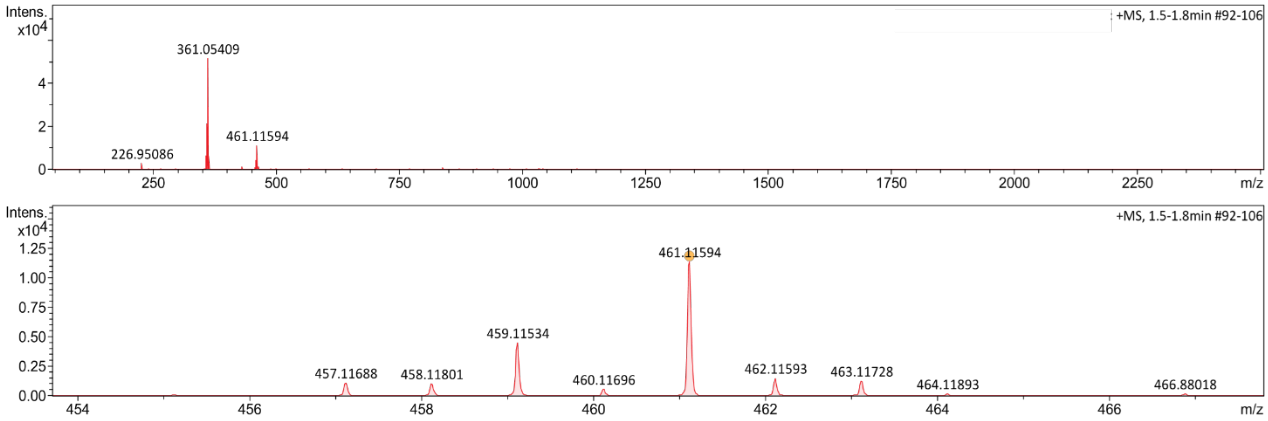

AllSeDMA (7)

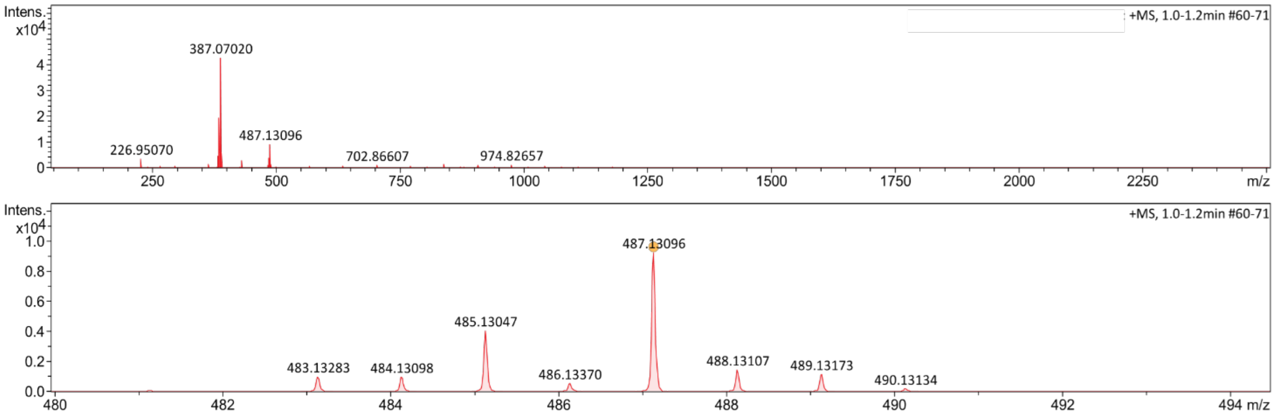

ProSeAM NMe$_2$ (8)

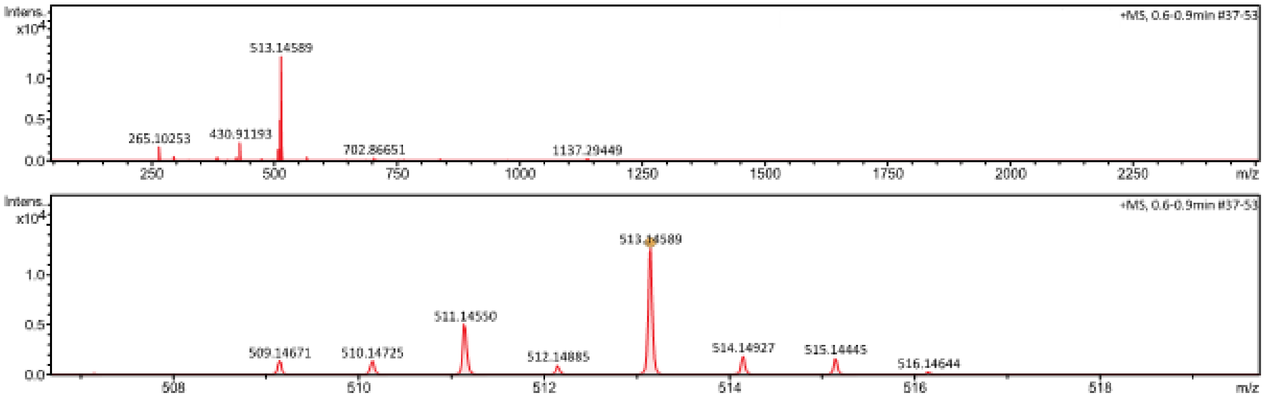

ProSeAM OH (9)

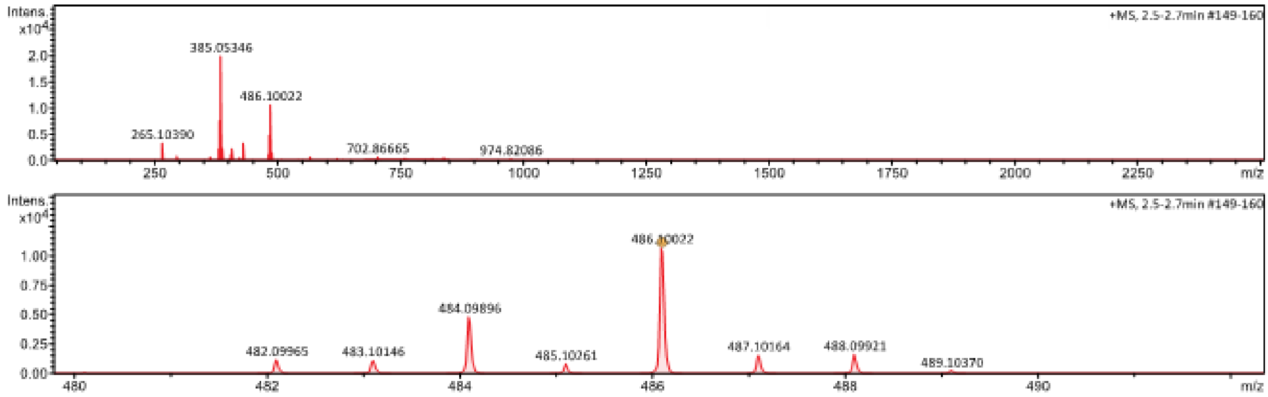

SDM

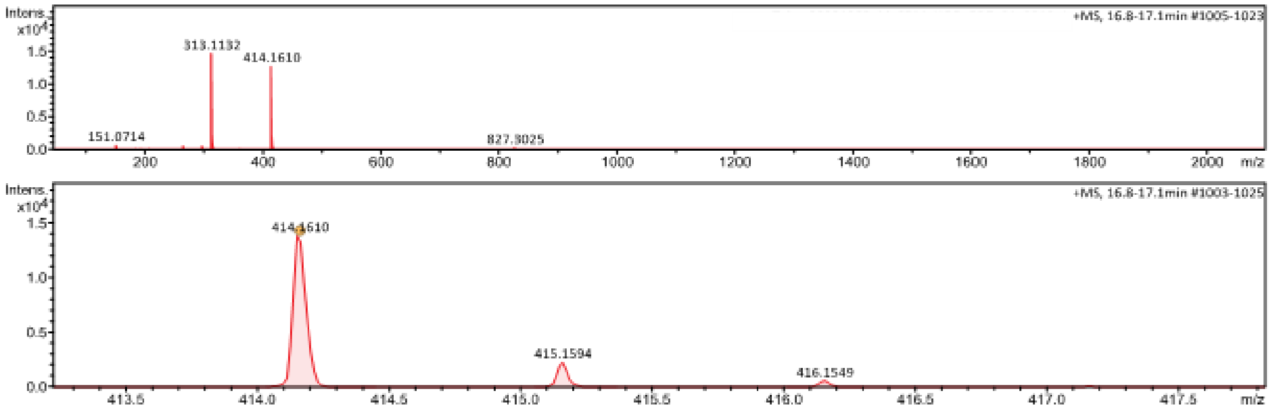

HPLC chromatograms

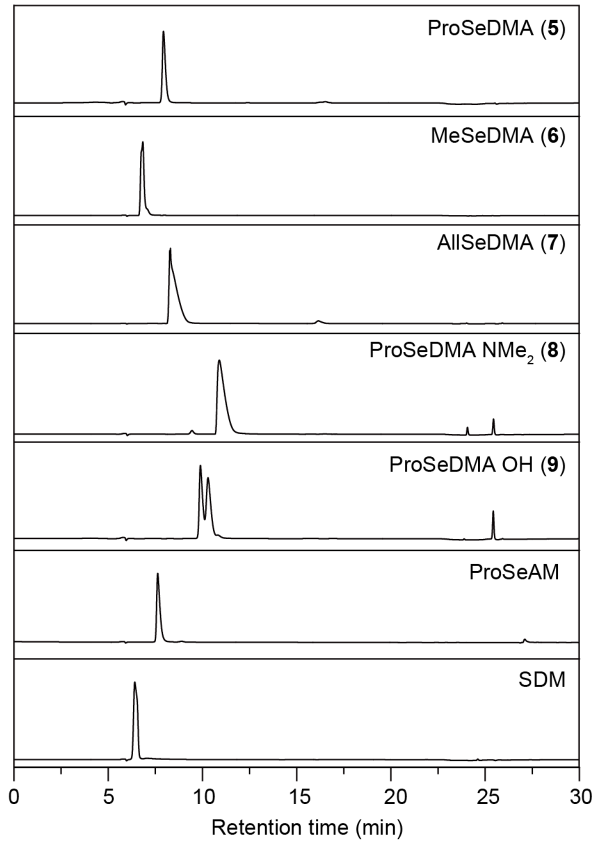

Conditions for HPLC analysis of all synthesized cofactors: RP-HPLC (NUCLEOSIL 100-5 C18 column (5 µm, 125 x 4 mm). Linear gradient B conc. 5% - 7% (0 min to 15 min), 7% - 70% (15min to 30 min); solvent A was H_2O + 0.1% TFA; solvent B was MeCN + 0.1% TFA; flow rate was 0.7 mL/min at 30°C with UV detection at 260 nm. Samples were injected from a 30 µM solution in 0.1% TFA in H_2O.

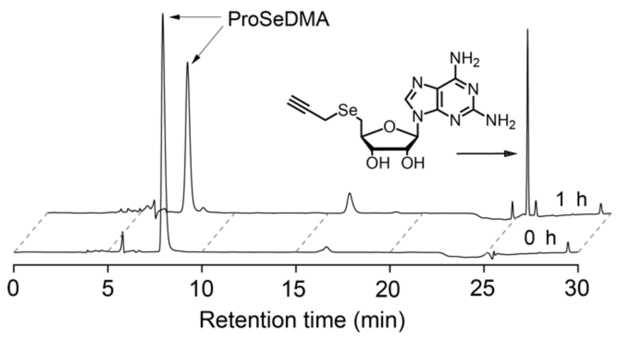

Stability assay of ProSeDMA (5) in cell lysate. 1 mM cofactor was incubated in cell lysate which was prepared from 10^6 HEK293T cells by RIPA buffer (150 mM NaCl, 1.0% IGEPAL® CA-630, 0.5% sodium deoxycholate, 0.1% SDS, 50 mM Tris, pH 7.5). Aliquots were analyzed by RP-HPLC, monitored at 260 nm.

References

(1) Weitz, I. S., Pellegrini, M., Mierke, D. F. & Chorev, M. Synthesis of a Trisubstituted 1,4-Diazepin-3-one-Based Dipeptidomimetic as a Novel Molecular Scaffold. *J. Org. Chem.* **62**, 2527–2534 (1997).

(2) Suzuki, K., & Takayama, H. First asymmetric total syntheses of (-)-subincanadines a and B, skeletally rearranged pentacyclic monoterpenoid indole alkaloids in aspidospermasubincanum. *Org. Lett.* **8**, 4605–4608 (2006).

(3) Chen, Y. K., Co, E. W., Guntupalli, P., Lawson, J. D., Notz, W. R. L., Stafford, J. A. & Ton-Nu, H.-T. Oxime derivatives as HSP90 inhibitors and their preparation, pharmaceutical compositions and use in the treatment of diseases. *WO2009097578A1*, (2009)

(4) Jiang, H., Congleton, J., Liu, Q., Merchant, P., Malavasi, F., Lee, H. C., Hao, Q., Yen, A. & Lin, H. Mechanism-based small molecule probes for labeling CD38 on live cells. *J. Am. Chem. Soc.* **131**, 1658–1659 (2009).

(5) Atdjian, C., Coelho, D., Iannazzo, L., Ethève-Quelquejeu, M. & Braud, E. Synthesis of Triazole-Linked SAM-Adenosine Conjugates: Functionalization of Adenosine at N1 or N6 Position without Protecting Groups. *Molecules* **25**, 3241 (2020).

(6) Jawalekar, A. M., Op de Beeck, M., van Delft, F. L. & Madder, A. Synthesis and incorporation of a furan-modified adenosine building block for DNA interstrand crosslinking. *Chem. Commun.* **47**, 2796–2798 (2011).

(7) Bothwell, I. & Luo, M. Large-Scale, Protection-Free Synthesis of Se-Adenosyl-l-selenomethionine Analogues and Their Application as Cofactor Surrogates of Methyltransferases. *Org. Lett.* **16**, 3056–3059 (2014).

(8) McKean, I. J. W., Sadler, J. C., Cuetos, A., Frese, A., Humphreys, L. D., Grogan, G., Hoskisson, P. A., & Burley, G. A. S-Adenosyl Methionine Cofactor Modifications Enhance the Biocatalytic Repertoire of Small Molecule C-Alkylation. *Angew. Chem. Int. Ed.* **58**, 17583–17588 (2019).
