# Supplementary material for: A SAM analogue-utilizing ribozyme for site-specific RNA alkylation in living cells
Source: Nat Chem. 2023 Sep 4;15(11):1523–31. doi: 10.1038/s41557-023-01320-z (PMC10624628; doi:10.1038/s41557-023-01320-z)
Supplement: Supplementary file 2 — Reporting Summary [file 41557_2023_1320_MOESM2_ESM.pdf]

nature portfolio

Corresponding author(s): Claudia Höbartner

Last updated by author(s): Jun 16, 2023

Reporting Summary

Nature Portfolio wishes to improve the reproducibility of the work that we publish. This form provides structure for consistency and transparency in reporting. For further information on Nature Portfolio policies, see our Editorial Policies and the Editorial Policy Checklist.

Statistics

For all statistical analyses, confirm that the following items are present in the figure legend, table legend, main text, or Methods section.

n/a	Confirmed	
☐	☒	The exact sample size (n) for each experimental group/condition, given as a discrete number and unit of measurement
☒	☐	A statement on whether measurements were taken from distinct samples or whether the same sample was measured repeatedly
☒	☐	The statistical test(s) used AND whether they are one- or two-sided<br>*Only common tests should be described solely by name; describe more complex techniques in the Methods section.*
☒	☐	A description of all covariates tested
☒	☐	A description of any assumptions or corrections, such as tests of normality and adjustment for multiple comparisons
☐	☒	A full description of the statistical parameters including central tendency (e.g. means) or other basic estimates (e.g. regression coefficient) AND variation (e.g. standard deviation) or associated estimates of uncertainty (e.g. confidence intervals)
☒	☐	For null hypothesis testing, the test statistic (e.g. F, t, r) with confidence intervals, effect sizes, degrees of freedom and P value noted<br>*Give P values as exact values whenever suitable.*
☒	☐	For Bayesian analysis, information on the choice of priors and Markov chain Monte Carlo settings
☒	☐	For hierarchical and complex designs, identification of the appropriate level for tests and full reporting of outcomes
☒	☐	Estimates of effect sizes (e.g. Cohen's d, Pearson's r), indicating how they were calculated

Our web collection on statistics for biologists contains articles on many of the points above.

Software and code

Policy information about availability of computer code

Data collection	gel images with fluorescently labeled RNA: BioRad Chemidoc; BioRad fluorescent cell imager; Anion exchange HPLC: Dionex DNAPAc column on ÄKTA micro; RP-HPLC: Nucleosil column on Jasco HPLC; UV detection at 260 nm; LC-MS: ThermoUltimate3000 HPLC with Synergi RP C18 column. HR-ESI-MS: Bruker MicroTof-QIII.
Data analysis	Origin (2019), Compass Data Analysis software 4.2 (Bruker Daltonics)

For manuscripts utilizing custom algorithms or software that are central to the research but not yet described in published literature, software must be made available to editors and reviewers. We strongly encourage code deposition in a community repository (e.g. GitHub). See the Nature Portfolio guidelines for submitting code & software for further information.

Data

Policy information about availability of data

All manuscripts must include a data availability statement. This statement should provide the following information, where applicable:

- Accession codes, unique identifiers, or web links for publicly available datasets
- A description of any restrictions on data availability
- For clinical datasets or third party data, please ensure that the statement adheres to our policy

The data generated and analysed during this study are included in this published article, in the Supplementary Information and in the Source Data files.

Human research participants

Policy information about studies involving human research participants and Sex and Gender in Research.

Reporting on sex and gender	not applicable
Population characteristics	not applicable
Recruitment	not applicable
Ethics oversight	not applicable

Note that full information on the approval of the study protocol must also be provided in the manuscript.

Field-specific reporting

Please select the one below that is the best fit for your research. If you are not sure, read the appropriate sections before making your selection.

☒ Life sciences ☐ Behavioural & social sciences ☐ Ecological, evolutionary & environmental sciences

For a reference copy of the document with all sections, see nature.com/documents/nr-reporting-summary-flat.pdf

Life sciences study design

All studies must disclose on these points even when the disclosure is negative.

Sample size	Samples sizes for experiments with transfected HEK293T cells: 100,000 cells were seeded in 12-well culture plate and grown for 24 h prior to the respective experiments, as described in material and methods. For cell viability assays, 24-well culture plates were used.
Data exclusions	No data was excluded from the analyses.
Replication	For all biochemical assays described in the manuscript, the experiments were performed in two or three independent replicates (described in material and methods), following the common standards in the field of ribozyme characterization. All attempts of replication were successful.
Randomization	Randomization was not required, since no organisms/animal or human subjects were used in this study, and because ribozyme characterization was done by measuring physical values, which are not influenced by the observer.
Blinding	blinding was not relevant to the study, because there was no manual scoring involved.

Reporting for specific materials, systems and methods

We require information from authors about some types of materials, experimental systems and methods used in many studies. Here, indicate whether each material, system or method listed is relevant to your study. If you are not sure if a list item applies to your research, read the appropriate section before selecting a response.

Materials & experimental systems

n/a	Involved in the study	
☒	☐	Antibodies
☐	☒	Eukaryotic cell lines
☒	☐	Palaeontology and archaeology
☒	☐	Animals and other organisms
☒	☐	Clinical data
☒	☐	Dual use research of concern

Methods

n/a	Involved in the study	
☒	☐	ChIP-seq
☒	☐	Flow cytometry
☒	☐	MRI-based neuroimaging

Eukaryotic cell lines

Policy information about cell lines and Sex and Gender in Research

Cell line source(s)	Human embryonic kidney (HEK) 293T cell was purchased from the American Type Culture Collection (ATCC).
Authentication	The cell lines was authenticated by vendor by STR profiling.

Mycoplasma contamination	The cell was tested negative for mycoplasma contamination before passaging.
Commonly misidentified lines (See ICLAC register)	No commonly misidentified cell lines were used.
