# Supplementary figures and images for: A SAM analogue-utilizing ribozyme for site-specific RNA alkylation in living cells
Source: Nat Chem. 2023 Sep 4;15(11):1523–31. doi: 10.1038/s41557-023-01320-z (PMC10624628; doi:10.1038/s41557-023-01320-z)

Fig. 2c

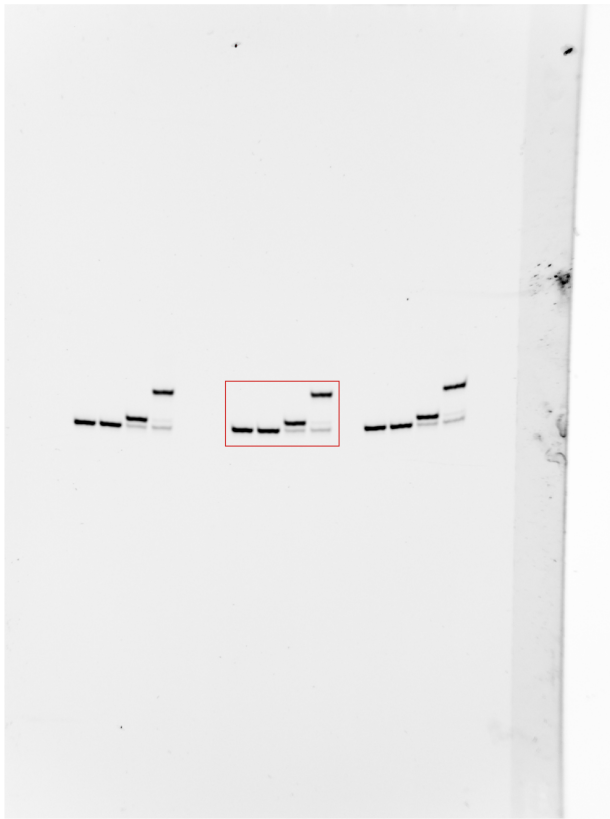

20 % dPAGE, 20×30 cm, 35W

Fig. 2d

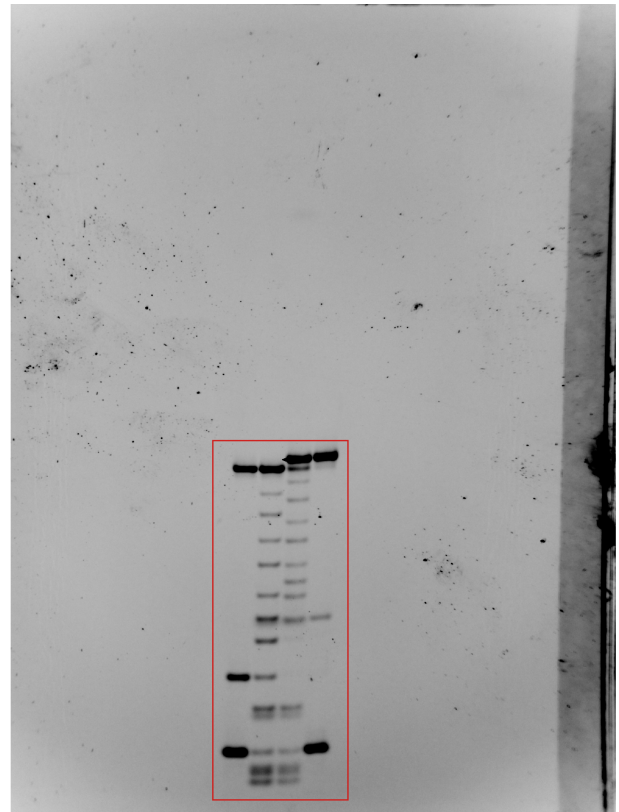

20 % dPAGE, 20×30 cm, 35W

Fig. 2e

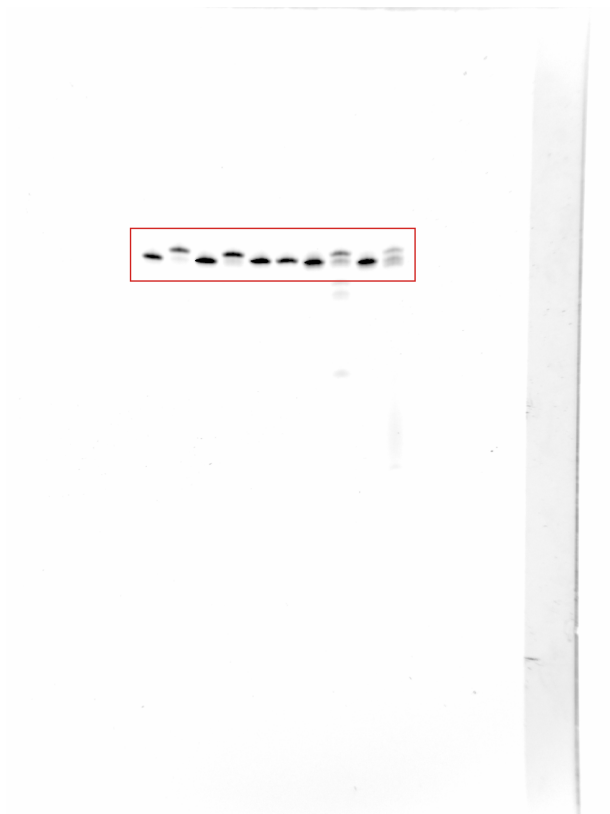

20 % dPAGE, 20×30 cm, 35W

Supplement: Supplementary file 3 — Unprocessed full size gels for Fig. 2c,e. [file 41557_2023_1320_MOESM3_ESM.pdf]

Fig. 4 d Cy5

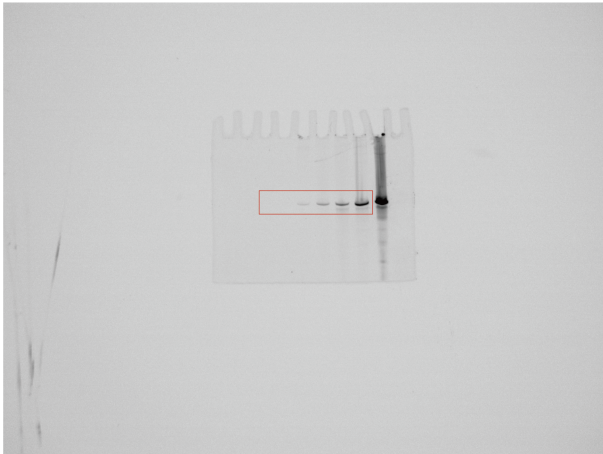

5 % dPAGE, 10×8 cm, 200 V

Fig. 4 d DFHBI

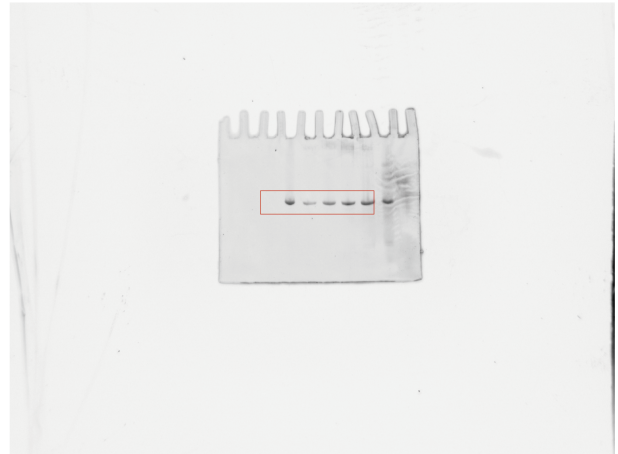

5 % dPAGE, 10×8 cm, 200 V

Fig. 4 d Sybr Gold

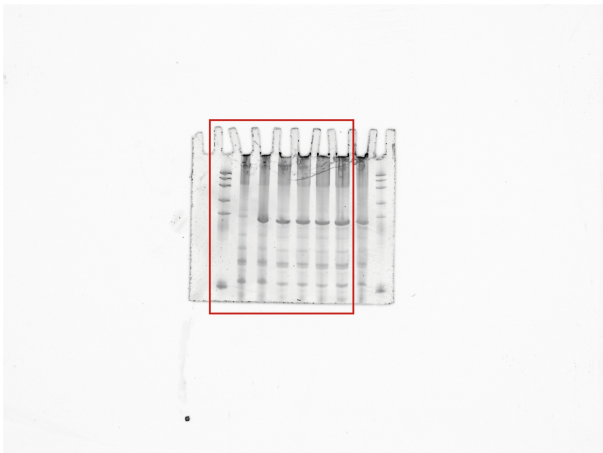

5 % dPAGE, 10×8 cm, 200 V

Supplement: Supplementary file 7 — Unprocessed full size gels for Fig. 4d. [file 41557_2023_1320_MOESM7_ESM.pdf]

ED Fig. 1b

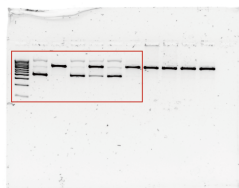

0.8 % agarose gel, 10×8 cm, 75 V

ED Fig. 1c

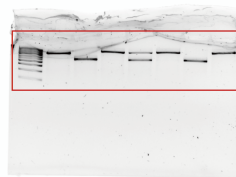

0.8 % agarose gel, 10×8 cm, 75 V

Supplement: Supplementary file 9 — Unprocessed gels for Extended Data Fig. 1b,c. [file 41557_2023_1320_MOESM9_ESM.pdf]
