# Supplementary material for: A SAM analogue-utilizing ribozyme for site-specific RNA alkylation in living cells
Source: Nat Chem. 2023 Sep 4;15(11):1523–31. doi: 10.1038/s41557-023-01320-z (PMC10624628; doi:10.1038/s41557-023-01320-z)
Supplement: Supplementary file 5 — Unprocessed full size gels for Fig. 3a,b,d,f. [file 41557_2023_1320_MOESM5_ESM.pdf]

Fig. 3a

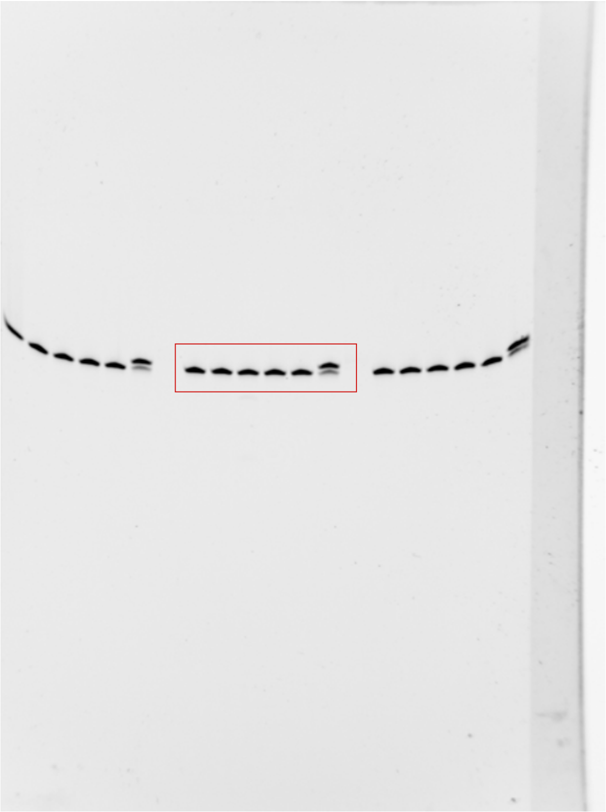

20 % dPAGE, 20×30 cm, 35W

Fig. 3b

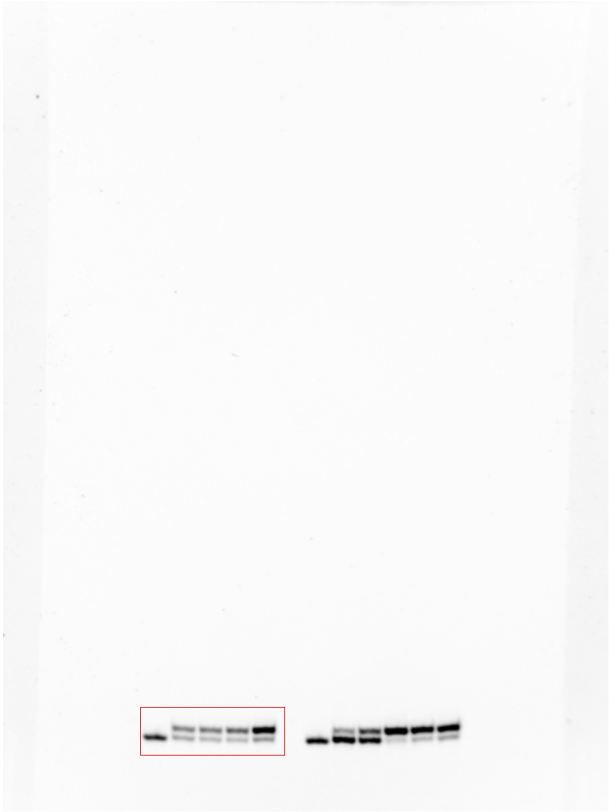

20 % dPAGE, 20×30 cm, 35W

Fig. 3c

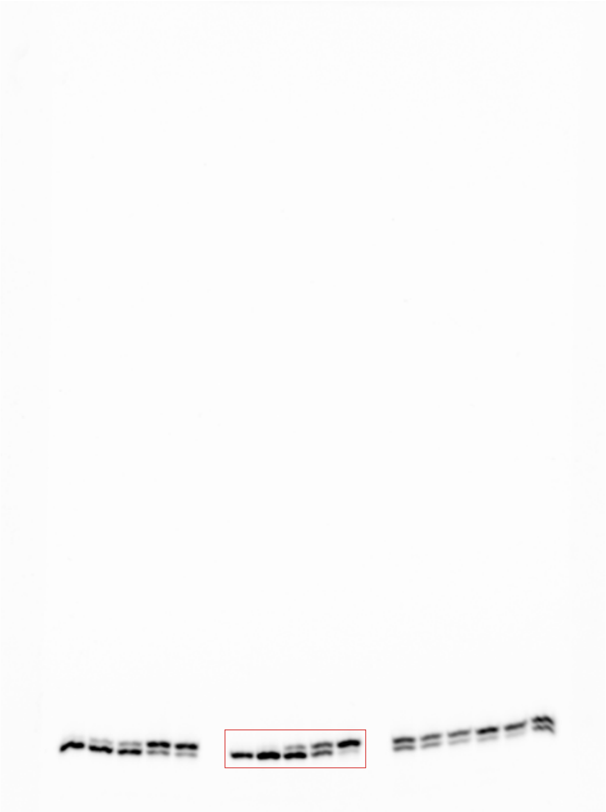

20 % dPAGE, 20×30 cm, 35W

Fig. 3d 1 μM

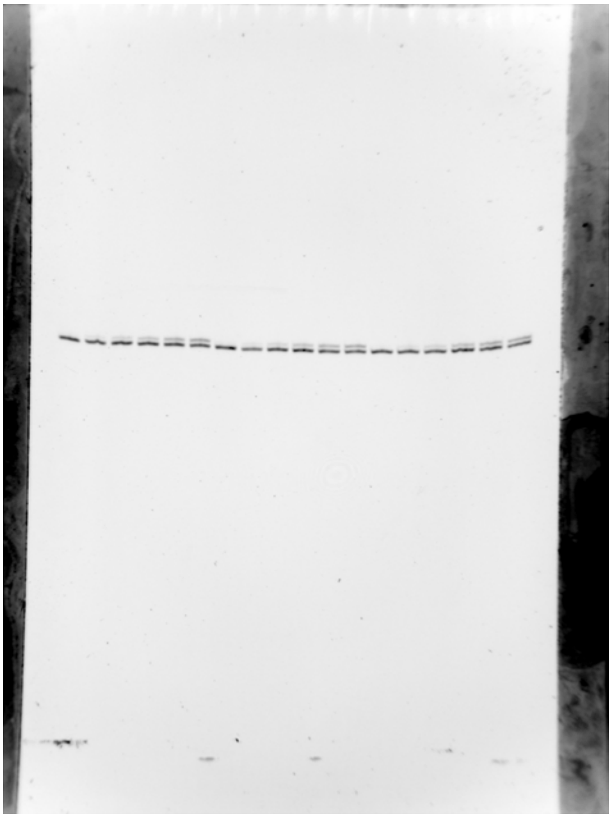

20 % dPAGE, 20×30 cm, 35W

Fig. 3d 2 μM, 3 μM, 4 μM

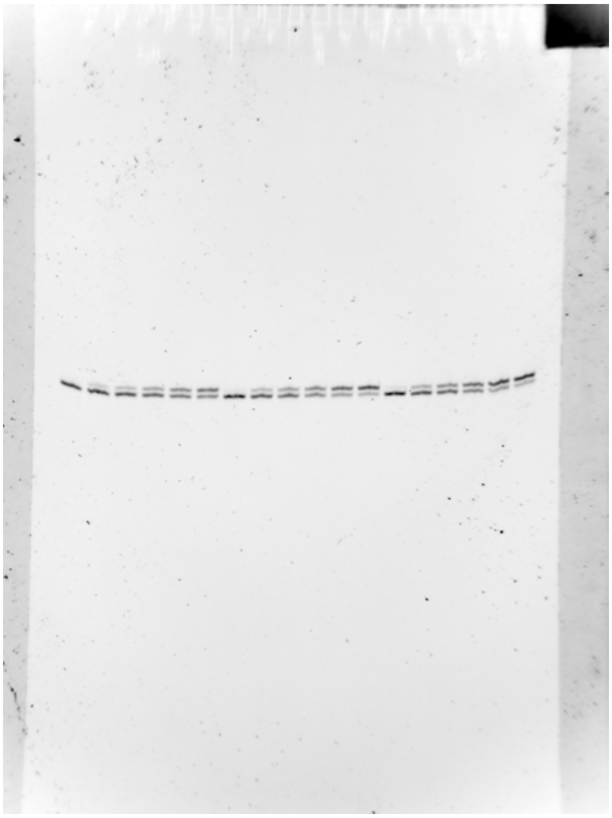

20 % dPAGE, 20×30 cm, 35W

Fig. 3d 5 μM

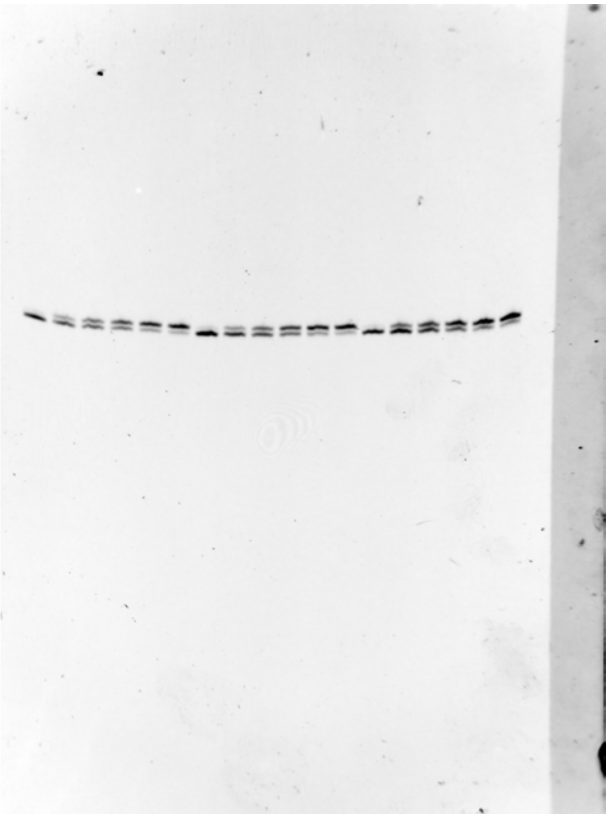

20 % dPAGE, 20×30 cm, 35W

Fig. 3d 20 μM

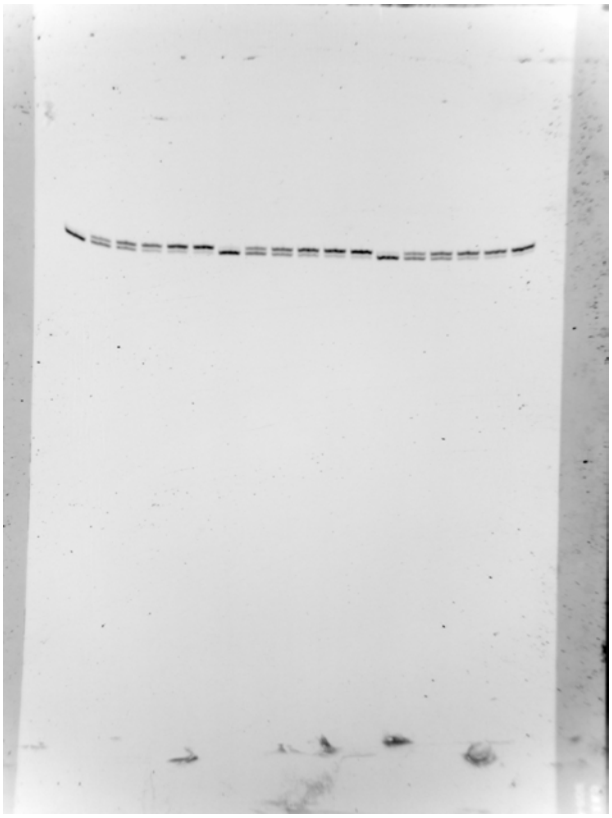

20 % dPAGE, 20×30 cm, 35W

Fig. 3d 50 μM

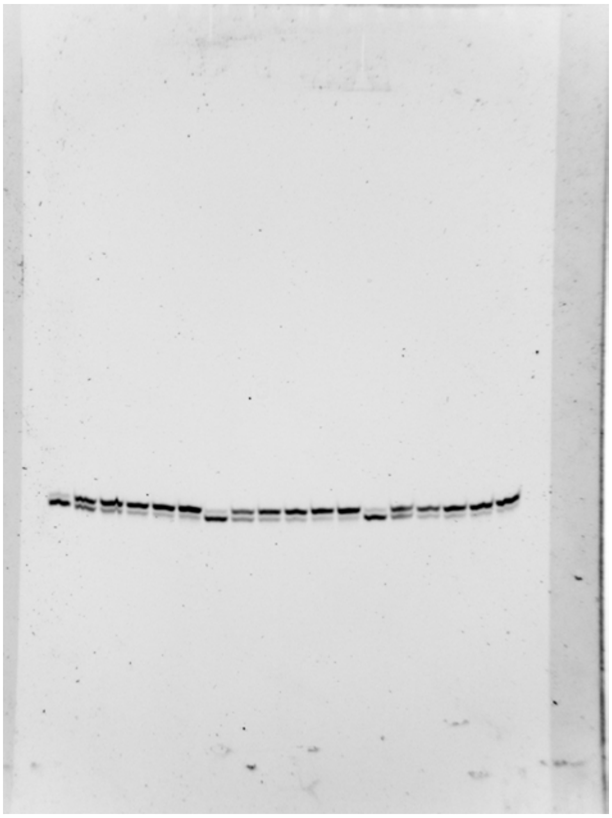

20 % dPAGE, 20×30 cm, 35W

Fig. 3e 10 μM

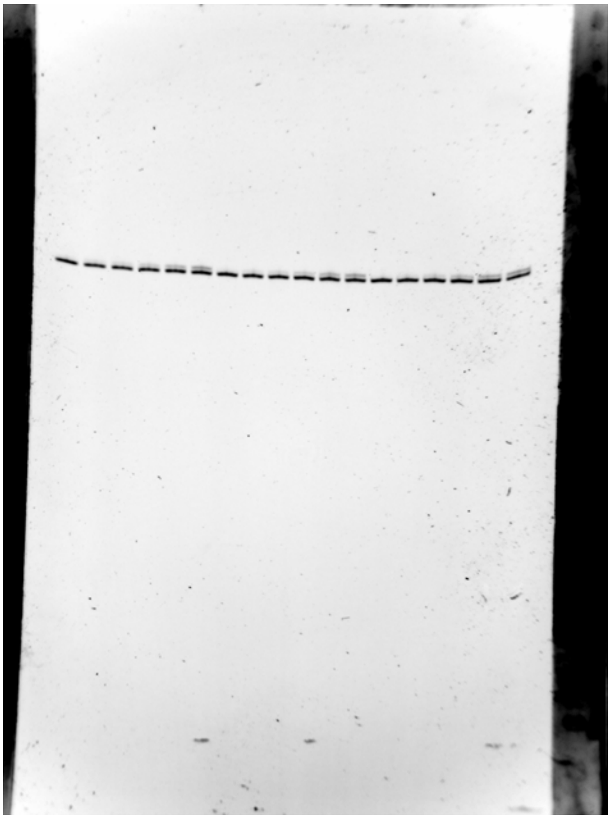

20 % dPAGE, 20×30 cm, 35W

Fig. 3e 20 μM

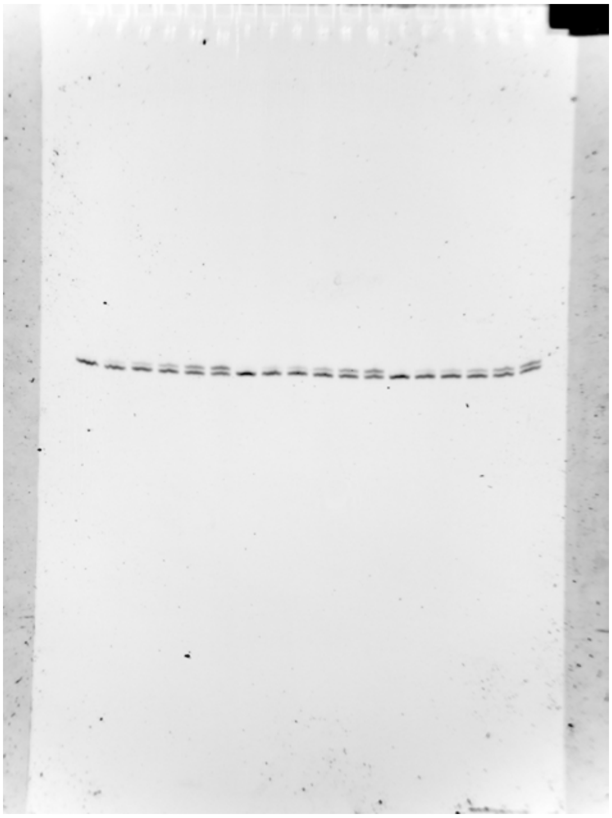

20 % dPAGE, 20×30 cm, 35W

Fig. 3e 30 μM

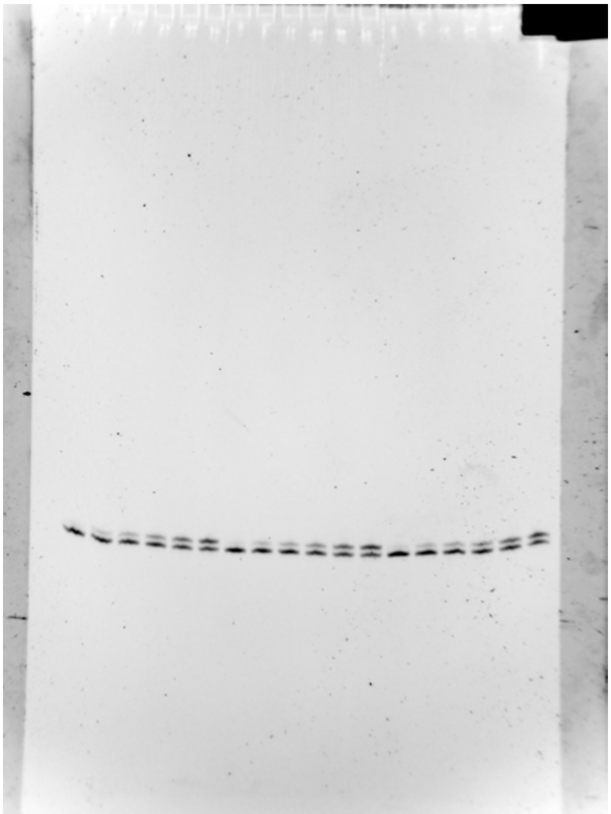

20 % dPAGE, 20×30 cm, 35W

Fig. 3e 40 μM

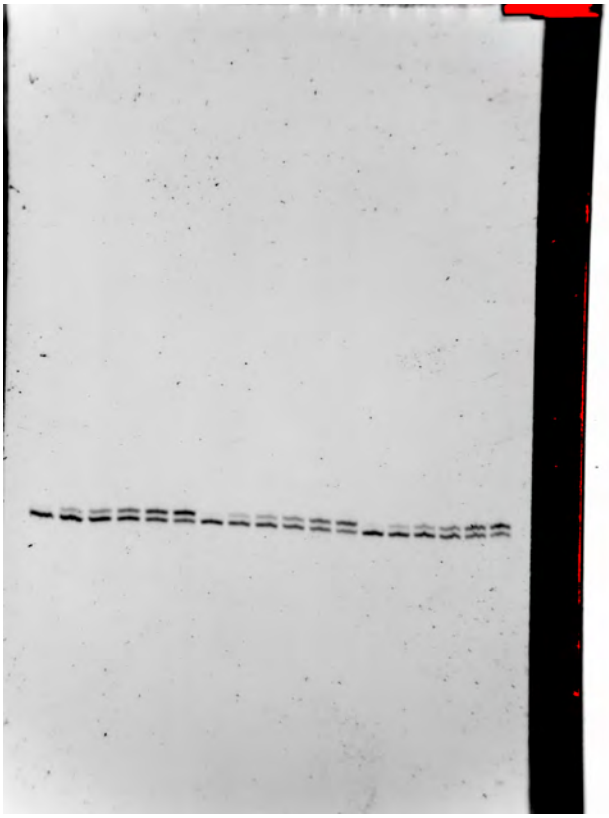

20 % dPAGE, 20×30 cm, 35W

Fig. 3e 50 μM

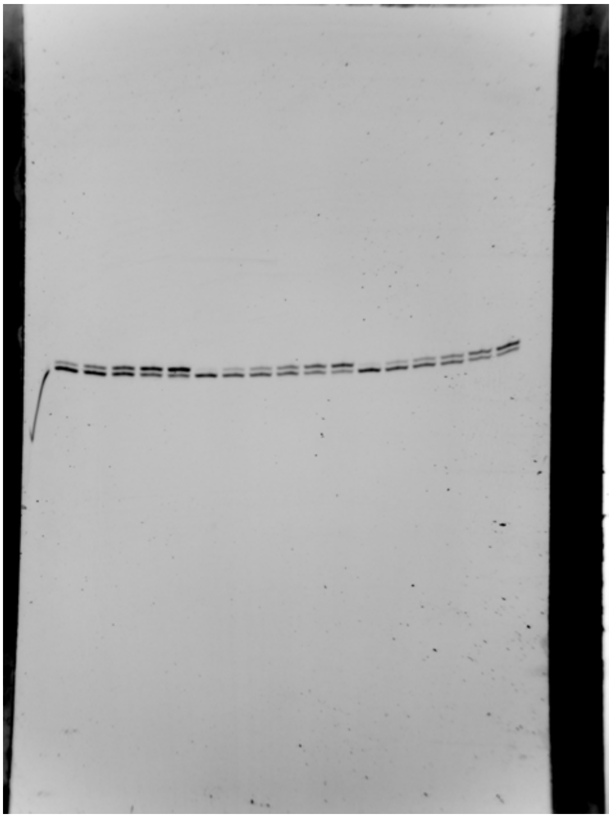

20 % dPAGE, 20×30 cm, 35W

Fig. 3e 100 μM

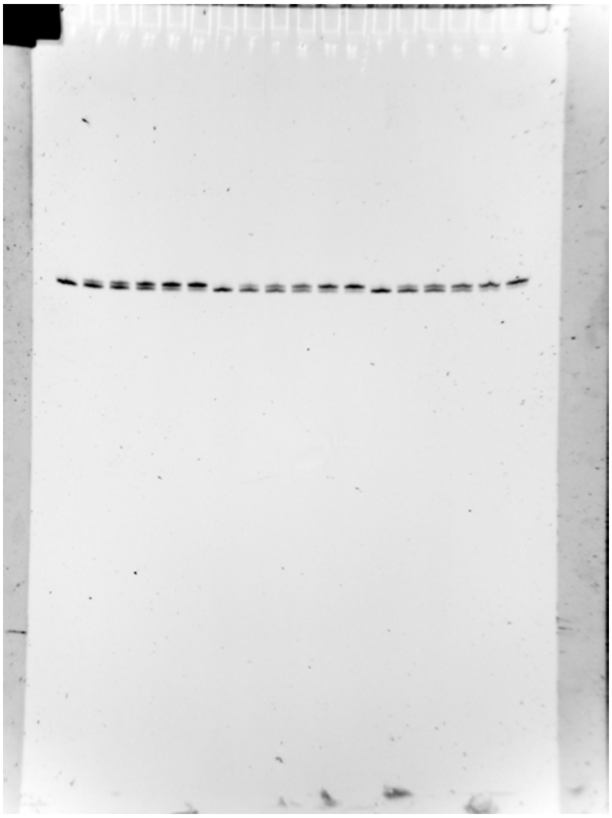

20 % dPAGE, 20×30 cm, 35W

Fig. 3e 150 μM

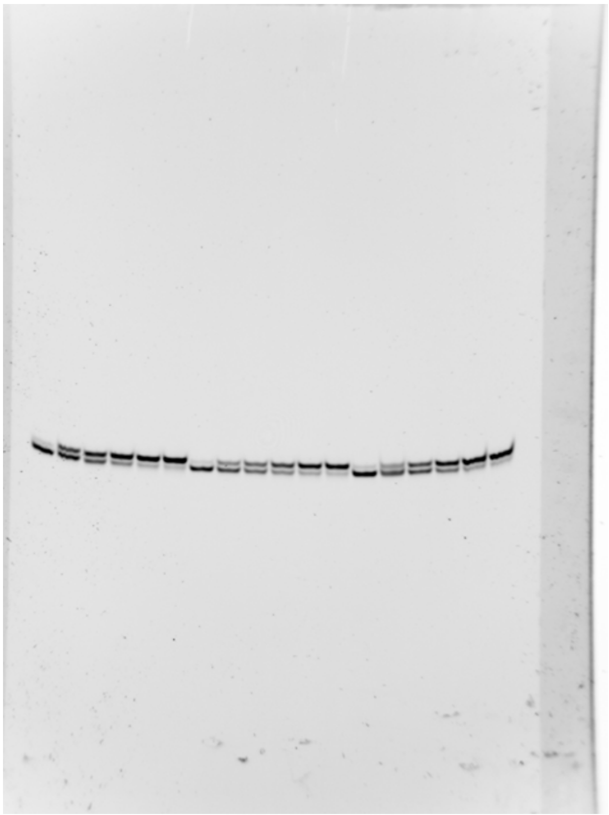

20 % dPAGE, 20×30 cm, 35W

Fig. 3f ProSeDMA

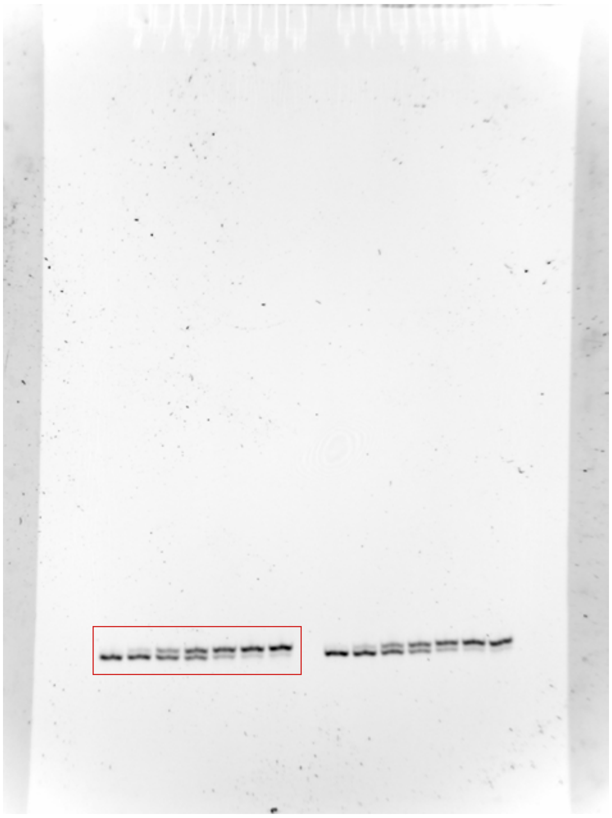

20 % dPAGE, 20×30 cm, 35W

Fig. 3f AllSeDMA

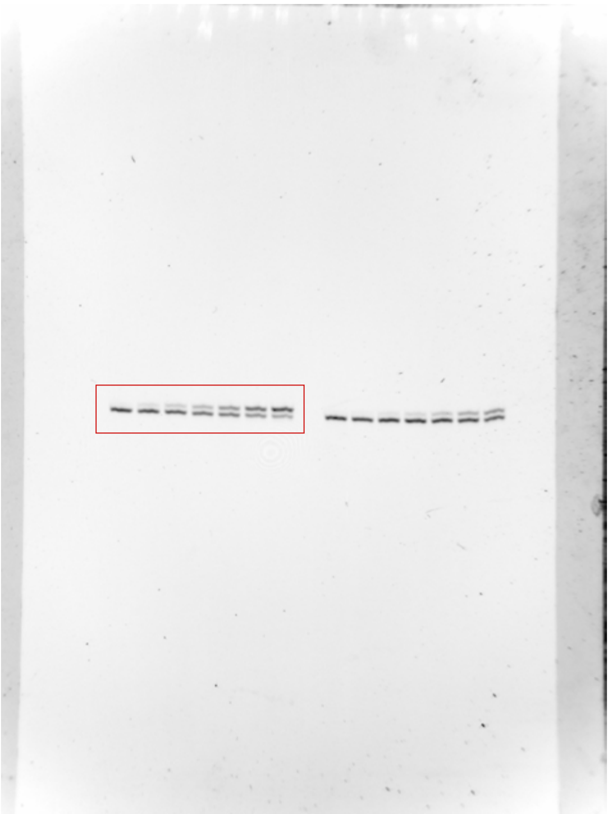

20 % dPAGE, 20×30 cm, 35W

Fig. 3f ProSeDMA OH

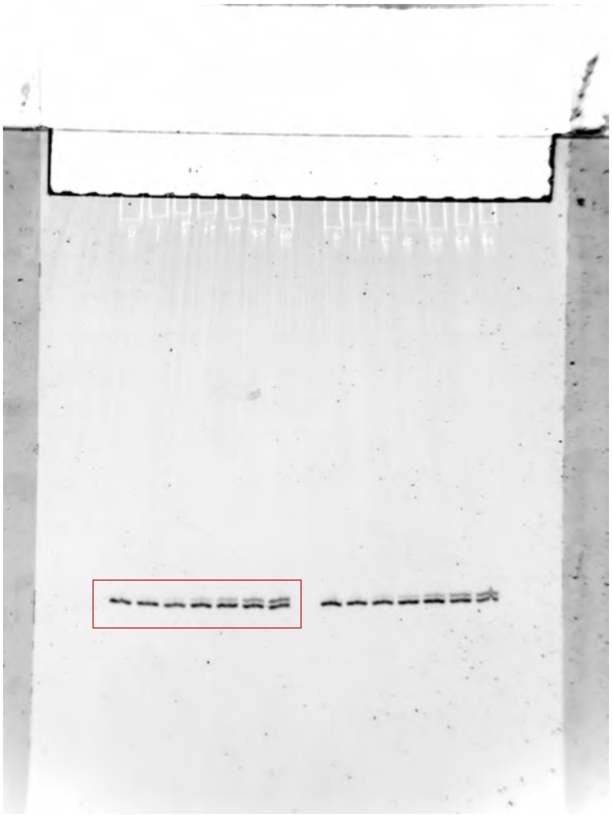

20 % dPAGE, 20×30 cm, 35W

Fig. 3f ProSeDMA NMe_2

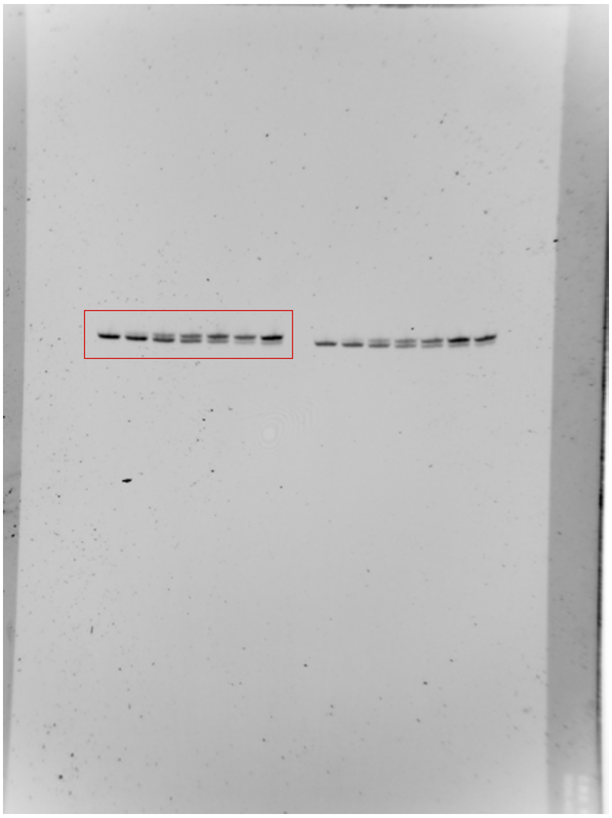

20 % dPAGE, 20×30 cm, 35W

Fig. 3f ProSeAM

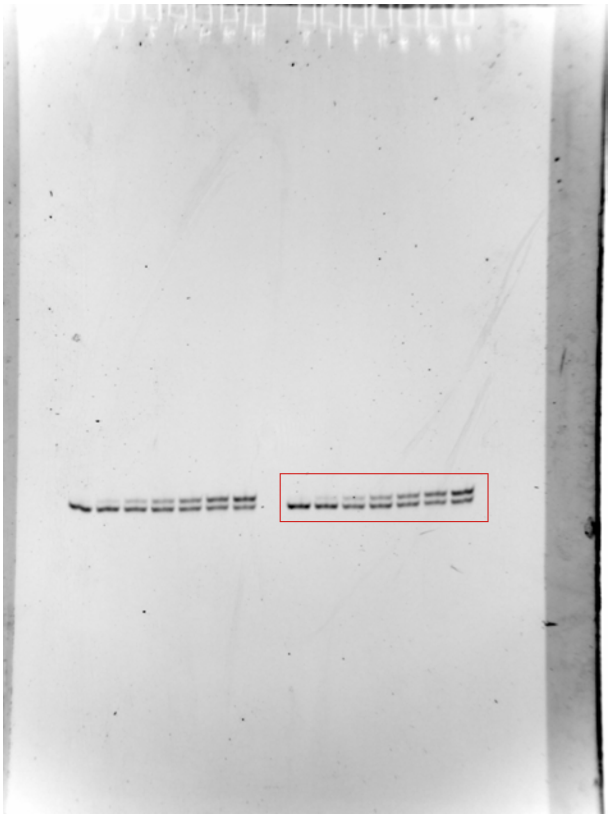

20 % dPAGE, 20×30 cm, 35W

Fig. 3f MeSeDMA

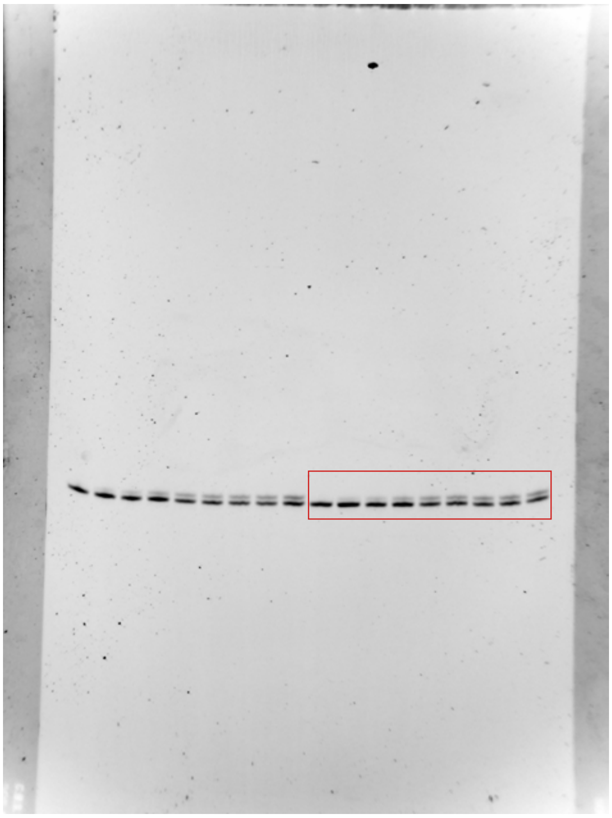

20 % dPAGE, 20×30 cm, 35W

Fig. 3f SAM

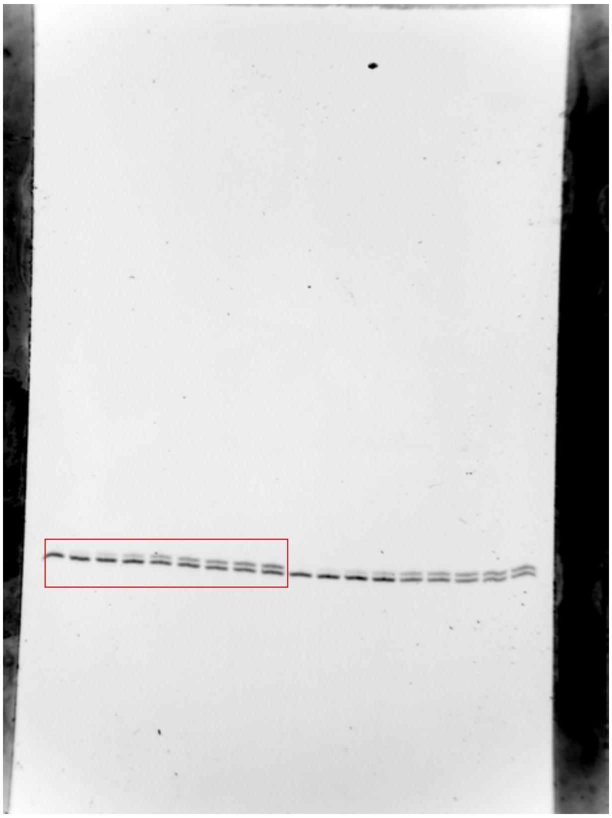

20 % dPAGE, 20×30 cm, 35W

Fig. 3f SDM

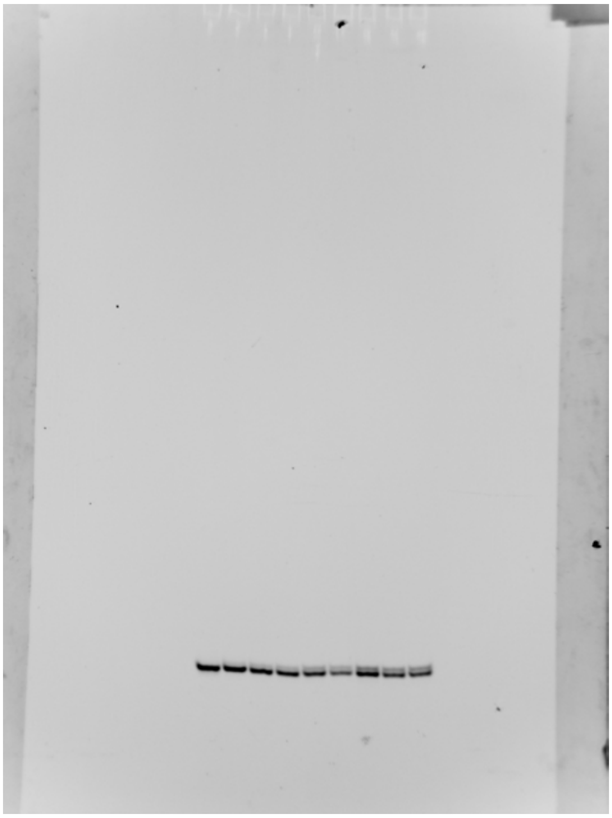

20 % dPAGE, 20×30 cm, 35W

Fig. 3g

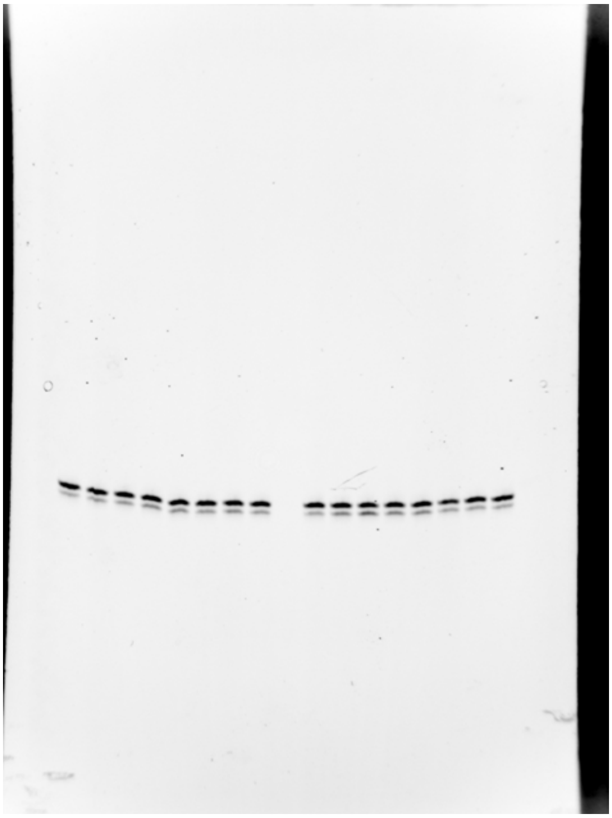

20 % dPAGE, 20×30 cm, 35W
