# Supplementary material for: A SAM analogue-utilizing ribozyme for site-specific RNA alkylation in living cells
Source: Nat Chem. 2023 Sep 4;15(11):1523–31. doi: 10.1038/s41557-023-01320-z (PMC10624628; doi:10.1038/s41557-023-01320-z)
Supplement: Supplementary file 10 — Unprocessed gels for Extended Data Fig. 3. [file 41557_2023_1320_MOESM10_ESM.pdf]

ED Fig. 3 OH2, OH 10

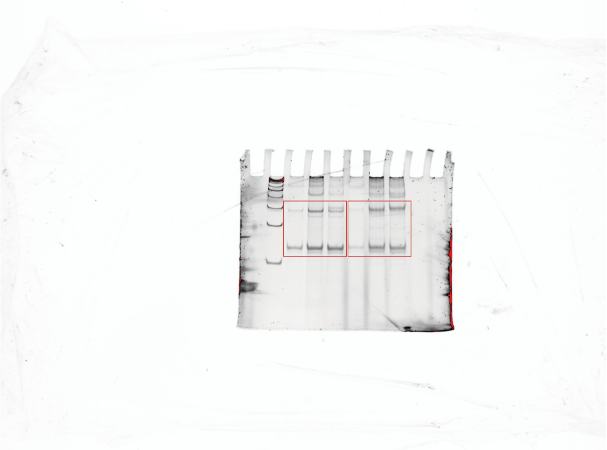

10 % nativePAGE, 10×8 cm, 200 V

ED Fig. 3 OH7, OH 9

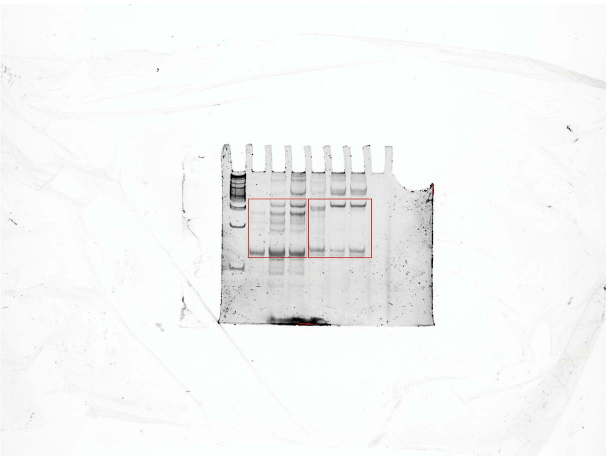

10 % nativePAGE, 10×8 cm, 200 V

ED Fig. 3 $NH_2$1, NH_2 4

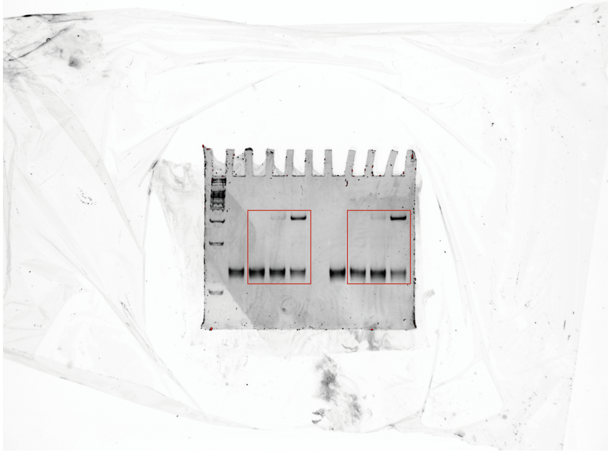

10 % nativePAGE, 10×8 cm, 200 V

ED Fig. 3 $NH_2$5, NH_2 13

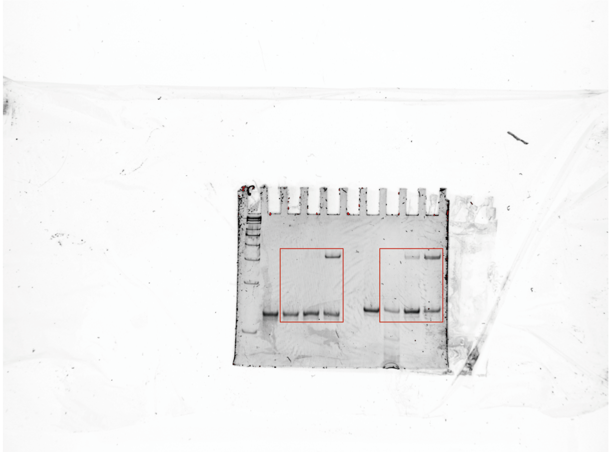

10 % nativePAGE, 10×8 cm, 200 V
