# Supplementary material for: A SAM analogue-utilizing ribozyme for site-specific RNA alkylation in living cells
Source: Nat Chem. 2023 Sep 4;15(11):1523–31. doi: 10.1038/s41557-023-01320-z (PMC10624628; doi:10.1038/s41557-023-01320-z)
Supplement: Supplementary file 11 — Unprocessed gels for Extended Data Fig. 4. [file 41557_2023_1320_MOESM11_ESM.pdf]

ED Fig.4a

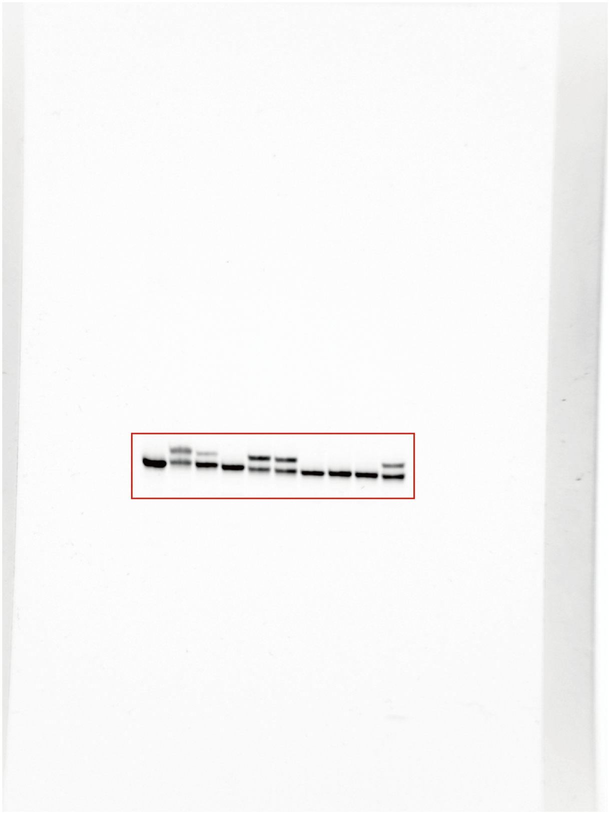

20 % dPAGE, 20×30 cm, 35W

ED Fig.4a

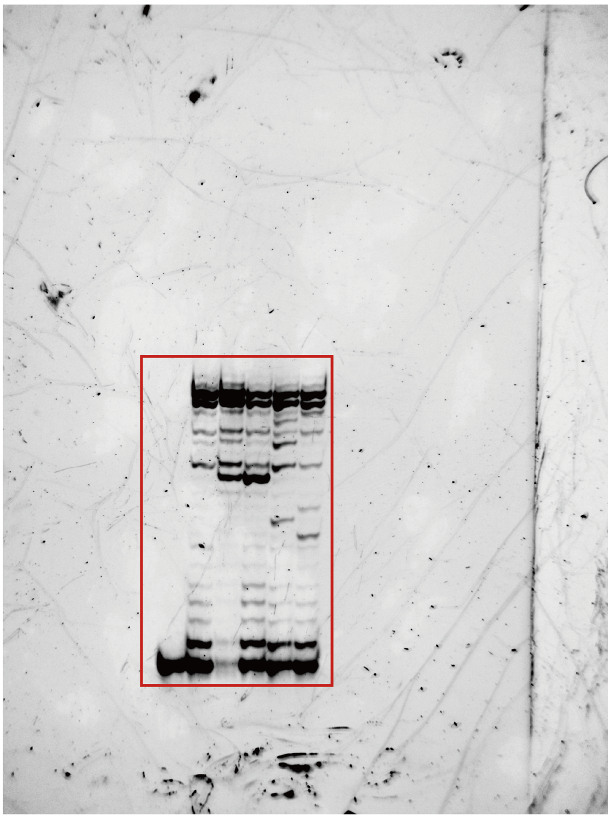

20 % dPAGE, 20×30 cm, 35W

ED Fig.4b

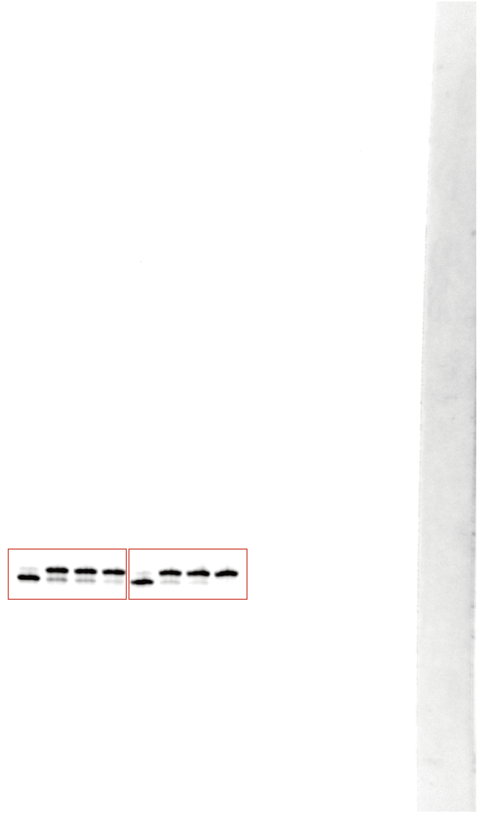

20 % dPAGE, 20×30 cm, 35W

ED Fig.4b

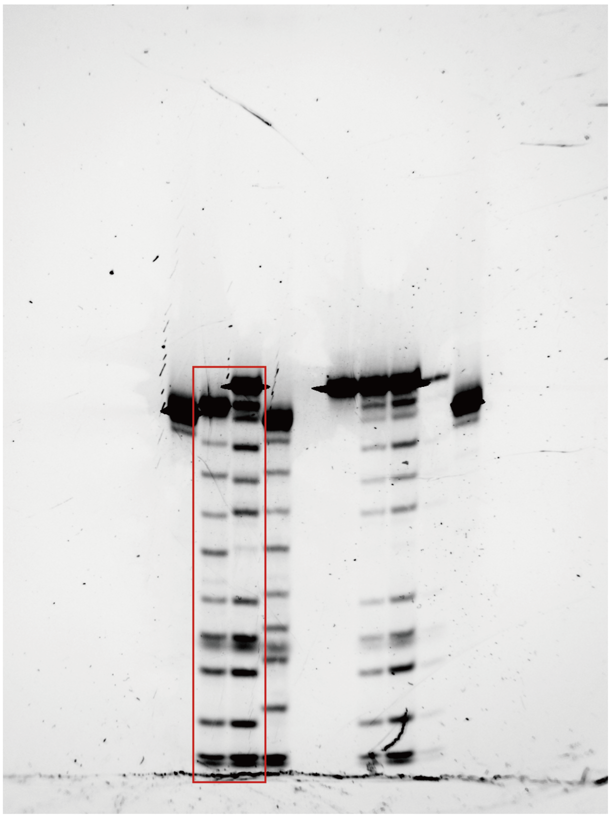

20 % dPAGE, 20×30 cm, 35W

ED Fig.4c

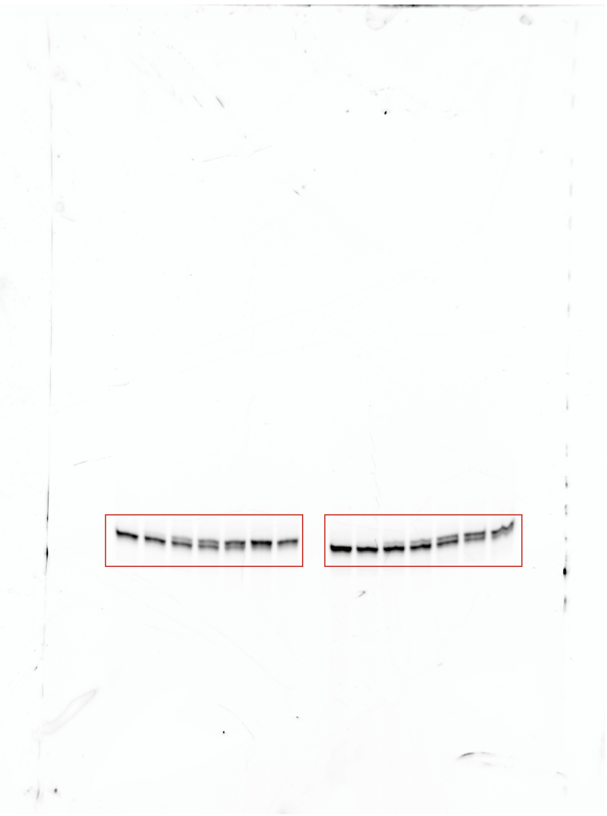

20 % dPAGE, 20×30 cm, 35W

ED Fig.4c

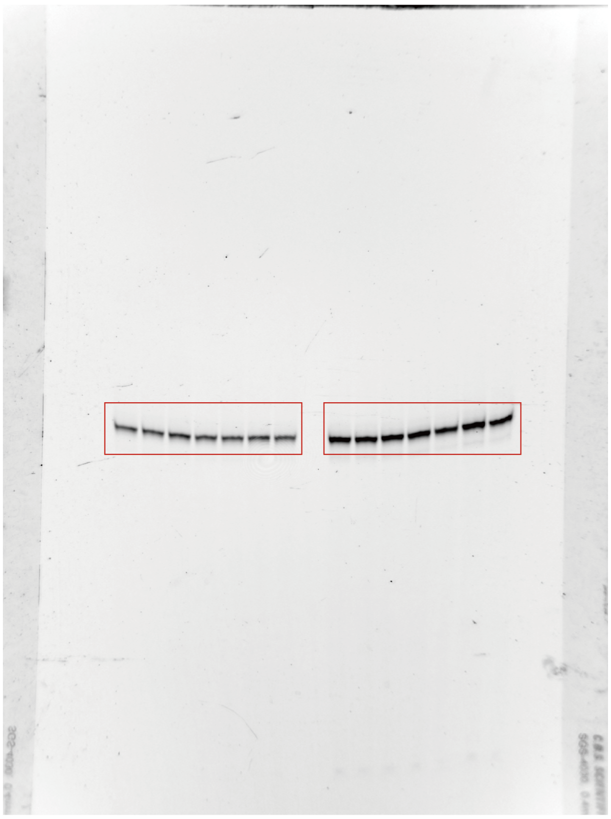

20 % dPAGE, 20×30 cm, 35W
